# Design, structure-based optimization and antiviral evaluation of potent inhibitors for the macrodomain Mac1 of SARS-CoV-2

**DOI:** 10.1038/s41467-026-75835-7

**Published:** 2026-07-27

**Authors:** Maximilian Sandmann, Sahra Tajdar, Simon Sander, David Ruiz Carrillo, Benedikt Ganter, Celine Fischer, Marina Ocenas, Stefanie Etzold, Neele Pekarek, Julia Berger, Toni Luise Meister, Barbara Selisko, Bruno Canard, Joanna M. Watt, Ondřej Baszczyňski, Barry VL Potter, Maria Garcia Alai, Henning Tidow, Susanne Pfefferle, Chris Meier, Ralf Fliegert

**Affiliations:** 1 Department of Biochemistry and Molecular Cell Biology, University Medical Center Hamburg-Eppendorf, Hamburg, Germany; 2 Organic Chemistry, Department of Chemistry, University of Hamburg, Hamburg, Germany; 3 The Hamburg Advanced Research Center for Bioorganic Chemistry (HARBOR) & Department of Chemistry, Institute for Biochemistry and Molecular Biology, University of Hamburg, Hamburg, Germany; 4 European Molecular Biology Laboratory Hamburg, Hamburg, Germany; 5 Institute for Medical Microbiology, Virology and Hygiene, University Medical Center Hamburg-Eppendorf, Hamburg, Germany; 6 Bernhard Nocht Institute, Leibniz Institute for Tropical Medicine, Hamburg, Germany; 7 Institute for Infection Research and Vaccine Development (IIRVD), Centre for Internal Medicine, University Medical Centre Hamburg-Eppendorf (UKE), Hamburg, Germany; 8 German Centre for Infection Research (DZIF), Partner site Hamburg-Lübeck-Borstel-Riems, Hamburg, Germany; 9 Laboratoire Architecture et Fonction des Macromolécules Biologiques (AFMB), CNRS, Aix-Marseille Université, UMR7257, Marseille, France; 10 Department of Life Sciences, University of Bath, Bath, UK; 11 Medicinal Chemistry & Drug Discovery, Department of Pharmacology, University of Oxford, Oxford, UK; 12 Centre for Structural Systems Biology (CSSB), DESY Campus, Hamburg, Germany

**Keywords:** Enzymes, Drug discovery, SARS-CoV-2, X-ray crystallography, Molecular conformation

## Abstract

Enzymatically active macrodomains of (+)ss-RNA viruses mediate immune evasion by countering ADP-ribosylation and are therefore promising druggable targets. Here we report testing of ADP / ADP-ribose analogues for their ability to inhibit Mac1 of SARS-CoV-2, measurement of the affinity of active compounds and characterization of their binding mode by cocrystallization, uncovering critical molecular determinants of protein-ligand interaction. Key findings of the resulting structure-activity relationship (SAR) include that inhibitory potency is improved by either replacing the distal ribose of ADP-ribose by a small alkyl group or the adenine N7 by carbon. Based on insights from the SAR, we show β-methyl-GS-441524-diphosphate as nanomolar inhibitor that exhibits >1000-fold selectivity over human MacroD1 and MacroD2. Addition of C_11_-acyloxybenzyl (AB)-masking groups yields a membrane permeable, lipophilic prodrug that inhibits SARS-CoV-2 in cell culture (EC_50_ 0.06 µM) while exhibiting low cytotoxicity (CC_50_ > 50 µM). Replacement of the terminal methyl phosphate with an ethyl phosphonate increases stability of the prodrug with little effect on toxicity and antiviral potency (EC_50_ = 0.03 µM), making it a membrane-permeable nucleotide-based prodrug against viral macrodomains.

## Abstract (web summary)

The authors report a complete structure-activity relationship of Mac1 of SARS-CoV-2, that was used to design potent nucleotide inhibitors. Hits were developed into membrane-permeable, non-toxic prodrugs, which strongly suppress viral replication.

## Introduction

Macrodomains are evolutionary conserved binding motifs for adenosine 5′-diphosphoribose (ADPR) **1**^1,2^. Some macrodomains, including the human proteins MacroD1 and MacroD2, exhibit catalytic activity and remove ADPR **1** residues from proteins^3^. ADP-ribosylation is a posttranslational modification where the ADPR moiety of β-NAD^+^ **2** is transferred to amino acid side chains of proteins, involved in a number of cellular stress responses including viral infection^4^. A number of (+)ss-RNA viruses encode MacroD-like macrodomains that were initially termed X domains because of their unknown function^5^. The discovery that these viral macrodomains are catalytically active^6^ led to the notion that they contribute to immune evasion of viruses by mediating de-ADP-ribosylation of proteins involved in sensing of viral RNA or the signalling of interferons thereby preventing the induction of interferon-stimulated genes (ISGs)^5,7^.

Coronaviruses like SARS-CoV-2 feature a conserved MacroD-like macrodomain (Mac1) as part of the transmembrane non-structural protein 3 (nsp3) encoded by open reading frame 1 (ORF1)^8,9^. Nsp3 has been reported to suppress the antiviral interferon (IFN) response triggered by viral replication intermediates upon host recognition^10^, although the exact mechanism still remains unclear. During the recent pandemic of Covid19 Mac1 received a lot of attention as potential target for antiviral therapy^11^. Numerous attempts have been made to identify inhibitors for the viral macrodomain, e.g. by high-throughput screening (HTS) and fragment screening, but so far only few viable drug candidates for Mac1 have been identified^11–20^.

Here, using an assay for the enzymatic activity of Mac1, we probed its active site using a collection of ADPR and ADP derivatives. The resulting initial SAR guided our modification of the low-affinity Mac1-binding nucleoside GS-441524 **3**^21^ into a nanomolar inhibitor β–methyl-GS-441524-diphosphate (β–methyl-GS-441524-DP) **4**. Addition of a biocleavable masking group yields a membrane permeable prodrug (ST135) **5**, that accumulates in cells to concentrations consistent with inhibition of Mac1 and dose-dependently inhibits SARS-CoV-2 in infection assays. Recombinant SARS-CoV-2 with intact or modified Mac1 enabled us to evaluate the specificity of the inhibitor. Determination of the in vitro stability of compound 5 reveals a short half-life due to spontaneous hydrolysis of the phosphate anhydride bond. Replacement of the terminal methyl phosphate with an ethyl phosphonate, yielding compound **6** (ST166), markedly improved stability.

## Results

### Fluorimetric and HPLC assay for the enzymatic activity of Mac1

The α-anomer of NAD^+^ **7** is a substrate of mono-ADP-ribosylhydrolases^22^, allowing for its use as surrogate substrate to assess their catalytic activity^23^. To determine whether this also applies to Mac1, we incubated Mac1 with both α-NAD^+^ **7** (Supplementary Fig. 1a,b) and β-NAD^+^ **2** (Supplementary Fig. 1c,d), using NADase from *Neurospora crassa* as control. The NADase hydrolyzed β-NAD^+^ **2** to produce ADPR **1** and nicotinamide **8** but did not cleave α-NAD^+^ **7**, whereas Mac1 selectively hydrolysed α-NAD^+^ **7** leaving β-NAD^+^ **2** intact. This allowed us to adopt a fluorescence microplate endpoint assay for Mac1 using a post-reaction derivatization recently employed by Wazir and colleagues^24^, that proved to be specific for the substrate and showed good robustness (Supplementary Fig. 1e,f).

**Fig. 1:**
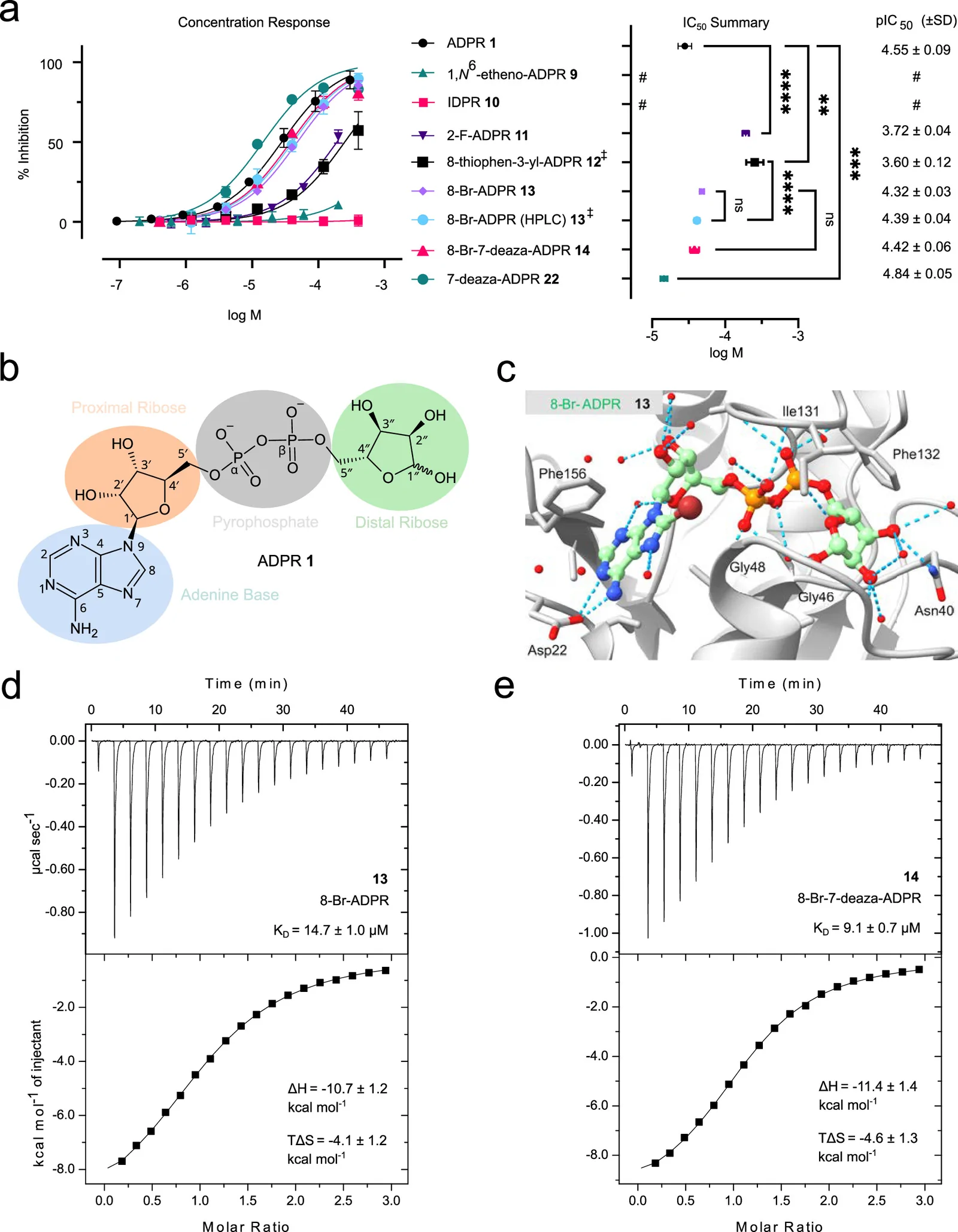
Probing the binding pocket of Mac1 using Purine-modified ADPR derivatives. **a** Concentration-responses for Mac1 inhibition by ADPR derivatives with modifications of the adenine base. Data were obtained using either the microplate assay or the HPLC assay (‡). The parameters of a sigmoidal model were fitted to the data and the derived pIC_50_ values are shown to the right. In some cases the pIC50 are outside the concentration range tested (#). Experiments using ADPR **1** (black) as inhibitor were always included as control and are shown for comparison. Data are presented as mean ± SD and were tested by one-way ANOVA followed by pair-wise comparison using Šídák’s correction. For all compounds except ADPR **1**, data are from 3 independent experiments. Since ADPR **1** was always included as matched control, data are from 6 independent experiments. ns: not significant, ** *p* ≤ 0.01,*** *p* ≤ 0.001, **** *p* ≤ 0.0001. Adjusted *p*-values: **1** vs. **11**: <0.0001, **1** vs. **13**: 0.0017, **13** (plate) vs. **13** (HPLC): 0.843, **12** vs. **13**: <0.0001, **13** vs. **14**: 0.4856, **1** vs. **22**: 0.0002. **b** Functional moieties of ADPR **1**. **c** Structure of 8-Br-ADPR **13** (PDB 8AZM) in complex with Mac1. H-bond interactions are shown as dashed blue lines. **d**, **e** ITC data for the binding of 8-Br-ADPR **13** (**d**) and 8-Br-7-deaza-ADPR **14** (**e**) to Mac1. Top graphs show thermograms and bottom graphs depict the integrated values of each titration point fitted to a one-site binding model. The experiment was repeated two more times yielding K_D_, ΔH and TΔS as mean ± SD. Source data are provided as a Source Data file.

As the product of the α-NAD^+^ **7** hydrolysis reaction, ADPR **1** inhibited Mac1 with an IC₅₀ of ~28 µM (pIC_50_ 4.55, Fig. 1a), as described for the homologous macrodomains MacroD1 and MacroD2^24^, which was in good agreement with the binding determined by X-ray crystallography and isothermal titration calorimetry (ITC, Supplementary Figs. 1g and 2). Of note, the physiological ligand ADPR **1** consists of an adenine base, a proximal ribofuranose, a pyrophosphate and a distal reducing ribose (Fig. 1b), thus we explored the active site using ADPR and ADP analogs with targeted modifications in each structural component (Supplementary Figs. 3, 4and 5, Supplementary Tables 1 and 2), using ADPR **1** as point of reference.

### Probing the adenosine binding pocket

Structural superposition of our own high-resolution crystal structure of Mac1 in complex with ADPR **1** (PDB: 8AZD) with existing structures of Mac1-ADPR complexes (PDB 6Z5T, 7KQP, 6WOJ, 6W02) demonstrates a conserved macrodomain fold and ligand binding mode, with two key hydrogen bonds between the adenine base and the protein: N6 acts as hydrogen bond donor to Asp22, while N1 accepts a hydrogen bond from the amide N-H of the peptide bond between Asp22 and Ile23. To address the importance of these interactions we tested 1,*N*⁶-ethenoadenosine-5′-*O*-diphosphoribose (1,*N*^6^-etheno-ADPR) **9**, where the etheno-bridge sterically prevents formation of both hydrogen bonds, and inosine-5′-*O*-diphosphoribose (IDPR) **10** where the exocyclic amino group is replaced by a keto group and N1 no longer accepts hydrogen bonds because its electron lone pair has become part of the aromatic heterocycle. Both compounds did not inhibit Mac1 (Fig. 1a).

Introduction of a fluorine at C2 (2-F-ADPR) **11** significantly decreased inhibition (pIC_50_ 3.72) compared to ADPR **1**. The electronegative fluorine reduces N1 basicity (in adenosine addition of a 2-F shifts the pK_a_ from 3.5 to <1)^25^ weakening the hydrogen bond between N1 and Asp22.

Addition of a thiophen-3-yl group to C8 (8-thiophen-3-yl-ADPR) **12** significantly reduced inhibition (pIC₅₀ 3.60). To investigate whether this effect depends on the size of the substituent, brominated ADPR analogs were tested, given the small size of bromine compared to the larger thiophen residue. Both 8-Br-ADPR **13** and 8-Br-7-deaza-ADPR **14**, inhibited Mac1 more potently than 8-thiophen-3-yl-ADPR **12** (pIC₅₀ 4.32 and 4.42, respectively), suggesting that bulky C8-substituents negatively affect inhibitory potential.

8-substitutions in purines shift the equilibrium between the *syn* and *anti*-conformation around the *N*-glycosidic bond in favor of the *syn-*conformation^26,27^. We solved the structure of 8-Br-ADPR **13** in complex with Mac1 (PDB 8AZM) showing the adenine base of **13** in an *anti*-conformation assuming an overall ADPR-like binding pose (Fig. 1c, Supplementary Fig. 6a), with the bromine pointing out of the binding pocket and no obvious steric clashes. Binding assays (Fig. 1d, Supplementary Table 2) showed a lower affinity for 8-Br-ADPR **13** (14.7 µM) than for ADPR **1**, consistent with its reduced inhibitory potency. 8-Br-7-deaza-ADPR **14** exhibited a K_D_ comparable to ADPR **1** (Fig. 1e). Both 8-brominated analogs exhibited slightly higher net binding enthalpies than ADPR **1**, which in case of 8-Br-ADPR **13** only partially compensated for the substantial entropic penalty associated with the C8-substituent, whereas in case of 8-Br-7-deaza-ADPR **14** the higher binding enthalpy compensated the entropic penalty due to the C8-substitution.

**Fig. 2:**
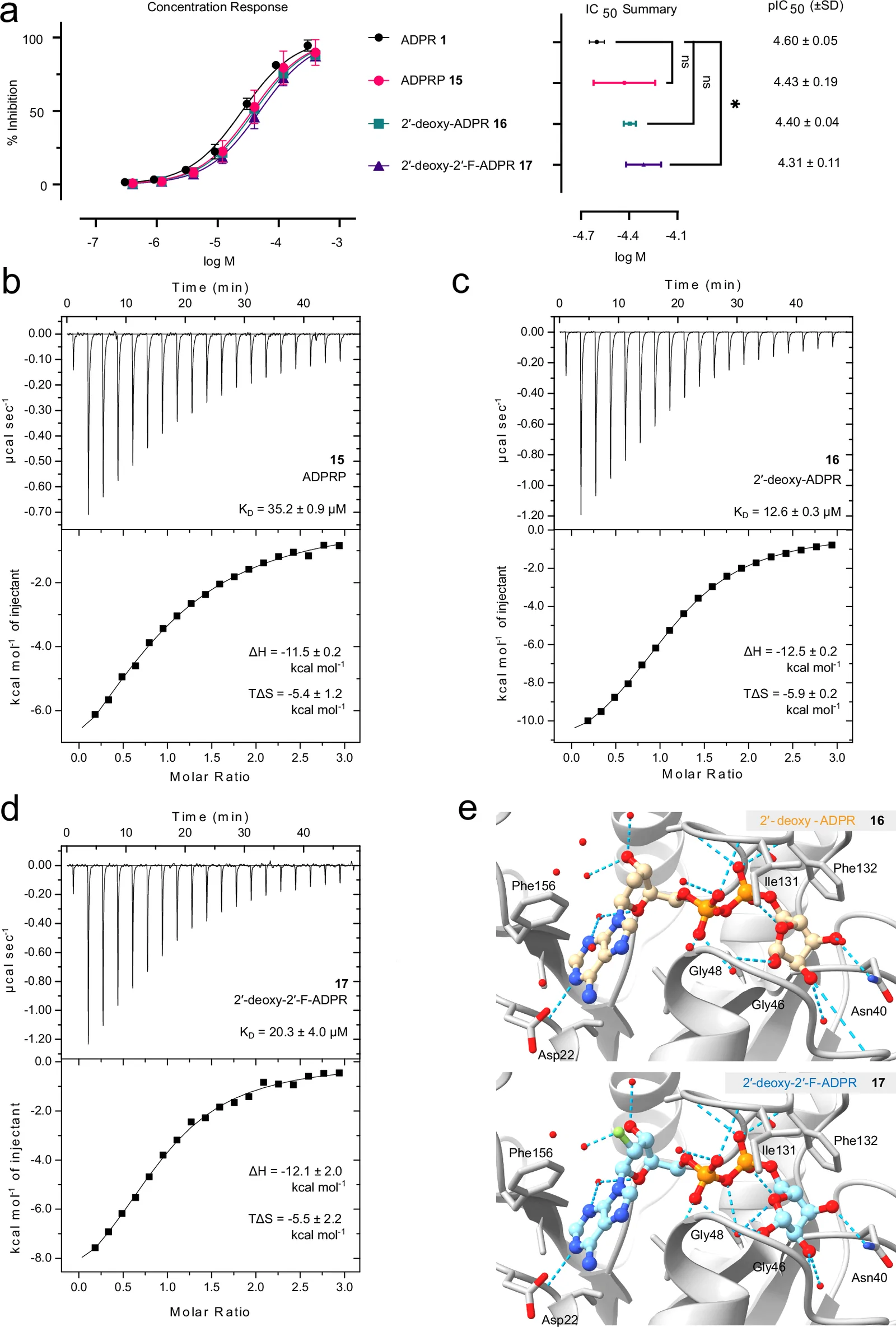
Modifications at the C2′ position of the proximal ribose of ADPR have no major impact on affinity and inhibition of Mac1. **a** Concentration-responses for Mac1 inhibition by ADPR derivatives with modifications of the adenosine ribose. Data were obtained using the microplate assay. The parameters of a sigmoidal model were fitted to the data and the derived pIC_50_ values are shown to the right. Experiments using ADPR **1** (black) as inhibitor were included as control and are shown for comparison. Data are presented as mean ± SD from 3 independent experiments and were tested by one-way ANOVA followed by pair-wise comparison using Šídák’s correction. ns: not significant, *p ≤ 0.05. Adjusted *p*-values: **1** vs. **15**: 0.2802, **1** vs. **16**: 0.1678, **1** vs. **17**: 0.0423. Representative ITC data for the binding of ADPRP **15** (**b**), 2´-deoxy-ADPR **16** (**c**) and 2´-deoxy-2´-F-ADPR **17** (**d**) to Mac1. Top graphs show thermograms and the bottom graphs depict the integrated values of each titration point fitted to a one-site binding model. The experiments were repeated two more times yielding K_D_, ΔH and TΔS as mean ± SD. **e** Structure of 2´-deoxy-ADPR **16** (beige, PDB 8AZI) and 2´-deoxy-2´-F-ADPR **17** (turquoise, PDB 8AZL) in complex with Mac1. H-bond interactions are shown as dashed blue lines. Source data are provided as a Source Data file.

The 2′-hydroxyl group of ADPR **1** points out of the pocket without direct interactions with the protein (PDB 8AZD, Supplementary Fig. 2a-c). Ni and coworkers have shown that ADPR-2′-phosphate (ADPRP) **15** binds to Mac1 with slightly higher affinity than ADPR **1**^21^. Mac1 bound to ADPRP **15** (PDB 7BF5) revealed an Mg^2+^ ion-mediated contact with Asp157^21^. In our hands, ADPRP **15** did not significantly differ in inhibitory potency from ADPR **1** (Fig. 2a), though ITC measurements showed lower affinity (35.2 µM, Fig. 2b).

In solution the ribofuranose of ribonucleotides mostly assumes the 3′-endo conformation but is in rapid equilibrium with the 2′-endo configuration^28,29^. Enzymes can show preference for a specific pucker of the substrate^30^. We tested 2′-deoxy-ADPR **16** which preferentially assumes the 2′-endo conformation and 2′-deoxy-2′-F-ADPR **17** where fluorine locks the sugar in the 3′-endo conformation^31^ (Fig. 2a). 2′-Deoxy-ADPR **16** did not differ significantly from ADPR **1** in inhibitory potency, and 2′-deoxy-2′-F-ADPR **17** exhibited only slightly decreased inhibitory potency (pIC_50_ 4.31) compared to ADPR **1** (Fig. 2a). This is also reflected by the slightly lower affinity of both compounds (12.6 µM and 20.3 µM respectively, Fig. 2c, d), indicating that Mac1 does not favor one ribofuranose pucker. However, the resolution of the structure of both compounds in complex with Mac1 (PDB 8AZI 1.9 Å and 8AZL 2.2 Å, Fig. 2e and Supplementary Fig. 6b, c) was insufficient to determine unambiguously the pucker of the ribofuranose.

Additionally, we tested commercial ADP derivatives (Supplementary Figs. 4 and 7). ADP **18** showed weak inhibition (pIC₅₀ 2.44). As in ADPR **1**, removal of the C2′ hydroxyl group (2′-deoxy-ADP **19**) had little effect. Replacing N7 with a carbon significantly enhanced inhibition for both 7-deaza-ADP **20** (pIC₅₀ 2.92) and 7-deaza-2′-deoxy-ADP **21** (pIC₅₀ 2.57), which led us to hypothesise that the 7-deaza modification generally increases affinity and inhibitory potency. To test this hypothesis, we synthesized 7-deaza-ADPR **22** adapting published protocols^32–37^. 7-deaza-ADPR **22** turned out more potent than ADPR **1** (pIC_50_ 4.84, Fig. 1a) illustrating again the importance of the N1 to Asp22 hydrogen bonding.

### Probing the binding pocket for the distal ribose

The distal ribose is coordinated through multiple hydrogen bonds (PDB 8AZD, Supplementary Fig. 2a-c). The 3″-hydroxyl group hydrogen bonds with Asn40, while the 1″-hydroxyl group engages in hydrogen bonding with a catalytic water also bound to the α-phosphate. Rijpkema and coworkers demonstrated that modifications at C2″ and C3″ of the distal ribose reduced the ability to inhibit Mac1, whereas introduction of a cyano group at C1″ in the α-configuration increased affinity and inhibitory potency^38^. Additionally, mutation of Asn40 inactivates Mac1 supporting the importance of the 3″-hydroxyl group for the recognition of the distal ribofuranose^8,39^.

**Fig. 3:**
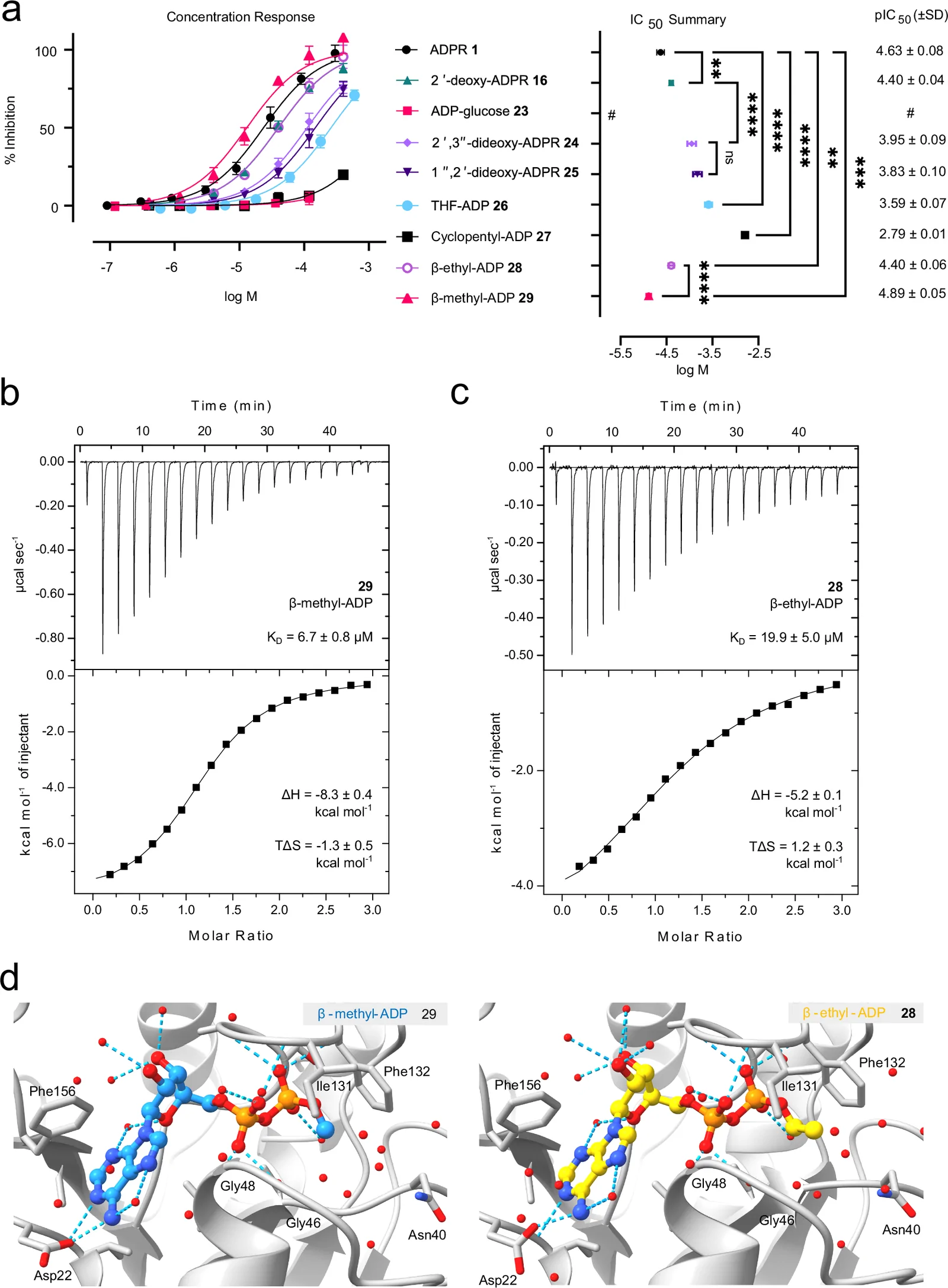
Substitution of the distal ribose of ADPR by small alkyl groups improves affinity and inhibition of Mac1. **a** Concentration-responses for Mac1 inhibition by ADPR derivatives with modifications of the distal ribose. All data were obtained using the microplate assay. The parameters of a sigmoidal model were fitted to the data and the derived pIC_50_ values are shown to the right. In some cases, the pIC_50_ are outside the concentration range tested (#). Matched experiments using ADPR **1** (black) and 2´-deoxy-ADPR **16** (green) as inhibitor were included as control and are shown for comparison. Data are presented as mean ± SD and were tested by one-way ANOVA followed by pair-wise comparison using Šídák’s correction. For all compounds except ADPR **1**, data are from 3 independent experiments. Since ADPR **1** was always included as matched control, data are from 5 independent experiments. ns: not significant, ** *p* ≤ 0.01,*** *p* ≤ 0.001, **** *p* ≤ 0.0001. Adjusted p-values: **1** vs. **16**: 0.0022, **16** vs. **24**: <0.0001, **1** vs. **26**: <0.0001, **1** vs. **27**: <0.0001, **1** vs. **29**: 0.0007, **1** vs. **28**: 0.0023, **28** vs. **29**: <0.0001, **24** vs. **25**: 0.293. ITC data for the binding of β-methyl-ADP **29** (**b**) and β-ethyl-ADP **28** (**c**) to Mac1. The top graphs show thermograms and the bottom graphs show the integrated values of each titration point fitted to a one-site binding model. The experiments were repeated two more times yielding K_D_, ΔH and TΔS as mean ± SD. **d** Structure of β-ethyl-ADP **28** (PDB 8AZO) and β-methyl-ADP **29** (PDB 8AZP) in complex with Mac1. H-bond interactions are shown as dashed blue lines. Source data are provided as a Source Data file.

To address the role of the distal ribose, we tested ADP-glucose **23**, with the distal ribose replaced by D-glucose (Fig. 3a). ADP-glucose **23** showed no detectable inhibition, indicating that steric bulk and/or increased polarity due to the additional hydroxyl-group at this position is unfavorable. Furthermore we tested ADPR analogs with progressively simpler structures, lacking one or more hydroxyl groups at the distal ribose. 2′,3″-Dideoxy-ADPR **24** exhibited reduced inhibitory potency compared to 2′-deoxy-ADPR **16** (pIC_50_ 3.95 vs. 4.40, respectively) (Fig. 3a), consistent with the hydrogen bond between the 3″-hydroxyl group and Asn40 and the inactivity of the Asn40 mutant^8,39^. Removal of the 1″-hydroxyl group in 1″,2′-dideoxy-ADPR **25** further reduced inhibitory potency (pIC_50_ 3.83) (Fig. 3a).

To explore whether the distal ribose could be replaced by non-hydrogen-bonding moieties, we tested β-(2-tetrahydrofuranyl)-ADP (THF-ADP) **26**, an ADPR **1** derivative lacking all hydroxyl groups of the five-membered ring. THF-ADP **26** exhibited a significantly lower inhibitory potency than ADPR **1** (pIC_50_ 3.59, Fig. 3a). Replacing the distal ribose by cyclopentane led to an even greater loss of inhibition (β-cyclopentyl-ADP **27**, pIC_50_ 2.79) (Fig. 3a). Interestingly, substituting the distal ribose with smaller alkyl chains resulted in pIC_50_ values of 4.40 for β-ethyl-ADP **28**, which is somewhat less potent than ADPR **1**, and 4.89 for β-methyl-ADP **29**, which makes this compounds a more potent inhibitor than ADPR **1** (Fig. 3a). This is in contrast to the low inhibitory potency of ADP **18**. ITC confirmed that β-methyl-ADP **29** has a higher affinity than ADPR **1** (6.7 µM vs 10.2 µM respectively), while β-ethyl-ADP **28** exhibited a somewhat lower affinity (19.9 µM) than ADPR **1** (Fig. 3b, c).

Our structures of β-ethyl-ADP **28** (PDB 8AZO) and β-methyl-ADP **29** (PDB 8AZP) in complex with Mac1 show the alkyl chains in close proximity to the isoleucine and phenylalanine of the GIF motif in loop 2 (Fig. 3d and Supplementary Fig. 8). Replacement of these residues by alanine has been shown to abrogate catalytic activity while concomitantly increasing affinity for ADPR **1** (Supplementary Fig. 9a,b)^40^. To test whether interactions between Phe132 and the methyl group contribute to the higher affinity for the alkylated ADP derivatives, we expressed Mac1 Phe132Ala and determined the affinity for ADPR **1**, β-ethyl-ADP **28** and β-methyl-ADP **29**. As expected the affinity of Mac1 Phe132Ala for ADPR **1** (4.9 μM) was higher than that of Mac1 (10.2 µM). Unexpectedly both β-alkylated-ADP **28** and **29** also bound with even higher affinity to Mac1 Phe132Ala (3.3 µM and 2.9 µM respectively) (Supplementary Fig. 9c,d) than to Mac1 (Fig. 3b, c).

### Bioisosteric replacement of the pyrophosphate

Nucleoside diphosphate analogs face several challenges as inhibitors of intracellular enzymes. Their charged pyrophosphate limits membrane permeability and renders them susceptible to hydrolysis, reducing stability in biological fluids. Replacing the pyrophosphate with a more stable, less charged bioisostere could therefore enhance the potential of ADPR analogs as Mac1 inhibitors^41^.

We tested a series of compounds with the pyrophosphate replaced by bioisosteres commonly used for diphosphates in drug design: 5-ribosyl-squaryl-adenosine **30**^42^, adenosine-5′-*O*-(2-phosphoryl)acetate ribose (A-acetyl-PR) **31** and adenosine-5′-phosphonoacetyl-ribose (AMP-acetyl-R) **32**^43^ as well as α-β methylene-ADPR (CH_2_-ADPR) **33** and α-β methylene-ADP (CH_2_-ADP) **34** (Supplementary Figs. 3). However, none of these compounds inhibited Mac1 over the tested concentration range (Supplementary Fig. 10).

Another approach to enhance stability involves replacing the non-bridging oxygens by sulphur^41^. Phosphorothioates have successfully been used to increase the stability of oligonucleotides^44^. We tested adenosine-5′-*O*-(2-thiodiphosphate) (ADP-β-S) **35**, along with **(***R*p)-ADP-α-S **36** and (*S*p)-ADP-α-S **37** (Supplementary Figs. 5 and 10). While ADP-β-S 35 exhibited inhibitory potency comparable to ADP (pIC_50_ 2.41), both *R*p-ADP-α-S **36** and *S*p-ADP-α-S **37** showed enhanced inhibition of Mac1. Notably, stereoselectivity was observed, with the *S*p-isomer **37** showing significantly greater inhibition (pIC_50_ 2.75) than the *R*p-isomer **36** (pIC_50_ 2.59) (Supplementary Fig. 10).

### Key Mac1-ligand interactions: a structure-activity relationship (SAR) model

**Fig. 4:**
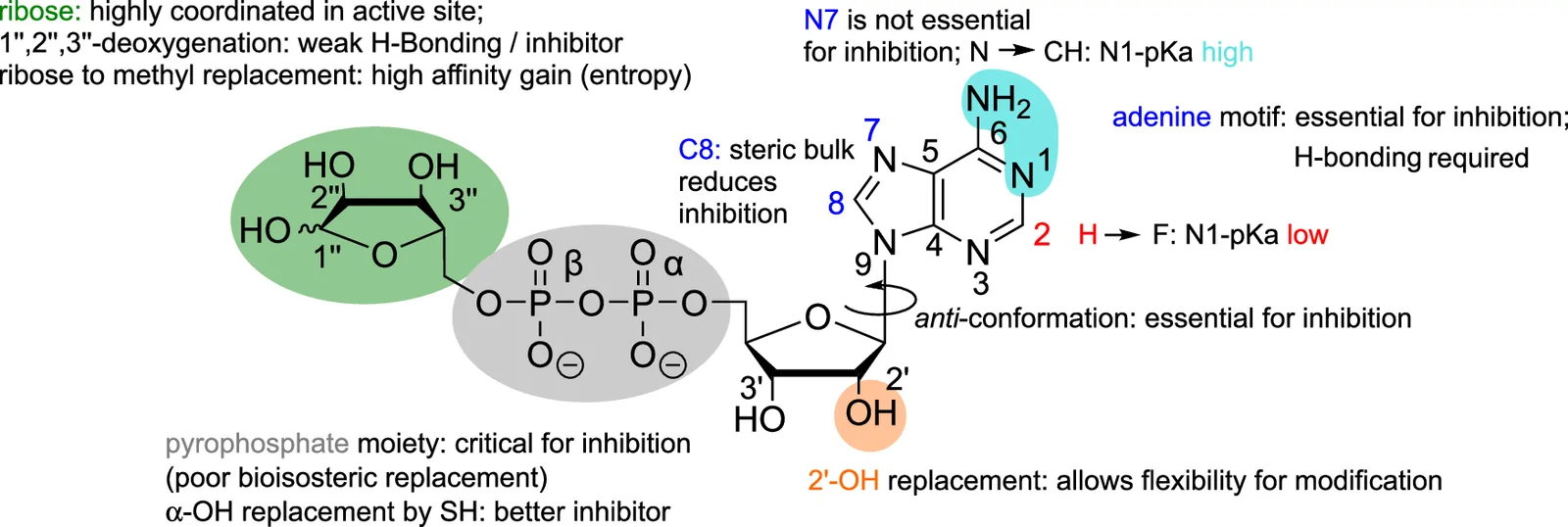
Major SAR findings for ADPR 1 binding towards Mac1. Summary of the major structure–activity relationship (SAR) findings for ADP(R)-derived ligands. Structural modifications of the adenine base, the proximal ribose, the pyrophosphate (grey), and the distal ribose (green) that affect inhibitory activity and/or binding affinity are highlighted. N1 and the exocyclic amino group of the adenine base (cyan) form hydrogen bonds with Mac1 that can be modulated by modification of N7 and C2. The 2′-hydroxyl group (orange), while not significantly affecting inhibitory potency, might allow further modifications.

Major findings from probing the active site of Mac1 with our ADP(R) library are summarized in Fig. 4. Our SAR model gives a comprehensive overview of the important biophysical, chemical and sterical features of the different functional groups of ADPR **1**, i.e. the adenosine moiety, the pyrophosphate and the distal ribose that determine ligand engagement by Mac1, which could be exploited for subsequent ligand-based drug design approaches.

### SAR-guided design of a nanomolar inhibitor for Mac1

**Fig. 5:**
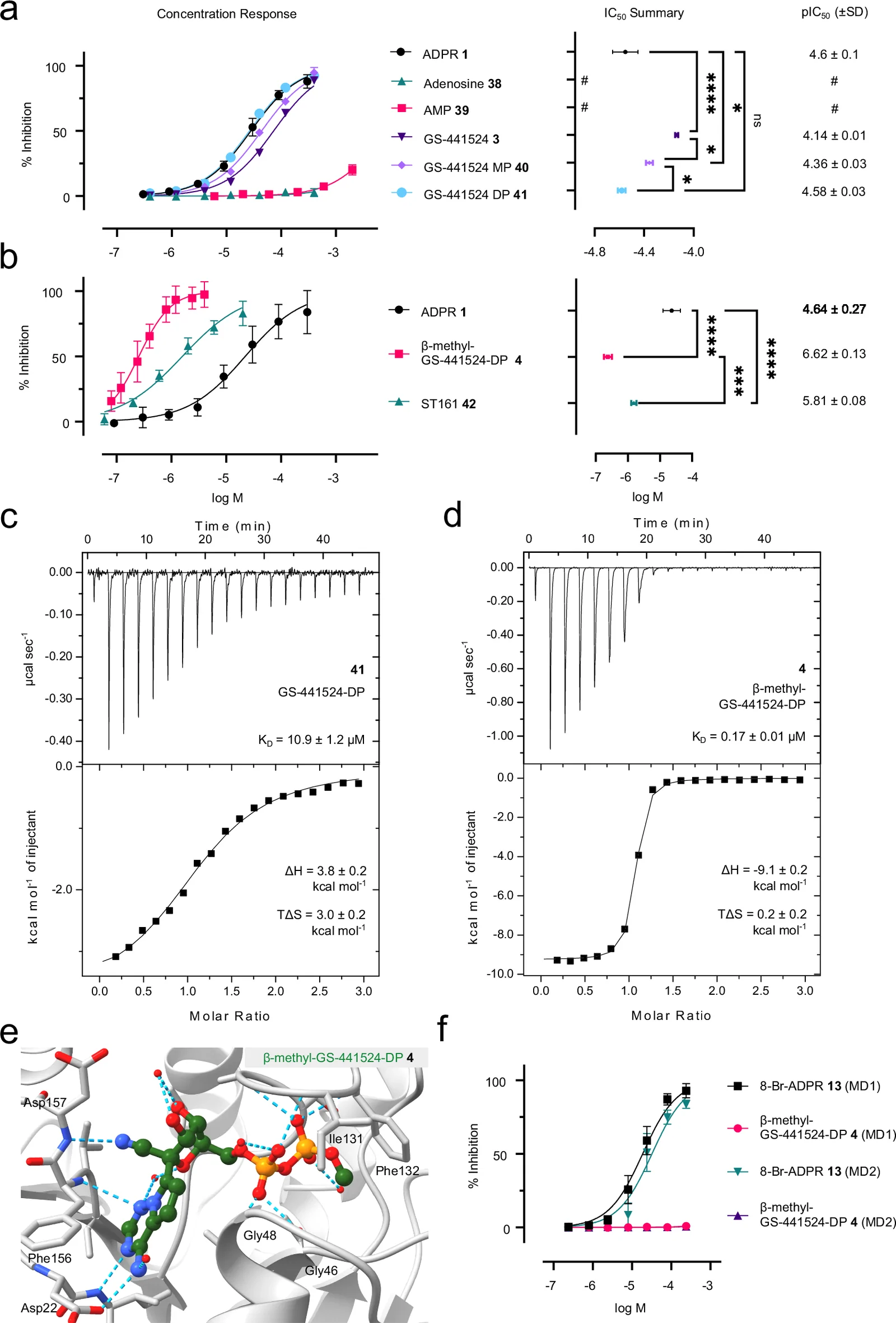
Development of nucleotide-based Mac1 inhibitors β-methyl-GS-441524-DP and ST161. Concentration-responses for Mac1 inhibition by (**a**) adenosine **38**, AMP **39**, GS-441524 **3** and its mono- **40** and diphosphate **41** as well as (**b**) β-methyl-diphosphate **4** and β-ethylphosphonate phosphate derivatives (ST161) **42** of GS-441524 obtained by microplate assay. All data are shown as mean ± SD (**a** from 3 independent experiments, except ADPR **1:** 9 independent experiments, **b** from 6 independent experiments for β-methyl-GS-441524-DP **4** and ADPR **1**, ST161 **42:** 3 independent experiments). Response curves were fitted by sigmoidal models and pIC_50_ shown to the right. #: pIC_50_ outside the concentration range tested. pIC_50_s were tested by one-way ANOVA followed by pair-wise comparison using Šídák’s correction. ns: not significant, *p ≤ 0.05, *** *p* ≤ 0.001, **** *p* ≤ 0.0001. Adjusted *p*-values: **1** vs. **3**: <0.0001, **1** vs. **40**: 0.0117, **1** vs. **41**: 0.9886, **3** vs. **40**: 0.0202, **40** vs. **41**: 0.0184, **1** vs. **4**: <0.0001, **1** vs. **42**: <0.0001, **4** vs. **42**: 0.0002. Representative ITC data for Mac1 binding of GS-441524-DP **41** **c** and β-methyl-GS-441524-DP **4** **d** (three independent experiments each). Top graphs show thermograms and bottom graphs show the integrated values of each titration point fitted to a one-site binding model. Calculated K_D_, ΔH and TΔS shown as mean ± SD. **e** Structure of β-methyl-GS-441524-DP **4** (green, PDB 9RHO) in complex with Mac1 with H-bond interactions (dashed blue lines). **f** Response curves for inhibition of human MacroD1 (MD1) and MacroD2 (MD2) by β-methyl-GS-441524-DP **4** and 8-Br-ADPR **13** (control). Data obtained by microplate assay are presented as mean ± SD and were fit to a sigmoidal model (β-methyl-GS-441524-DP **4:** 3 independent experiments, 8-Br-ADPR **13:** 6 independent experiments). Source data are provided as a Source Data file.

GS-441524 **3**, the nucleoside of the antiviral prodrug Remdesivir, binds to Mac1 with an affinity similar to ADPR **1**^21^. As adenosine **38** and AMP **39** showed no inhibition (Fig. 5a), we tested whether GS-441524 **3** inhibited Mac1 activity, which it did, although with significantly lower potency than ADPR **1** (pIC_50_ 4.14) (Fig. 5a), as reported previously^45^. Phosphorylation of GS-441524 **3** increased potency, with a pIC_50_ of 4.36 for the GS-441524-monophosphate (MP) **40** and its diphosphate (DP) **41** being equally potent (pIC_50_ 4.58) to ADPR **1** (Fig. 5a). The difference to the very low potency of ADP **18** is due to the replacement of the adenine by 4-amino-pyrrolo[2,1-*f*][1,2,4]triazin and the additional cyano group at C1´.

Based on our SAR model, which suggests favoured binding of Mac1 towards β-alkylated nucleotide diphosphates (Fig. 4), we synthesized β-methyl-GS-441524-DP **4**. Converting the β-phosphate into a methyl ester had a similar effect on GS-441524-DP **41** as it had on ADP **18**, increasing inhibitory potency and affinity by two orders of magnitude (pIC_50_ 4.58 and 10.9 µM for GS-441524-DP **41** and pIC_50_ 6.62 and 0.17 µM for β-methyl-GS-441524-DP **4**) (Fig. 5b-d). Furthermore, the structure of Mac1 in complex with β-methyl-GS-441524-DP **4** (PDB 9RHO, Fig. 5e) shows an ADPR-like binding mode (Supplementary Fig. 11) almost identical to β-methyl-ADP **29** (PDB 8AZP, Fig. 3d).

### Effect of Mac1 inhibitors on human MacroD1 and MacroD2

It is important that drugs against viral macrodomains do not inhibit human orthologs^46^. We thus tested β-methyl-GS-441524-DP **4** against human MacroD1 and MacroD2. As 8-Br-ADPR **13** inhibits both MacroD1 and MacroD2 in the low micromolar range, it was included as control. β-methyl-GS-441524-DP **4** did not affect either enzyme over the concentration range tested (Fig. 5f).

### Development of a prodrug for cellular studies

**Fig. 6:**
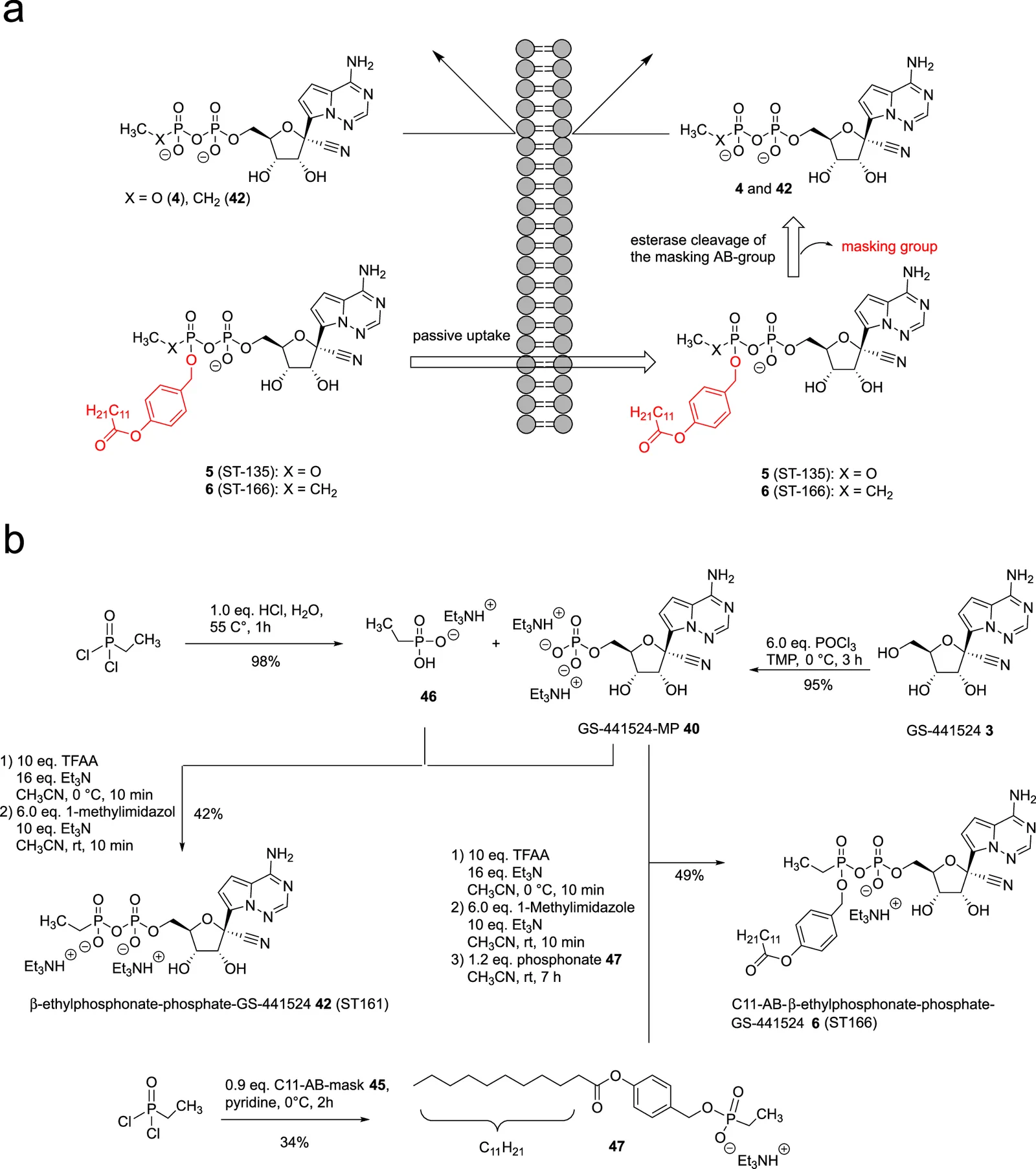
The Mac1 prodrug concept. Scheme of the **a** intracellular delivery and entrapment of nucleotides as C_11_-AB-masked prodrugs as well as for the **b** synthesis of ST166 **6** and its parent β-ethylphosphonate-phosphate-GS-441524 **42** (ST161).

Since bioisosteric replacement was ineffective, we attempted to convert the compound into a prodrug. Methods were developed to allow membrane passage of biocleavably-modified nucleotides^47–50^ by adding covalently chemical moieties to the terminal phosphate making the compound lipophilic enough for cell permeation (Di*PP*ro- and Tri*PPP*ro-approaches). The bipartite acyloxybenzyl (AB) prodrug groups get enzymatically cleaved by esterases or lipases. For the conversion of β-methyl-GS-441524-DP **4** into the respective prodrug ST135 **5** the Di*PP*ro approach was used^51,52^ (Fig. 6).

**Fig. 7:**
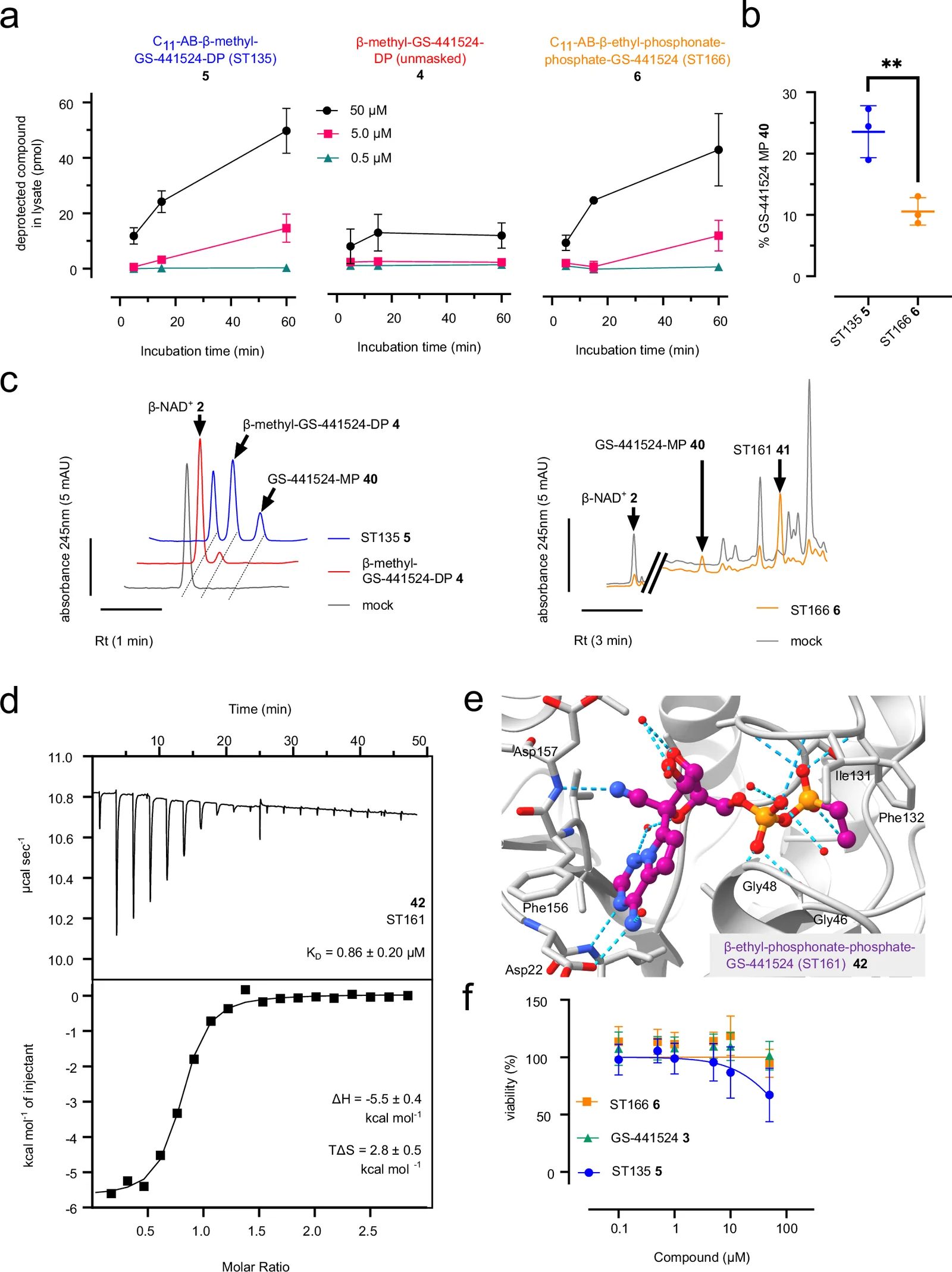
Permeability, metabolic stability and cytotoxicity of the prodrugs of β-methyl-GS-441524-DP and ST161. **a**–**c** Uptake and metabolic hydrolysis of the AB-masked prodrugs ST135 **5** and ST166 **6** and β-methyl-GS-441524 DP **4** by CaLu-3 cells (from 3 independent experiments). (a) Amounts of the respective deprotected compounds (in case of the prodrugs) or unmasked β-methyl-GS-441524-DP **4** in cell lysates determined by RP-HPLC shown as mean ± SD. **b** Breakdown of prodrug **5** and **6** after 1 h of CaLu-3 treatment with 50 µM compound. The amounts of GS-441524-MP **40** were normalized to the sum of GS-441524-MP **40** and the respective deprotected compound with intact pyrophosphate (β-methyl-GS-441524-DP **4** or ST161 **42**) in the lysates. Data as mean ± SD tested by two-sided unpaired t-test, ** p ≤ 0.01. Exact *p*-value: 0.0093. (c) Representative HPLC data for (a,b). Chromatograms display 1 h of treatment with 50 µM of either (left panel) the C_11_-AB-prodrug of β-methyl-GS-441524-DP **5** (ST135, blue), β-methyl-GS-441524-DP **4** (red) or (right panel) the prodrug ST166 **6** (orange). Vehicle control shown in grey. Rt: retention time. **d** Representative ITC data for the binding of ST161 **42** to Mac1 (from three independent experiments). The top graph shows the thermogram and the bottom graph shows the integrated values of each titration point fitted to a one-site binding model. Calculated K_D_, ΔH and TΔS shown as mean ± SD. **e** Structure of ST161 **42** (PDB 9RHN) in complex with Mac1 with H-Bonds (Dashed blue lines). **f** Cytotoxicity of GS-441524 **3** (green), the C_11_-AB-prodrug of β-methyl-GS-441524-DP (ST135) **5** (blue), and ST166 **6** (orange). Cell viability calculated in relation to untreated cells. Data shown mean ± SD from 4 (ST135 **5** and GS-441524 **3**) or 3 independent experiments (ST166 **6**) and fitted to sigmoidal model to derive CC_50_. Source data are provided as a Source Data file.

Significantly more β-methyl-GS-441524-DP **4** accumulated in CaLu-3 cells incubated with ST135 **5** compared to cells treated with β-methyl-GS-441524-DP **4** (Fig. 7a). For the latter, cellular uptake reached an equilibrium after 15 min, whereas for prodrug **5**, levels of β-methyl-GS-441524-DP **4** rapidly increased and still rose after 15 min. De-esterification of the AB-mask inside cells supports accumulation of the drug.

In contrast to cells incubated with β-methyl-GS-441524-DP **4**, we observed formation of GS-441524-MP **40** in cells incubated with 50 µM of prodrug **5**, reaching 24% of the total of detectable mono- **40** and β-methyl-diphosphate **4** (Fig. 7b, c), indicating a destabilizing effect of the AB mask on the pyrophosphate, consistent with reduced chemical stability, which initially was observed during the measurements of NMR spectra of the product ST135 **5** (Supplementary Fig. 12). When performing hydrolysis studies regarding the chemical stability of the prodrug **5** at pH7.3 a half-life of 61 h was determined, and an incipient decomposition to the GS-441524-MP **40** was discovered after only 30 s (Supplementary Fig. 13a). However, prodrug **5** hydrolysis with pig liver esterase (PLE) proved to be successful, as the unmasked diphosphate was primarily detected immediately after addition of the enzyme (Supplementary Fig. 13b).

To stabilise the anhydride bond, we replaced the β-methyl phosphate ester moiety in ST135 **5** by an ethyl phosphonate. We observed previously a marked increase in stability of a masked terminal phosphonate group attached to an unmasked phosphate forming a phosphonate-phosphate anhydride bond as compared to a masked terminal phosphate-phosphate anhydride linkage^53,54^. As a side reaction the cleavage of this anhydride bond was observed. The synthesis of β-ethyl-GS-441524-phosphate-phosphonate **42** (ST161) and the respective C_11_-AB-masked prodrug **6** (ST166) is shown in Fig. 6. This β-ethyl-phosphonate phosphate of GS-441524 **42** was less potent than β-methyl-GS-441524-DP **4** (pIC_50_ 5.81, Fig. 5b) due to a lower affinity (0.86 µM, Fig. 7d) but is still significantly more potent than ADPR **1**. The structure of Mac1 in complex with ST161 **42** (PDB 9RHN, Fig. 7e and Supplementary Fig. 11b) showed a binding mode similar to that displayed by β-methyl-GS-441524-DP **4** (PDB 9RHO, Supplementary Fig. 11a).

The uptake of ST166 **6**, the C_11_-AB-masked prodrug of ST161 **42**, in CaLu-3 cells was comparable to ST135 **5** (Fig. 7a). After 1 h similar cellular concentrations of the unmasked compounds were reached, whilst significantly less GS-441524-MP **40** was formed (11% of the total of detectable **40** and β-ethyl-phosphonate-phosphate) (Fig. 7a-c). Chemical hydrolysis studies showed no degradation to the monophosphate (Supplementary Fig. 14a). Additionally, during synthesis, purification and analysis, no decomposition was noticed and this compound did not require rapid and special handling and storage. Enzymatic hydrolysis of the C_11_-AB-masked phosphate-phosphonate ST166 **6** was very fast. After one minute, the prodrug **6** was converted to the active nucleotide **42** by PLE at 37 °C and physiological pH (Supplementary Fig. 14b), whilst there was only moderate decomposition in cell culture medium and PBS (t_1/2_ = 45.5 h and 71.5 h respectively, Supplementary Figs 15, 16). This confirms the higher chemical stability of the phosphate-phosphonate analogs, which reduced the tendency to cleave the masked phosphonate-phosphate bond.

Upon treatment with prodrugs ST135 **5** and ST166 **6**, the β-NAD^+^ **2** levels in CaLu-3 cells decreased within 60 min by 62% and 67%, respectively, which was not the case without inhibitor (Fig. 7c), potentially indicating stress exerted by the C_11_-AB-masked compounds. We thus investigated the impact of the prodrugs on cell viability in CaLu-3 cells (Fig. 7f). For comparison the membrane permeable^55^ parental nucleoside GS-441524 **3** was used. While ST135 **5** exhibited little cytotoxicity in the low micromolar range (CC_50_ > 50 µM), ST166 **6** showed no detectable cytotoxicity over the concentration range. Thus, both C_11_-AB masked prodrugs **5** & **6** are well tolerated by CaLu-3 cells.

### Antiviral activity in cell culture infection assays

**Fig. 8:**
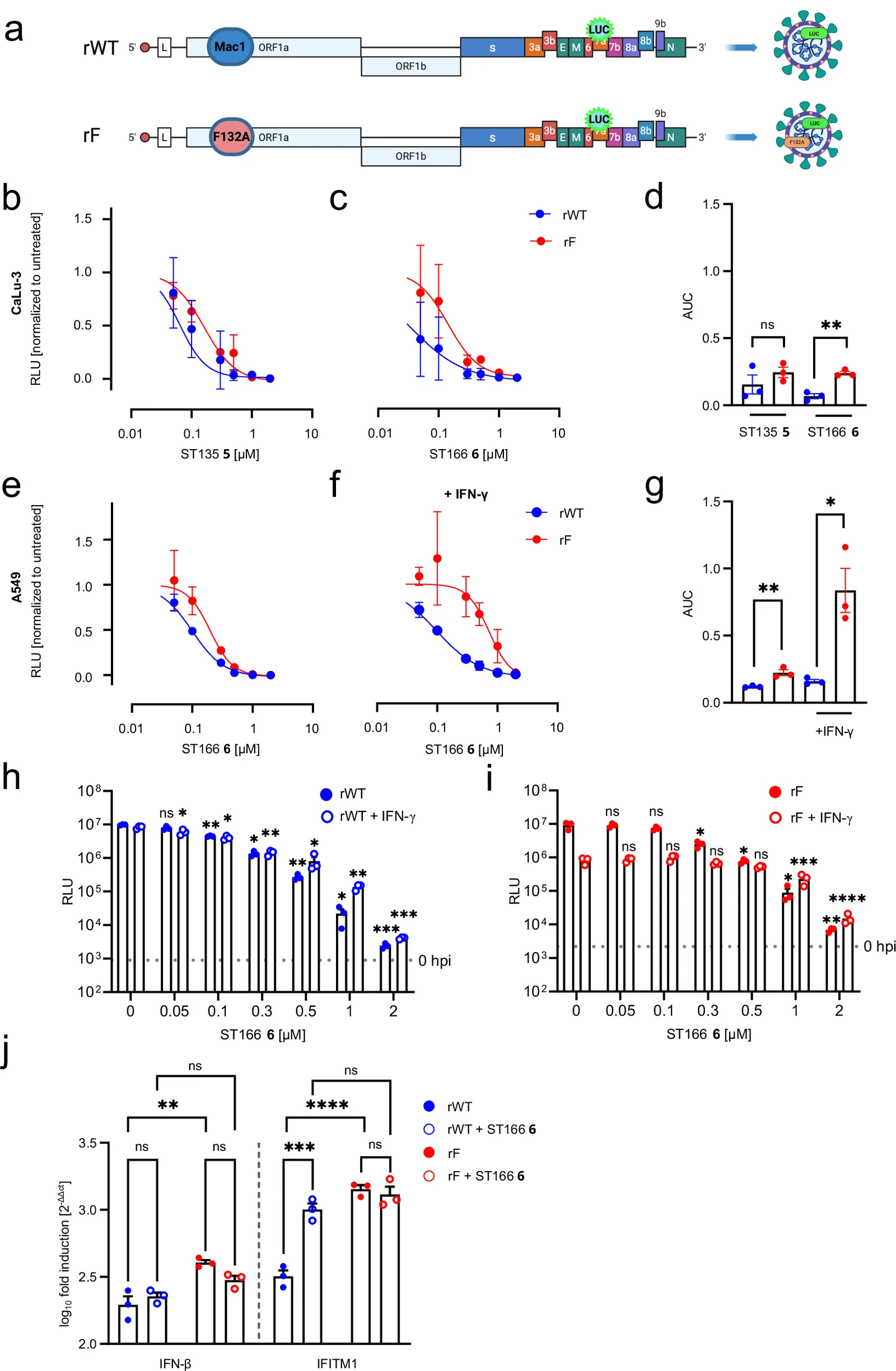
Prodrugs of β-methyl-GS-441524-DP and ST161 potently inhibit SARS-CoV-2 in cell culture. **a** Schematic overview of the recombinant viruses. Created in BioRender. Pfefferle, S (2026) https://BioRender.com/4gqy85y CaLu-3 cells were infected with rWT or rF at MOI = 1 and treated with **b** ST135 (C_11_-β-methyl-GS-441524-DP) **5** or **c** ST166 **6** at increasing concentrations. Luciferase reporter activity (RLU) was measured 24 hpi and (**d**) corresponding area under the curve (AUC) values from each experiment compared by two-sided unpaired t-tests. Adjusted *p*-values (rF vs rWT): **5**: 0.3209, **6**: 0.0017. A549-A/T cells were infected with rWT or rF at MOI = 1 and treated with **e** ST166 **6** or **f** additionally stimulated with IFN-γ (250 U) prior to infection. RLU and (g) corresponding AUC values were determined and statistically tested as above. Adjusted *p*-values (rF vs rWT): +IFN-γ: 0.0146, without IFN-γ: 0.0091. **h**, **i** Raw RLU data underlying (**e**–**g**) were tested by lognormal ANOVA with Šídák-Holm-corrected post hoc test against prodrug-untreated controls. A dashed line indicates the RLU at 0 hpi. Adjusted *p*-values: **h** rWT (+/- IFN-γ): 0.05 µM: 0.0354/ 0.0618, 0.1 µM: 0.0101/ 0.0033, 0.3 µM: 0.0047/ 0.0118, 0.5 µM: 0.0283/ 0.0052, 1 µM: 0.0064/ 0.0118, 2 µM: 0.0007/ 0.001; **i** rF (+/- IFN-γ): 0.05 µM: 0.7691/ 0.9429, 0.1 µM: 0.7691/ 0.359, 0.3 µM: 0.7691/ 0.0115, 0.5 µM: 0.2833/ 0.0157, 1 µM: 0.0003/ 0.0137, 2 µM: <0.0001/ 0.0058. **j** CaLu-3 cells were infected with rWT or rF at MOI = 1 with or without ST166 **6** (0.5 µM) treatment 16 hpi. Induction of IFNβ and IFITM1 (mRNAs) was calculated at 48 hpi relative to uninfected, untreated cells. Two-way ANOVA with Šídák–corrected post-hoc t-tests of log-transformed data were performed. Adjusted *p*-values (IFNβ/ IFITM1): rWT + **6** vs. rWT without **6**: 0.7438/ 0.0002, rF + **6** vs. rF without **6**: 0.1737/ 0.9601, rWT without **6** vs. rF without **6**: 0.0019/ < 0.0001, rWT + **6** vs. rF + **6**: 0.2305/ 0.3958. Data points (*n* = 3 independent infections) are represented, means ± SEM are given. ns: not significant, *p ≤ 0.05 **p ≤ 0.01,***p ≤ 0.001, ****p ≤ 0.0001. Source data are provided as a Source Data file.

To study the effect of inhibitors on SARS-CoV-2, we constructed recombinant SARS-CoV-2 encoding a luciferase reporter and either wild-type Mac1 (rWT) or catalytically inactive Mac1 rF132A (rF) to assess the contribution of Mac1 catalytic activity to the antiviral effects observed with our compounds. (Fig. 8a).

Next, in order to test if the Mac1 mutant binds the active metabolites (compound **4** and **42**) of our prodrugs (**5** and **6**) differently than the wildtype, we determined binding affinities. As expected, Phe132Ala Mac1 binds to both compounds **4** and **42**, albeit with somewhat higher affinities (0.03 and 0.54 µM respectively, Supplementary Fig. 17).

Both recombinant viruses replicated on CaLu-3 and A549 cells. Pre-stimulation of cells with IFN-γ (Supplementary Fig. 18) resulted in a pronounced decrease of transcriptional activity of rF compared to rWT with an almost complete loss of reporter activity of rF on CaLu-3 cells, in line with previous reports by Kerr and coworkers^56^.

Importantly, ST135 **5** inhibited both recombinant viruses at low concentrations on CaLu-3 cells (EC_50_ rWT 0.06 µM, EC_50_ rF 0.16 µM, Fig. 8b). In addition, rWT and rF were inhibited at comparably low concentrations of ST166 **6** (EC_50_ rWT 0.03 µM, EC_50_ rF 0.144 µM, Fig. 8c), yet a Mac1-specific effect was observed with rF tolerating significantly higher concentrations of ST166 **6** (Fig. 8c, d).

Full inhibition of replication of rWT and rF by both prodrugs **5** and **6** indicates that the active metabolites (β-methyl-GS-441524-DP **4** and ST161 **42**) have an additional mode of action. Due to the structural relationship to the nucleoside core of remdesivir, we suspected that the viral RNA-dependent RNA polymerase (RdRp) could be an additional target of Mac1 inhibitors. We thus assessed if compounds **4** and **42** are substrates and/or inhibitors of the minimal replication/transcription complex (RTC) of SARS CoV-2 in vitro. (Supplementary Fig. 19) Primer elongation analysis shows that SARS CoV-2 RTC weakly incorporated both compounds **4** and **42**. However, when the compounds **4** and **42** were part of a NTP mixture, we observed neither incorporation of the compounds nor inhibitory effects (up to 250 µM) in contrast to the Remdesivir-metabolite GS-41524-TP **43**. This suggested that the Mac1 inhibitors did not directly affect RTC function.

The catalytic activity of Mac1 is particularly important for the virus in activated cells^56^. We therefore determined the effect of prodrug **6** on viruses in IFN-γ treated cells, switching to A549-A/T cells that in contrast to CaLu-3 cells, allowed sufficient viral growth in the IFN-γ induced active state (Supplementary Fig. 18).

In untreated A549-A/T cells, both viruses were inhibited at low concentrations of ST166 **6** (EC_50_ WT 0.09 µM), with rF132A (rF) being somewhat less sensitive (EC_50_ 0.19 µM, Fig. 8e). Of note, upon IFN-γ stimulation of A549-A/T cells, rF (with inactive Mac1) was even less sensitive to prodrug **6** (EC_50_ rWT 0.09 µM, EC_50_ rF 0.72 µM, Fig. 8f, g). This is also reflected by the raw luciferase data showing that the low level replication of rF in the presence of IFN-γ only responds to ST166 **6** at 1 µM and above (Fig. 8h, i). The dynamic range of the luciferase assay is mostly limited by the residual luciferase present in the input viral particles (indicated in Fig. 8h, i and Supplementary Fig. 18 as dashed line denoting 0 hpi) and covers approx. 3.5 log steps. This was confirmed by using 2 µM of the RdRp inhibitor remdesivir to fully inhibit viral replication (Supplementary Fig. 20).

As the luciferase activity does not necessarily reflect the amount of infectious particles. Therefore, we additionally performed a cell-based titration assay with A549-A/T cells in the absence of IFN-γ using a lower MOI of 0.01. Quantification of the infectious viral titres confirmed near-complete suppression of rWT at 0.3 µM ST166 **6**, which had no significant effect on rF (Supplementary Fig. 21).

### Determining cellular effects upon ST166 treatment

Since we observed a drop in NAD^+^ upon prodrug treatment in our uptake assays (Fig.7c), we assessed the effect of ST166 **6** treatment on cellular NAD^+^ by quantifying total NAD-levels in CaLu-3 cells (Supplementary Fig. 22). In uninfected cells, IFN-γ treatment caused a roughly 40% reduction in NAD-pools. SARS-CoV-2 infection had no significant effect on cellular NAD-levels at 2 hpi and caused an approximately 30% reduction at 48 hpi. ST166 **6** alone had no significant effect on cellular NAD levels, which did not further alter in combination with viral infection or IFN-γ preincubation.

Next, we analyzed the induction of IFN-β and IFITM1 as representative ISGs in cells infected with a MOI of 1 of either rWT or rF. To allow infection to become established prior to inhibitor treatment ST166 **6** (0.5 µM) was added 16 hpi (Fig. 8j). Viral RNA levels proved comparable under all conditions (Supplementary Fig. 23). Both viruses induced IFN-β expression; however, transcript levels were significantly higher in rF-infected cells than in rWT-infected cells (404-fold vs. 200-fold). Treatment with ST166 **6** increased IFN-β induction in rWT-infected cells to levels comparable to those observed for rF (299-fold vs. 227-fold).

Induction of IFITM1 showed an even more pronounced difference between the viruses, with rF triggering substantially higher expression than rWT (1435-fold vs. 322-fold). Inhibition of Mac1 by ST166 **6** markedly enhanced IFITM1 induction in rWT-infected cells (1014-fold), elevating expression to levels similar to those induced by rF (1326-fold). In contrast, ST166 **6** had no significant effect on IFITM1 induction in rF-infected cells. Consistent trends were observed under conditions of IFN-γ pretreatment (Supplementary Fig. 24). Here, ST166 **6** was added at 2 hpi at 0.1 µM, which may affect direct comparability with the experimental setup described above. In this setting, ST166 **6** significantly increased IFITM1 induction in rWT-infected cells, whereas ISG induction in rF-infected cells remained unaffected.

## Discussion

While nucleoside and nucleotide derivatives have been used to develop antiviral drugs, efforts to develop Mac1 inhibitors focused mostly on identifying non-nucleotide small molecules. Notable achievements have been made by other groups, which yielded Mac1 inhibitors that also showed effect in viral infection assays^18–20^. For instance, following a biochemical HTS of 30,400 compounds, Wazir et al. identified a 2-amide-3-methylester thiophene scaffold that led to the synthesis of MDOLL-0229 with an IC_50_ of 2.1 µM and a reported EC_50_ between 12.5 and 25 µM in SARS-CoV-2 infected CaLu-3 cells^18^. In addition, Pfannenstiel et al. conducted in silico-assisted structure-based optimization of a tryptophanate precursor MCD-628^57^ and generated two membrane-permeable antivirals (compound 5c and 6e) with an IC_50_ of 4.9 and 3.7 µM respectively, which both inhibited SARS-CoV-2 replication in CaLu-3 cells at 12.5 µM^19^. Furthermore, capitalizing on previously described Mac1 inhibitor candidates^16^, Suryawanshi et al. performed an extensive medicinal chemistry campaign to merge key structural elements of AVI-92 and AVI-219, which yielded the AVI-4206, a pyrrolidinone with additional urea-functionality exhibiting an IC_50_ of 64 nM while impairing SARS-CoV-2 replication in human airway organoids at 4 µM^20^.

We, in contrast, used an assorted, purine-targeted library that includes many ADP(R) analogs not commercially available and probed the relative importance of the different structural elements of ADPR **1**, which resulted in an initial complete SAR and allowed us to come up with a nucleotide-based inhibitor of Mac1. We found that replacement of N7 by a carbon atom (CH) increases affinity and inhibitory potency consistently across all tested compound pairs (Fig. 4). This modification in adenine nucleotides increases the basicity of N1 thus enhances hydrogen bonding^25^, which illustrates the essential role of the hydrogen bond between the peptide bond N-H of Ile23 and N1 of the adenine base for the binding of ADPR/ADP analogs to Mac1 and the potential to enhance these interactions via manipulation of the pK_a_ of the base (Fig. 4). In a recent study derivatives of 7*H*-pyrrolo(2,3-*d*)pyrimidine, the the base of 7-deaza-ADPR were found to bind in the active site of Mac1, although these compounds assumed a variety of binding poses^13^, making this moiety a promising scaffold for Mac1 inhibitors^57^.

Most ADP derivatives showed poor activity consistent with previous findings that Mac1 is not thermally stabilised by ADP **18**^58^, likely due to the negatively charged β-phosphate causing local electrostatic repulsion within the predominantly hydrophobic environment (Ile131 and Phe132). Nucleoside diphosphates with a substituent at the β-phosphate do not show this electrostatic repulsion. Upon ADPR **1** binding, structured water molecules in the binding site of the distal ribose are reorganised or displaced to accommodate the ligand^59^. The hydroxyl groups of the ribofuranose could partially replace the interactions with the water molecules, preventing the loss of enthalpy when they are displaced by a large, mostly aliphatic substituent such as the tetrahydrofurfuryl or the cyclopentyl group. Apparently, the entropic gain due to desolvation, or small enthalpic gains due to additional van der Waals interactions, cannot counteract the enthalpy loss due to the displaced water. In contrast to larger substituents, the small alkyl chains keep the water molecules in place while preventing the enthalpy loss due to electrostatic repulsion, consistent with the large differences in binding enthalpy between β-methyl-GS-441524-DP **4** and GS-441524-DP **41**. The reduced inhibitory potency and affinity of the β-ethylated nucleoside-diphosphates compared to the β-methylated nucleoside-diphosphates could have two contributions: the longer alkyl chain imposing additional constrains on the solvation network, thus being entropically unfavourable, or the +I effect of the alkyl chain reducing the acidity thus the hydrogen bonding capacity of the β-phosphate^60^ thereby negatively affecting the net binding enthalpy. This would be in line with the reduced inhibitory potency and affinity of the ethyl phosphonate ST161 **42** as phosphonates have a higher second pK_a_ than phosphates^61^. This suggests that the methyl group is in the Goldilocks zone, providing protection from the electrostatic repulsion while keeping solvation of the binding pocket mostly undisturbed (Fig. 4).

β-Methyl-GS-441524-DP **4** exhibited no detectable inhibition of the human macrodomains MacroD1 and MacroD2 under the conditions tested, suggesting an initial degree of selectivity for Mac1. The reason for the high selectivity of the GS-441524 derivative **4** probably lies in the GS-441524 **3** nucleoside component. Ni et al. demonstrated that the 1′-cyano group of GS-441524 **3** inserts into a pocket near loop β7-α6, interacting with the backbone N-H groups of Phe156 and Asp157, which provides a positive dipole to stabilise the negative dipole of the cyano group^21^. Schuller et al. aptly referred to this region as the oxyanion hole as they identified several anionic fragments binding within this site^13^. In human MacroD1/MacroD2, a leucine in the oxyanion hole is replaced by aspartate. The electrostatic incompatibility of the aspartate with the partial negative charge of the cyano group might contribute to the specificity of the β-alkylated GS-441524 DP **4,** **42** for Mac1.

However, the present selectivity analysis remains limited, as only two human macrodomains were examined. Given the nucleoside analog nature of these compounds, broader profiling against additional macrodomains and other host ADP-ribose/NAD-binding proteins will be important to more comprehensively assess selectivity and potential off-target effects in future studies. While GS-441524 **3** enters cells by adenosine transporters^62^, we found that β-methyl-GS-441524-DP **4** was only detectable in lysates of cells treated with very high concentrations, likely reflecting binding to cell surface receptors^62^. The charged diphosphate in the inhibitors thus required us to turn them into prodrugs by linking a C_11_-AB group to the phosph(on)ate. As described for other masked nucleoside phosphates, deprotection of the prodrugs sustains the concentration gradient enabling efficient uptake of the active compound by passive diffusion^35^ to intracellular levels that exceed treatment concentration ( > 3-fold for both ST135 **5** and ST166 6)^63^. The prodrugs accumulated in CaLu-3 cells even at low concentrations that showed no cytotoxicity. In contrast to other studies with Mac1 inhibitors^11^, we observed a clear inhibitory effect of our prodrugs in cell culture infection experiments.

Recombinant virus with intact Mac1 (rWT) tended to be inhibited by lower concentrations than virus with catalytically inactive Mac1 (rF), which was particularly evident in IFN-γ pre-stimulated cells, underlining the importance of Mac1 in interacting with the host^39,56,64^. Upon treatment with IFN-γ, which sensitises cells for Mac1 inhibition^20^, the rF was more tolerant to the inhibitor. This is in contrast to MDOLL-0229 as well as of compounds 5c and 6e described previously^18,19^, which exhibited antiviral effects only in the presence of IFN-γ. The recently described AVI-4206 was able to impair SARS-CoV-2 replication at 4 µM in an organoid system even without exogenous IFN-γ^20^, consistent with our observation.

Notably, rF was not completely resistant, suggesting the presence of a secondary, Mac1-independent mode of action of prodrugs **5** and **6**. Given the remdesivir-derived core motif present in both compounds, a plausible additional viral target is the viral RNA-dependent RNA polymerase (RdRp). Although the active metabolites **4** and **42** do not substantially interfere with replication-transcription complex (RTC) function under physiological conditions, partial destabilization of the pyrophosphate moiety by the masking group in prodrug **5** could promote formation of GS-441524-MP **40** upon intracellular processing. Subsequent phosphorylation to the corresponding triphosphate **43** could, in principle, inhibit the viral RdRp^65^, analogous to the mechanism of remdesivir. Consistent with this interpretation, stabilization of the pyrophosphate in compound **6** increased the differential inhibition observed between rWT and rF inhibition, indicating a shift toward preferential Mac1 targeting. While the contribution of polymerase inhibition remains to be formally established, a dual mode of action maybe advantageous from a therapeutic perspective, as resistance development would likely require concurrent adapation in two independent viral targets.

Although rF led to a significantly stronger IFN-β and IFITM1 induction than rWT, for induction of IFN-β no statistically significant differences between viral variants were observed in IFN-γ stimulated cells irrespective of the presence of inhibitor **6**. In contrast, the ISG IFITM1 was not only induced by both viruses but also revealed significant differences between the viral variants. Notably, IFITM1 expression was enhanced in the presence of ST166 **6** by rWT but not by rF, indicating that the catalytic activity of Mac1 plays a critical role in suppressing the expression of IFITM1, suggesting that while both viruses can initiate interferon production, only the wild-type variant actively dampens downstream expression of ISGs via Mac1.

Here we demonstrate an extensive biochemical, chemical and structural investigation of Mac1 of SARS-CoV-2, enabling a SAR-guided development of two potent membrane permeable prodrugs **5** and **6** for effective inhibition of SARS-CoV-2 in cells. Besides the unconventional nucleotide-based compound design combined with the innovative prodrug-approach, this study contributes tools for the investigation of ADP-ribosylation in host-virus interaction and antiviral candidates for further (pre-) clinical testing. Due to the high structural conservation of viral macrodomains in all CoVs, alphaviruses and orthohepeviruses, the significance of these findings is not limited to SARS-CoV-2.

## Methods

### Ethics

The genetic engineering work was approved by the authorities (Hamburg Ministry of the Environment, Climate, Energy and Agriculture, BUKEA) (AZ I14-29/2022).

### Chemicals and compound handling

8-Br-ADPR **13**, 8-Br-7-deaza-ADPR **14**, IDPR **10**, 1*N*^6^-etheno-ADPR **9**, ADPR-2′-phosphate **15**, 2′-deoxy-ADPR **16**, 2′-deoxy-2′-F-ADPR **17**, 2′-deoxy-ADP **19**, 7-deaza-ADP **20**, 7-deaza-2′-deoxy-ADP **21**, adenosine-5′-*O*-(β-thiodiphosphate) 35, (*R*p)- **36** and (*S*p)-isomer 37 of adenosine-5′-*O*-(α-thiodiphosphate), and CH_2_-ADP **34** were purchased from Biolog (Bremen, Germany). ADPR **1**, adenosine **38**, AMP **39**, ADP **18**, α-NAD^+^ **7**, β-NAD^+^ **2**, β-NMN **44**, nicotinamide **8** were bought at the highest available purity from Sigma Aldrich. GS-441524 **3** was sourced from MedChem Express. NAMPT inhibitor (FK866) was purchased from Sigma-Aldrich. Most reactions were carried out under anhydrous conditions and nitrogen atmosphere. All anhydrous solvents were purchased from Acros (extra dry over molecular sieves). All starting materials and reagents were purchased from Sigma-Aldrich, TCI, Acros, ABCR or Carbosynth and were used without further purification. Ultrapure water was obtained from a Sartorius arium pro system (Sartopore 0.2 μm, UV). HPLC grade CH_3_CN used for automated RP-18 flash chromatography and HPLC analysis was purchased from VWR or Honeywell. Column chromatography was performed with technical grade solvents, which were distilled prior to use. Silica gel 60 M (0.04–0.063 mm) and thin-layer-sheets (ALUGRAM^®^ Xtra SIL G/UV254) used for thin-layer chromatography were purchased from Macherey-Nagel. Visualisation of non-UV active compounds was done by heat-staining with vanillin in methanol, acetic acid and sulfuric acid. Automated RP-18 flash chromatography was carried out using prepacked MN RS 40 C18ec columns on an Interchim Puriflash 430. NMR solvents were purchased from Euroiso-Top or Deutero. NMR spectra were recorded on Bruker instruments Fourier HD 300, Avance I 400, Avance I 500, and Avance III HD 600 at room temperature. All chemical shifts (δ) were given in ppm and calibrated on solvent signals. High-resolution mass spectra from samples dissolved in water or acetonitrile (LC-MS grade) were measured with an Agilent 6224 ESI-TOF instrument (10 µg/mL, injection volume 2.5 µL) using electron-spray ionization (ESI) time of flight mass spectrometry in negative mode (mass range of m/z 110-3200 and a rate of 1.03 spectra/s) at a gas temperature of 325 °C and a drying gas flow to 10 L/min. The synthesis of 2-F-ADPR **11**, 8-(thiophen-3-yl)-ADPR **12**, β-cyclopentyl-ADP **27**, and 5-ribosyl-squaryl-adenosine 30 has been described previously^66^. The synthesis of THF-ADP **26** and β-methyl-ADP **29** has been described previously^67^. The synthesis of 2′,3″-dideoxy-ADPR **24** and 1″,2′-dideoxy-ADPR **25** and has been described previously^68^. The synthesis of adenosine-5′-*O*-(2-phosphoryl)acetate ribose (A-acetyl-PR) **31**, adenosine-5′-phosphonoacetyl ribose (AMP-acetyl-R) **32** has been described previously^43^. CH_2_-ADPR **33** was a kind gift from László Csanády (Semmelweiß University, Budapest).

All compounds were dissolved in ultrapure water (Riedel-de Haen), with the exception of 5-ribosyl-squaryl-adenosine **30**, AMP-acetyl-R **32**, A-acetyl-PR 31, GS-441524 **3**, C_11_-AB-β-methyl-GS-441524-DP (ST135) **5**, C_11_-AB-β-ethyl-GS-441524-phosphate-phosphonate (ST166) **6** and FK866, which were dissolved in DMSO. All compounds were frozen in aliquots, stored at -80 °C and checked for integrity before use by HPLC. Compounds were routinely >95% pure by HPLC.

### Enzymes and other reagents

Human MacroD1 and MacroD2 were a kind gift from Andreas Ladurner (LMU, Munich), PLE and NADase from *Neurospora crassa* were sourced from Sigma Aldrich.

### HPLC analysis

Samples were thawed on ice and passed through a 10 kDa size exclusion filter (Pall) prior to analysis by reverse-phase HPLC system (1260 series, Agilent Technologies) equipped with a 250 mm × 4.6 mm C8 Luna column (5 μm particle size, Phenomenex) as stationary phase and a flow rate of 0.8 ml/ min.

Usually, the mobile phase consisted of HPLC buffer A (20 mM KH_2_PO_4_, pH 6.0) and B (50 % A, 50 % MeOH) and the percentage of buffer B was gradually increased over time (0 min 0 % buffer B, 5 min 0 % buffer B, 27.5 min 100 % buffer B, 30 min 100 % buffer B, 32 min 0 % buffer B, 43 min 0 % buffer B).

For analysis of cells treated with masked **5** and unmasked β-methyl-GS-441524-DP **4**, the mobile phase consisted of HPLC buffer A and C (10% A and 90 % MeOH) and the percentage of buffer C was gradually increased over time (0 min 0 % buffer C, 5 min 0 % buffer C, 27.5 min 55 % buffer C, 30 min 55 % buffer C, 35 min 100 % buffer C, 45 min 100 % buffer C, 47 min 0 % buffer C, 57 min 0 % buffer C).

For analysis of cells treated with phosphate-phosphonate derivatives of masked **6** and unmasked ST161 **42**, the mobile phase consisted of HPLC buffer D (A, supplemented with 5 mM tetrabutylammonium dihydrogen phosphate, Sigma Aldrich) and E (10 % D and 90 % MeOH) and the percentage of buffer E was gradually increased over time (0 min 0 % buffer E, 5 min 0 % buffer E, 27.5 min 55 % buffer E, 35 min 100 % buffer E, 45 min 100 % buffer E, 47 min 0 % buffer E, 57 min 0 % buffer E).

Light absorption by the analytes was recorded by a diode array detector (DAD, Agilent Technologies) usually at 260 nm with exception of GS-441524 derivatives, which were measured at 245 nm. For quantification, peak area was calibrated using external standards.

Hydrolysis studies of the prodrugs were carried out in PBS buffer (Dulbecco’s Phosphate Buffered Saline, Gibco) or cell medium (RPMI, Gibco/ Life Technologies) at 37 °C. The incubation solution was prepared with a final volume of 725 μL (0.917 mM) by adding 13.3 μL of the stock solution (50 mM stock solution of the respective prodrug in DMSO) to 363 μL PBS buffer or cell medium, 228 μL water, and 121 μL DMSO. The incubation was performed without internal standard in a thermomixer (TS basic, CellMedia). At indicated times, aliquots of 20 μL (prodrug **5**) or 35 µL (prodrug **6**) were taken and analyzed by HPLC with an injection volume of 15 μL (prodrug **5**) or 30 µl (prodrug **6**). Enzymatic hydrolysis was carried out by preparing an incubation solution with a final volume of 1.00 mL (0.453 mM). 9.10 μL of the stock solution (50 mM of the respective prodrug in DMSO) was added to 56.6 μL PLE (100 units/mg), 755 μL PBS buffer, and 180 μL DMSO. The incubation was performed without internal standard in a thermomixer at 37 °C, pH 7.3. At indicated times, aliquots of 60 μL were taken and 120 μL cold MeOH was added (protein precipitation MeOH/aqueous 2:1), and vortexed (vortexer REAX 2000, Heidolph). The aliquots were placed on ice for 5 minutes and then centrifugated for 10 minutes at +4 °C. The supernatant was filtered using a syringe filter and aliquots of 30 μL were taken and analyzed by HPLC with an injection volume of 20 μL. The analytical HPLC used was an Agilent Technologies system of the model 1260 Infinity II. The stationary phase was a reverse phase column EC 125/3 Nucleodur 100-5 C_18_ ec from Macherey-Nagel. The mobile phase consisted of a 2 mM Tetra-*n*-butylammonium acetate buffer (pH 6) and CH_3_CN (HPLC grade). The percentage of CH_3_CN was gradually increased over time (0 min, 95 % buffer, 5 % CH_3_CN; 20 min, 20 % buffer, 80 % CH_3_CN) and then kept isocratic for 10 minutes (30 min, 20 % buffer, 80 % CH_3_CN). The percentage of CH_3_CN was again decreased gradually (33 min, 95 % buffer, 5 % CH_3_CN) and kept isocratic for 1 minute (34 min, 95 % buffer, 5 % CH_3_CN). The flowrate was 1.0 mL/min and UV detection by a DAD (Agilent Technologies) was measured at 240 and 250 nm or 270 nm for chemical hydrolysis studies of prodrug **6**.

Chemical synthesis of β-ethyl-ADP 28, GS-441524-MP **40**, GS-441524-DP **41**, β-methyl-GS-441524-DP **4**, C_11_-AB-masked β-methyl-GS-441524-DP (ST135) **5**, β-ethyl-GS-441524-phosphate-phosphonate (ST161) **42**, C_11_-AB-masked β-ethyl-GS-441524-phosphate-phosphonate (ST166) **6**, 7-deaza-ADPR **22**

#### General procedure 1: phosphorylation via DCI-mediated coupling of a phosphoramidite

The reaction was performed under nitrogen atmosphere and dry conditions. 1.0 eq. of the respective alcohol was dissolved in CH_2_Cl_2_ and 1.5 eq. phosphoramidite were added. Subsequently, 2.0 eq. 4,5-dicyanoimidazole (0.25 mol/L in CH_3_CN, DCI) were added dropwise and the mixture was stirred for 3 h at room temperature. Oxidation was performed at 0 °C by portionwise addition of 1.5 eq *tert*-butyl hydroperoxide (5.5 M in *n*-decane). After stirring for 1 h at 0 °C, the reaction mixture was washed once with saturated NaHCO_3_ solution. The aqueous phase was extracted with CH_2_Cl_2_ three times. The combined organic layers were dried over Na_2_SO_4_ and the solvent was removed in vacuo. The crude product was purified by silica gel chromatography.

#### General procedure 2: imidazolidate-mediated formation of a phosphodiester bond

The reaction was performed under nitrogen atmosphere and dry conditions. GS-441524-MP **40** was suspended in CH_3_CN (1 mL/10 mg) and cooled to 0 °C. To the suspension, a cooled mixture containing 16 eq. Et_3_N and 10 eq. TFAA dissolved in CH_3_CN was added dropwise. It was stirred for 10 min at 0 °C and then all volatiles were removed in vacuo. The residue was redissolved in CH_3_CN and 10 eq. Et_3_N and 6.0 Eq. 1-methylimidazole (NMI) were added at room temperature. The reaction mixture was stirred for another 10 min. Subsequently, the second phosphate dissolved in CH_3_CN or DMF (1 mL/10 mg) was added dropwise. After full consumption of the starting material all volatiles were removed in vacuo and the crude product was purified by automatic RP18 flash chromatography. This was followed by conversion to the ammonium and triethylammonium forms using a cation-exchange resin (Dowex 50WX8), with subsequent automated RP18 flash chromatography (H_2_O/CH_3_CN 0-100 % Vol., 0-50 min; flow rate 20 mL/min), respectively. Product-containing fractions were freeze-dried.

#### GS-441524-MP 40

The reaction was performed according to Ni and coworkers^21^. The product was obtained as a colourless solid (95 %). ^**1**^**H-NMR** (400 MHz, D_2_O): *δ* [ppm] = 8.09 (s, 1H, H-2), 7.39 (d, ^3^*J*_H,H_ = 4.9 Hz, 1H, H-7), 7.16 (d, ^3^*J*_H,H_ = 4.8 Hz, 1H, H-8), 4.84 (d, ^3^*J*_H,H_ = 5.2 Hz, 1H, H-2‘), 4.52-4.48 (m, 1H, H-4‘), 4.48 (t, ^3^*J*_H,H_ = 4.9 Hz, H-3‘), 4.10-4.04 (m, 2H, H-5‘). – ^**13**^**C-NMR** (101 MHz, D_2_O): *δ* [ppm] = 148.7 (C-6), 135.5 (C-2), 128.4 (C-9), 118.3 (CN), 116.6 (C-5), 113.3 (C-8), 109.9 (C-7), 84.6 (C-4′), 75.3 (C-2′), 70.2 (C-3), 63.9 (C-5′). – ^**31**^**P-NMR** (202 MHz, D_2_O): *δ* [ppm] = 1.31. – **HRMS** (ESI^-^, m/z): calcd.: 370.0558 [M-H]^-^, found: 370.0549 [M-H]^-^.

#### GS-441524-DP 41

GS-441524-MP **40** (60 mg, 0.16 mmol) was suspended in CH_3_CN (3 mL), before Et_3_N (269 µL, 1.94 mmol) and TFAA (224 µL, 1.62 mmol) were added dropwise. After the reaction mixture was stirred for 10 min at room temperature, all volatiles were removed under reduced pressure, and the residue was dissolved again in CH_3_CN (3 mL). The solution was then treated with Et_3_N (224 µL, 1.62 mmol) and 1-methylimidazole (77 µL, 0.97 mmol). After the reaction mixture was stirred for another 10 min at room temperature, it was added dropwise via a syringe pump (0.2 mL/min) to a solution of monobasic tetra-*n*-butylammonium phosphate (1.21 µL, 0.97 mmol, 0.4 M solution in CH_3_CN) in CH_3_CN (2 mL). Subsequently, the reaction mixture was stirred for 30 min at room temperature, before all volatiles were removed under reduced pressure; the residue was taken up in water and washed three times with CH_2_Cl_2_. The aqueous phase was then concentrated under reduced pressure and the residue was purified using automated column chromatographic purification using a DEAE-Sephadex® A-25 column (H_2_O/1 M TEAB; 100:0 to 0:100 v/v) and RP_18_ flash column chromatography (H_2_O/CH_3_CN; 100:0 to 0:100 v/v) provided the triethylammonium salt of GS-441524-DP **41** (64 mg) as a colourless solid (89%). ^**1**^**H-NMR** (400 MHz, D_2_O): *δ* [ppm] = 7.96 (s, 1H, H-8), 7.06 (d, ^3^J_HH_ = 4.75 Hz, 1H, *H*-5), 6.95 (d, 1H, ^3^J_HH_ = 4.80 Hz, *H*-6), 4.99 (d, ^3^J_HH_ = 5.05 Hz, 1H, *H*-2´), 4.55 – 4.52 (m, 2H, *H*-4´, *H*-3´), 4.18 (ddd, ^2^J_HH_ = 11.91 Hz, ^3^J_HP_ = 6.48 Hz, ^3^J_HH_ = 3.01 Hz, 1H, *H*-5´), 4.08 (ddd, ^2^J_HH_ = 11.78 Hz, ^3^J_HP_ = 5.15 Hz, ^3^J_HH_ = 2.79 Hz, 1H, *H*-5´), 3.20 (q, ^3^J_HH_ = 8.08 Hz, 18H, *H*-b), 1.28 (t, ^3^J_HH_ = 7.25 Hz, 12H, *H*-a). – ^**31**^**P-NMR** (202 MHz, D_2_O): *δ* [ppm] = -9.47 (d, ^2^J_PP_ = 21.93 Hz, *P*-β), -11.27 (d, ^2^J_PP_ = 20.99 Hz, *P*-α).

#### Dibenzylmethyl phosphate

The reaction was carried out according to general procedure 1, with 0.090 mL (2.2 mmol, 1.5 eq.) methanol, 0.49 mL (1.4 mmol, 1.0 eq.) dibenzyl-*N,N*-di*iso*propyl phosphoramidite and 28 mL CH_2_Cl_2_. 11 mL 4,5-Dicyanoimidazole (0.25 mol/L in acetonitrile) was added dropwise and the reaction mixture was stirred for 3 h at room temperature. Subsequently, the reaction mixture was cooled to 0 °C and 0.21 mL (2.2 mmol, 1.5 eq.) *tert*-butyl hydroperoxide was added dropwise. The reaction was stirred for another 1 hour and then washed once with a saturated NaHCO_3_. The aqueous phase was extracted with CH_2_Cl_2_ three times. The combined organic layers were dried over Na_2_SO_4_ and the solvent was removed in vacuo. The crude product was purified by silica gel chromatography (PE/EtOAc 2:1) to afford 0.31 g (1.1 mmol) of the product as a colourless oil (74 %). ^**1**^**H-NMR** (500 MHz, CDCl_3_): *δ* [ppm] = 7.39-7.30 (m, 10H, H-Aryl), 5.09-5.00 (m, 4H, H-1), 3.70 (d, ^3^*J*_H,P_ = 11.2 Hz, 3H, H-6). – ^**13**^**C-NMR** (126 MHz, CDCl_3_): *δ* [ppm] = 136.0 (d, ^3^*J*_C,P_ = 6.8 Hz, C-2), 128.7, 128.7, 128.1 (C-Aryl), 69.4 (d, ^2^*J*_C,P_ = 5.6 Hz, C-1), 54.4 (d, ^2^*J*_C,P_ = 6.0 Hz, C-6). – ^**31**^**P-NMR** (202 MHz, CDCl_3_): *δ* [ppm] = 1.51. – **HRMS** (ESI^+^, m/z): calcd.: 315.0757 [M+Na]^+^, found: 315.0763 [M+Na]^+^.

#### Methyl phosphate as triethylammonium salt

The reaction was carried out according to general procedure 2, with 0.15 g (0.51 mmol, 1.0 eq.) dibenzylmethyl phosphate, 11.25 mL MeOH, 1.125 mL water (alcohol/water 10:1) and 0.14 mL (1.0 mmol, 2.0 eq.) triethylamine and 30 mg (20 wt.-%) palladium hydroxide on activated charcoal. After vigorously stirring for 20 h at room temperature, the reaction was filtered over Celite^®^ using MeOH to afford 96 mg (0.51 mmol) of the product as a colourless oil in a quantitative yield. ^**1**^**H-NMR** (600 MHz, MeOH-*d*_4_): *δ* [ppm] = 3.55 (d, ^3^*J*_H,P_ = 10.9 Hz, 3H, CH_3_). – ^**13**^**C-NMR** (126 MHz, MeOH-*d*_4_): *δ* [ppm] = 53.10 (CH_3_). – ^**31**^**P-NMR** (243 MHz, MeOH-*d*_4_): *δ* [ppm] = 1.66. – **HRMS** (ESI^-^, m/z): calcd.: 110.9853 [M-H]^-^, found: 110.9856 [M-H]^-^.

#### β-methyl-GS-441524-DP 4

The reaction was carried out according to general procedure 1, with 7.9 mg (0.014 mmol, 1.0 eq.) GS-441524-MP **40**, 1 mL CH_3_CN, 0.030 mL (0.23 mmol, 16 eq.) triethylamine and 0.020 mL (0.14 mmol, 10 eq.) TFAA, dissolved in 1 mL CH_3_CN. The residue was redissolved in 2 mL CH_3_CN and 0.020 mL (0.14 mmol, 10 eq.) triethylamine, 0.010 mL (0.085 mmol, 6.0 eq.) NMI were added. After a stirring time of 10 min, 4.5 mg (0.021 mmol, 1.5 eq.) of methyl phosphate, suspended in 1 mL CH_3_CN, was added dropwise. The reaction mixture was stirred for 7 h at room temperature and then purified by automated RP18 flash chromatography (H_2_O/CH_3_CN 0–100 % Vol., 0-50 min; flow rate 20 mL/min). The product was obtained as a colourless solid (15 mg, 0.018 mmol, 85 %) as its triethylammonium salt. ^**1**^**H-NMR** (400 MHz, D_2_O): *δ* [ppm] = 8.11 (s, 1H, H-2), 7.41 (d, ^3^*J*_H,H_ = 4.9 Hz, 1H, H-7), 7.20 (d, ^3^*J*_H,H_ = 4.9 Hz, 1H, H-8), 4.98 (d, ^3^*J*_H,H_ = 5.1 Hz, 1H, H-2′), 4.58-4.52 (m, 1H, H-4′), 4.49 (t, ^3^*J*_H,H_ = 4.8 Hz, 1H, H-3′), 4.22-4.15 (m, 2H, H-5′), 3.61 (d, ^3^*J*_H,P_ = 11.4 Hz, 3H, -CH_3_). – ^**13**^**C-NMR** (101 MHz, D_2_O): *δ* [ppm] = 149.4 (C-6), 136.3 (C-2), 129.2 (C-9), 118.5 (CN), 117.1 (C-1′), 115.6 (C-5), 114.1 (C-8), 110.7 (C-7), 85.5 (d, ^3^*J*_C,P_ = 8.6 Hz, C-4′), 76.0 (C-2′), 70.9 (C-3′), 65.2 (d, ^2^*J*_C,P_ = 5.2 Hz, C-5′), 53.9 (dd, ^3^*J*_C,P_ = 9.1 Hz, ^2^*J*_C,P_ = 6.0 Hz, -CH_3_). ^**31**^**P-NMR** (162 MHz, D_2_O): *δ* [ppm] = -9.76 (d, ^2^*J*_P,P_ = 21.6 Hz, P_α_), -11.55 (d, ^2^*J*_P,P_ = 21.3 Hz, P_β_). – **HRMS** (ESI^-^, m/z): calcd.: 464.0378 [M-H]^-^, found: 464.0376 [M-H]^-^.

#### 4-(Hydroxymethyl)phenyldodecanoate 45

The reaction was performed according to Weising and coworkers^69^. The product was obtained as a colourless solid in a yield of 61 %. ^**1**^**H-NMR** (600 MHz, CDCl_3_): *δ* [ppm]7.41 – 7.35 (m, 2H, H-3), 7.10 – 7.05 (m, 2H, H-2), 4.69 (d, ^3^*J*_H,H_ = 4.4 Hz, 2H, Ph-CH_2_), 2.55 (t, ^3^*J*_H,H_ = 7.5 Hz, 2H, H-b), 1.75 (p, ^3^*J*_H,H_ = 7.5 Hz, 1H, H-c), 1.46 – 1.37 (m, 2H, H-d), 1.37 – 1.21 (m, 14H, He, H-f, H-g, H-h, H-i, H-j, H-k), 0.88 (t, ^3^*J*_H,H_ = 7.0 Hz, 1H, H-l). – ^**13**^**C-NMR** *δ* [ppm] (151 MHz, CDCl_3_): 172.55 (C-a), 150.34 (C-1), 138.48 (C-4), 128.21 (C-3), 121.87 (C-2), 64.97 (Ph-CH_2_), 34.55 (C-b), 32.06, 29.75, 29.61, 29.48, 29.40, 29.26, 25.10, 22.83 (C-c, C-d, C-e, C-f, C-g, C-h, C-i, C-j, C-k), 14.27 (C-l).). – **HRMS**: (ESI^+^, m/z): calcd.: 329.2087 [M+Na^+^], found: 329.2074 [M+Na^+^].

#### 4-Dodecanoyloxybenzyl-methyl-*H*-phosphonate

The reaction was performed under nitrogen atmosphere and dry conditions. 0.050 mL (0.83 mmol, 0.83 eq.) diphenyl phosphite (DPP) was dissolved in 9 mL pyridine. It was cooled to 0 °C and 0.31 g (1.0 mmol, 1.0 eq.) of the C_11_-AB mask **45** was added. The reaction mixture was stirred for 1.5 h at 0 °C and 0.060 mL (1.5 mmol, 1.0 eq.) MeOH was added dropwise. After stirring for another 2 h at room temperature, all volatiles were removed in vacuo. The residue was coevaporated for three times with toluene and CH_2_Cl_2_, respectively. The crude product was purified by silica gel chromatography (PE/EtOAc 1:1, 0.5 % AcOH) to afford 71 % (0.23 g, 0.61 mmol) of the product as a colourless solid. ^**1**^**H-NMR** (500 MHz, CDCl_3_): *δ* [ppm] = 7.44-7.39 (m, 2H, H-3), 7.12-7.08 (m, 2H, H-2), 6.85 (d, ^1^*J*_H,P_ = 703 Hz, 1H, H-6), 5.10 (d, ^3^*J*_H,H_ = 9.7 Hz, 2H, H-5), 3.72 (d, ^3^*J*_H,H_ = 12.0 Hz, 3H, H-7), 2.55 (t, ^3^*J*_H,H_ = 7.5 Hz, 2H, H-b), 1.75 (p, ^3^*J*_H,H_ = 7.5 Hz, 2H, H-c), 1.44-1.23 (m, 16H, H-d, H-e, H-f, H-g, H-i, H-j, H-k), 0.88 (t, ^3^*J*_H,H_ = 6.9 Hz, 3H, H-l). – ^**13**^**C-NMR** (125 MHz, CDCl_3_): *δ* [ppm] = 172.3 (C-a), 151.2 (C-1), 133.2 (d, ^3^*J*_C,P_ = 5.8 Hz, C-4), 129.4 (C-3), 122.1 (C-2), 66.9 (d, ^2^*J*_C,P_ = 5.6 Hz, C-5), 52.1 (d, ^2^*J*_C,P_ = 6.0 Hz, C-7), 34.5 (C-b), 32.1 (C-d, C-e, C-f, C-g, C-h, C-i, C-j, C-k), 29.7 (C-d, C-e, C-f, C-g, C-h, C-i, C-j, C-k), 29.6 (C-d, C-e, C-f, C-g, C-h, C-i, C-j, C-k), 29.5 (C-d, C-e, C-f, C-g, C-h, C-i, C-j, C-k), 29.4 (C-d, C-e, C-f, C-g, C-h, C-i, C-j, C-k), 29.3 (C-d, C-e, C-f, C-g, C-h, C-i, C-j, C-k), 25.1 (C-c), 22.8 (C-d, C-e, C-f, C-g, C-h, C-i, C-j, C-k), 14.3 (C-l). – ^**31**^**P-NMR** (243 MHz, CDCl_3_): *δ* [ppm] = 9.10. –– **HRMS** (ESI^+^, m/z): calcd.: 407.1958 [M+Na]^+^, found: 407.1953 [M+Na]^+^.

#### Prodrug of β-methyl-GS-441524-DP 5

The reaction was performed under nitrogen atmosphere and dry conditions. First, 44 mg (0.11 mmol, 1.0 eq.) *H-*phosphonate was dissolved in 4 mL CH_3_CN, and 38 mg (0.28 mmol, 2.5 eq.) *N*-chlorosuccinimide was added in one portion. The reaction mixture was stirred for 16 h at 50 °C. Subsequently, 50 mg (0.11 mmol, 1.0 eq.) GS-441524-MP **40** was suspended in 5 mL CH_3_CN and 0.040 mL (0.28 mmol, 2.5 eq.) triethylamine was added. To this suspension, the activated phosphochloridate was added dropwise. The reaction mixture was stirred for 9 h at room temperature; all volatiles were removed in vacuo and then purified by automated RP18 flash chromatography (H_2_O/CH_3_CN 0-100 % Vol., 0–50 min; flow rate 20 mL/min). This was followed by conversion to the ammonium and triethylammonium forms using a cation-exchange resin (Dowex 50WX8), with subsequent automated RP18 flash chromatography (H_2_O/CH_3_CN 0-100 % Vol., 0-50 min; flow rate 20 mL/min), respectively. Product-containing fractions were freeze-dried to afford 66 mg (0.082 mmol) of the diphosphate as a colourless solid (73 %) as its triethylammonium salt. ^**1**^**H-NMR** (600 MHz, DMSO-*d*_6_): *δ* [ppm] = 7.92 (s, 1H, H-2), 7.35 (d, ^3^*J*_H,H_ = 8.5 Hz, 2H, H-3′′), 7.05-7.00 (m, 2H, H-2′′), 6.91 -6.86 (m, 2H, H-7, H-8), 5.10-5.00 (m, 1H, H-2′), 4.85-4.80 (m, 2H, H-5), 4.57 (d, ^3^*J*_H,H_ = 4.8 Hz, H-4′), 4.16 (dt, ^3^*J*_H,H_ = 7.3 Hz, ^3^*J*_H,H_ = 4.0 Hz, 1H, H-3′), 4.10-3.98 (m, 4H, H-5′, CH_3_), 2.55 (t, ^3^*J*_H,H_ = 7.4 Hz, 2H, H-b), 1.67-1.58 (m, 2H, H-c), 1.39-1.19 (m, 16H, H-d, H-e, H-f, H-g, H-h, H-i, H-j, H-k), 0.85 (z, ^3^*J*_H,H_ = 7.0 Hz, 3H, H-l). – ^**13**^**C-NMR** (151 MHz, DMSO-*d*_6_): *δ* [ppm] = 171.8 (C-a), 149.6 (C-1′′), 147.8 (C-2), 136.4 (C-4′′), 129.3 (C-9), 128.4 (C-3′′), 124.2 (C-1′), 121.4 (C-2′′), 116.8 (C-5), 110.4 (C-7 od. C-8), 100.9 (C-7 od. C-8), 82.8 (C-3′), 74.4 (C-4′), 69.5 (CH_3_), 68.0 (C-2′), 66.0 (C-5′′), 64.5 (C-5′), 33.5 (C-b), 31.3 (C-d, C-e, C-f, C-g, C-h, C-i, C-j, C-k), 30.7 (C-d, C-e, C-f, C-g, C-h, C-i, C-j, C-k), 29.0 (C-d, C-e, C-f, C-g, C-h, C-i, C-j, C-k), 28.9 (C-d, C-e, C-f, C-g, C-h, C-i, C-j, C-k), 28.7 (C-d, C-e, C-f, C-g, C-h, C-i, C-j, C-k), 28.7 (C-d, C-e, C-f, C-g, C-h, C-i, C-j, C-k), 28.7 (C-d, C-e, C-f, C-g, C-h, C-i, C-j, C-k), 28.4 (C-d, C-e, C-f, C-g, C-h, C-i, C-j, C-k), 24.3 (C-c), 22.1 (C-d, C-e, C-f, C-g, C-h, C-i, C-j, C-k), 14.0 (C-l). – ^**31**^**P-NMR** (243 MHz, DMSO-*d*_6_): *δ* [ppm] = -11.41 (q, ^2^*J*_P,P_ = 15.7 Hz). – **HRMS** (ESI^-^, m/z): calcd.: 752.2467 [M-H]^-^, found: 752.2457 [M-H]^-^.

#### Triethylammonium ethylphosphonate 46

0.21 g (1.4 mmol, 1.0 eq.) ethylphosphonic dichloride was dissolved in 20 mL water and 0.04 mL (1.4 mmol, 1.0 eq.) conc. hydrochloric acid was added. After stirring for one hour at 55 °C, all volatiles were removed in vacuo. This was followed by conversion to the triethylammonium form using a cation-exchange resin (Dowex 50WX8), with subsequent automated RP18 flash chromatography (H_2_O/CH_3_CN 0-100 % Vol., 0-50 min; flow rate 20 mL/min). Product-containing fractions were freeze-dried to afford 0.19 g (1.2 mmol) of the diphosphate as a colourless oil (84 %).^**1**^**H-NMR** (600 MHz, DMSO-*d*_6_): *δ* [ppm] = 1.48 (dq, ^2^*J*_H,H_ = 17.8 Hz, ^3^*J*_H,H_ = 7.7 Hz, 2H, H-1), 1.00 (dt, ^2^*J*_H,H_ = 18.9 Hz, ^3^*J*_H,H_ = 7.7 Hz, 3H, H-2). – ^**13**^**C-NMR** (151 MHz, DMSO-*d*_6_): *δ* [ppm] = 21.0 (C-1), 20.1 (C-1), 7.2 (C-2), 7.2 (C-2). – ^**31**^**P-NMR** (202 MHz, DMSO-*d*_6_): *δ* [ppm] = 27.68. – **HRMS** (ESI^-^, m/z): calcd.: 109.0060 [M-H]^-^, found: 109.0062 [M-H]^-^.

#### β-Ethylphosphonate phosphate GS-441524 (ST161) 42

The reaction was carried out according to general procedure 2, with 50 mg (0.14 mmol, 1.0 eq.) GS-441524-MP **40**, 5 mL CH_3_CN, 0.30 mL (2.2 mmol, 16 eq.) triethylamine and 0.19 mL (1.4 mmol, 10 eq.) TFAA, dissolved in 5 mL CH_3_CN. The residue was redissolved in 5 mL CH_3_CN and 0.19 mL (1.4 mmol, 10 eq.) triethylamine, 0.060 mL (0.81 mmol, 6.0 eq.) NMI were added. After a stirring time of 10 min, 33 mg (0.20 mmol, 1.5 eq.) of ethyl phosphonate, suspended in 3.5 mL CH_3_CN, was added dropwise. The reaction mixture was stirred for 7 h at room temperature and then purified by automated RP18 flash chromatography (H_2_O/CH_3_CN 0-100 % Vol., 0-50 min; flow rate 20 mL/min). The product was obtained as a colourless solid (51 mg, 0.085 mmol, 42 %) as its triethylammonium salt. ^**1**^**H-NMR** (500 MHz, D_2_O): *δ* [ppm] = 7.95 (s, 1H, H-2), 7.03 (d, ^3^*J*_H,H_ = 4.8 Hz, 1H, H-7), 7.00 (d, ^3^*J*_H,H_ = 4.7 Hz, 1H, H-8), 4.96 (d, ^3^*J*_H,H_ = 5.4 Hz, 1H, H-2′), 4.53-4.50 (m, 1H, H-4′), 4.49-4.45 (m, 1H, H-3′), 4.15-4.04 (m, 2H, H-5′), 1.57 (dq, ^2^*J*_H,P_ = 17.8 Hz, ^3^*J*_H,H_ = 7.7 Hz, 2H, H-a), 0.95 (dt, ^3^*J*_H,P_ = 20.0 Hz, ^3^*J*_H,H_ = 7.7 Hz, 3H, H-b). – ^**13**^**C-NMR** (125 MHz, D_2_O): *δ* [ppm] = 154.2 (C-6), 144.7 (C-2), 124.8 (C-9), 117.3 (CN), 116.2 (C-5), 112.0 (C-7), 104.6 (C-8), 85.4 (d, ^3^*J*_C,P_ = 9.1 Hz, C-4′), 77.0 (C-1′), 75.3 (C-2′), 70.8 (C-3′), 64.9 (d, ^2^*J*_C,P_ = 5.5 Hz, C-5′), 21.1 (d, ^1^*J*_C,P_ = 138.5 Hz, C-a), 7.03 (d, ^2^*J*_C,P_ = 6.6 Hz, C-b). ^**31**^**P-NMR** (243 MHz, D_2_O): *δ* [ppm] = 21.88 (d, ^2^*J*_P,P_ = 26.5 Hz, P_β_), -11.60 (d, ^2^*J*_P,P_ = 26.9 Hz, P_α_). – **HRMS** (ESI^-^, m/z): calcd.: 464.0378 [M-H]^-^, found: 464.0376 [M-H]^-^.

#### Triethylammonium (4-dodecanoyloxybenzyl)-ethyl phosphonate 47

The reaction was performed under nitrogen atmosphere and dry conditions. First, 0.20 g (1.4 mmol, 1.0 eq.) ethylphosphonic dichloride was dissolved in 20 mL pyridine and 0.38 g (1.2 mmol, 0.90 eq.) of the C_11_-AB mask **45 **was added portionwise. After stirring for three h at room temperature, 10 mL of a 1 M TEAB buffer was added. Then all volatiles were removed and the residue was coevaporated three times with toluol and CH_2_Cl_2_, respectively. The crude product was purified by automated RP18 flash chromatography (H_2_O/CH_3_CN 0-100 % Vol., 0-50 min; flow rate 20 mL/min) to afford 0.20 g (0.46 mmol) of the product as a colourless oil (34 %). ^**1**^**H-NMR** (400 MHz, CD_3_CN): *δ* [ppm] = 7.44-7.36 (m, 2H, H-3), 7.06-6.99 (m, 2H, H-2), 4.88 (d, ^3^*J*_H,H_ = 7.4 Hz, 2H, H-5), 2.54 (t, ^3^*J*_H,H_ = 7.4 Hz, 2H, H-b), 1.69 (pseudo-p, ^3^*J*_H,H_ = 7.4 Hz, 2H, H-c), 1.54 (dq, ^2^*J*_H,P_ = 17.5 Hz, ^3^*J*_H,H_ = 7.7 Hz, 2H, H-1′), 1.44-1.25 (m, 16H, H-d, H-e, H-f, H-g, H-h, H-i, H-j, H-k), 1.04 (dt, ^3^*J*_H,P_ = 18.4 Hz, ^3^*J*_H,H_ = 7.7 Hz, 3H, H-2′), 0.92-0.84 (m, 3H, H-l). – ^**13**^**C-NMR** (101 MHz, CD_3_CN): *δ* [ppm] = 173.3 (C-a), 151.6 (C-1), 137.6 (C-4), 129.4 (C-3), 122.6 (C-2), 65.8 (d, ^2^*J*_C,P_ = 5.3 Hz, C-5), 34.7 (C-b), 32.6 (C-d, C-e, C-f, C-g, C-h, C-i, C-j, C-k), 30.3 (C-d, C-e, C-f, C-g, C-h, C-i, C-j, C-k), 30.3 (C-d, C-e, C-f, C-g, C-h, C-i, C-j, C-k), 30.2 (C-d, C-e, C-f, C-g, C-h, C-i, C-j, C-k), 30.0 (C-d, C-e, C-f, C-g, C-h, C-i, C-j, C-k), 29.9 (C-d, C-e, C-f, C-g, C-h, C-i, C-j, C-k), 29.7 (C-d, C-e, C-f, C-g, C-h, C-i, C-j, C-k), 25.6 (C-c), 23.4 (C-d, C-e, C-f, C-g, C-h, C-i, C-j, C-k), 20.9 (C-1′), 14.4 (C-l), 8.0 (d, ^2^*J*_C,P_ = 6.7 Hz, C-2′). ^**31**^**P-NMR** (162 MHz, CD_3_CN): *δ* [ppm] = 26.53. – **HRMS** (ESI^-^, m/z): calcd.: 397.2149 [M-H]^-^, found: 397.2152 [M-H]^-^.

#### β-C11-AB-(ethyl)-GS-441524-phosphonate-phosphate (ST166) 6

The reaction was carried out according to general procedure 2, with 50 mg (0.14 mmol, 1.0 eq.) GS-441524-MP **40**, 5 mL CH_3_CN, 0.30 mL (2.2 mmol, 16 eq.) triethylamine and 0.19 mL (1.4 mmol, 10 eq.) TFAA, dissolved in 5 mL CH_3_CN. The residue was redissolved in 5 mL CH_3_CN and 0.19 mL (1.4 mmol, 10 eq.) triethylamine, 0.060 mL (0.81 mmol, 6.0 eq.) NMI were added. After a stirring time of 10 min, 75 mg (0.16 mmol, 1.2 eq.) of (4-dodecanoyloxybenzyl)-ethyl phosphonate **47**, suspended in 7.5 mL CH_3_CN/DMF (2:1), was added dropwise. The reaction mixture was stirred for 7 h at room temperature and then purified by automated RP18 flash chromatography (H_2_O/CH_3_CN 0-100 % Vol., 0-50 min; flow rate 20 mL/min). The product was obtained as a colourless solid (62 mg, 0.079 mmol, 49 %) as its triethylammonium salt. ^**1**^**H-NMR** (600 MHz, D_2_O): *δ* [ppm] = 7.77 (pseudo-d, ^3^*J*_H,H_ = 6.2 Hz, 1H, H-2), 7.25-7.14 (m, 2H, H-3′′), 6.90-6.71 (m, 4H, H-2′′, H-7, H-8), 4.00-4.86 (m, 2H, H-5′′), 4.49-4.40 (m, 1H, H-4′), 4.39-4.31 (m, 1H, H-3′), 4.28-4.09 (m, 2H, H-5′), 2.40-2.28 (m, 2H, H-b), 1.90-1.70 (m, 2H, H-c), 1.58-1.46 (m, 2H, H-10), 1.29-1.12 (m, 16H, H-d, H-e, H-f, H-g, H-h, H-i, H-j, H-k), 1.02-0.89 (m, 3H, H-11), 0.85-0.77 (m, 3H, H-l). – ^**13**^**C-NMR** (151 MHz, D_2_O): *δ* [ppm] = 172.1 (C-a), 152.2 (C-2), 151.2 (C-6), 150.1 (C-1′′), 133.2 (C-4′′), 128.7 (C-3′′), 125.3 (C-9), 121.4 (C-2′′), 113.3 (C-5), 111.8 (C-7, C-8), 83.4 (C-4′), 74.8 (C-2′), 69.6 (C-3′), 66.3 (C-5′′), 65.1 (C-5′), 33.9 (C-b), 31.9 (C-d, C-e, C-f, C-g, C-h, C-i, C-j, C-k), 29.7 (C-d, C-e, C-f, C-g, C-h, C-i, C-j, C-k), 29.7 (C-d, C-e, C-f, C-g, C-h, C-i, C-j, C-k), 29.4 (C-d, C-e, C-f, C-g, C-h, C-i, C-j, C-k), 29.4 (C-d, C-e, C-f, C-g, C-h, C-i, C-j, C-k), 29.1 (C-d, C-e, C-f, C-g, C-h, C-i, C-j, C-k), 24.6 (C-10), 22.6 (C-d, C-e, C-f, C-g, C-h, C-i, C-j, C-k), 18.3 (C-c), 13.8 (C-l), 8.2 (C-d, C-e, C-f, C-g, C-h, C-i, C-j, C-k), 5.7 (C-11). ^**31**^**P-NMR** (243 MHz, D_2_O): *δ* [ppm] = 27.30 (P_β_), -11.75 (P_α_). – **HRMS** (ESI^+^, m/z): calcd.: 752.2820 [M + H]^+^, found: 752.2821 [M + H]^+^.

#### Bis-*O*-(9*H*-fluoren-9-ylmethyl-*N,N*-di*iso*propylamino phosphoramidite (Fm amidite)

The reaction was performed according to Pahnke and Meier^36^. ^**1**^**H-NMR:** *δ* [ppm] (400 MHz, CDCl_3_): 7.78 – 7.73 (m, 4H, H-4, H-7), 7.64 (m, 4H, H-1, H-10), 7.38 (m, 4H, H-3, H-8), 7.29 (m, 4H, H-2, H-9), 4.18 (t, ^3^*J*_H,H_ = 7.1 Hz, 2H, H-12), 4.00 (dt, ^3^*J*_H,H_ = 9.9, ^3^*J*_H,H_ = 6.8 Hz, 2H, Fm-CH_2_), 3.80 (dt, ^3^*J*_H,H_ = , 7.3 Hz, 2H, Fm-CH_2_), 3.65 (m, 2H, *i*Pr-CH), 1.16 (d, ^3^*J*_H,H_ = 6.8 Hz, 6H, *i*Pr-CH_3_). – ^**13**^**C-NMR** *δ* [ppm] (126 MHz, CDCl_3_): *δ* 144.9 (C-11, C-13), 141.5, 141.4 (C-5, C-6), 127.5 (d, *J*_C,P_ = 5.1 Hz, C-3, C-8), 127.2, 127.0 (d, *J*_C,P_ = 5.1 Hz, C-2, C-9), 125.6, 125.3 (C-1, C-10), 121.1, 119.9 (d, *J*_C,P_ = 7.8 Hz, C-4, C-7), 66.0 (d, *J*_C,P_ = 17.0 Hz, Fm-CH_2_), 49.3 (d, *J*_C,P_ = 7.4 Hz, C-12), 43.2 (d, *J*_C,P_ = 12.4 Hz, *i*Pr-CH), 24.8 (d, *J*_C,P_ = 7.4 Hz, *i*Pr-CH_3_). – ^**31**^**P-NMR**: *δ* [ppm] (202 MHz, CDCl_3_): 147.40. – **HRMS** (ESI^+^, m/z): calcd.: 522.2556 [M + H^+^], found: 522.2581 [M + H^+^].

#### 5-*O*-Trityl-D-ribono-1,4-lactone

The reaction was performed according to Drown and coworkers^32^. ^**1**^**H-NMR**: *δ* [ppm] (500 MHz, DMSO-*d*_6_): 7.40 – 7.31 (m, 12H, H-8, H-9), 7.31– 7.27 (m, 3H, H-10), 5.90 (d, ^3^*J*_H,H_ = 7.6 Hz, 1H, 1-OH), 5.43 (d, ^4^*J*_H,H_ = 4.0 Hz, 1H, 2-OH), 4.51 (dd, ^3^*J*_H,H_ = 7.6, ^4^*J*_H,H_ = 5.4 Hz, 1H, H-2), 4.36 (m, 1H, H-4), 4.00 (m, 1H, H-3), 3.35 (m, 1H, H-5), 3.13 (dd, ^3^*J*_H,H_ = 11.0, ^4^*J*_H,H_ = 3.8 Hz, 1H, H-5). – ^**13**^**C-NMR**: *δ* [ppm] (126 MHz, DMSO-d_6_): 177.1 (C-1), 143.0 (C-7), 128.1 (C-8, C-9), 128.0 (C-8, C-9), 127.2 (C-10), 86.6 (C-6), 83.3 (C-4), 69.3 (C-3), 68.5 (C-2), 63.0 (C-5). – **HRMS** (ESI^+^, m/z): calcd.: 413.1359 [M+Na^+^], found: 413.1366 [M+Na^+^].

#### 2,3-Bis-*O*-*tert*-Butyldimethylsilyl-5-*O*-trityl-D-ribono-1,4-lactone

The reaction was performed according to Drown and coworkers^32^. ^**1**^**H-NMR**: *δ* [ppm] (500 MHz, DMSO-*d*_6_): 7.37-7.34 (m, 12H, H-8, H-9), 7.32-7.27 (m, 3H, H-10), 4.68 (d, ^3^*J*_H,H_ = 5.2 Hz, 1H, H-2), 4.30 (m, 1H, H-4), 3.97 (m, 1H, H-3), 3.54 (dd, ^3^*J*_H,H_ = 11.5, ^4^*J*_H,H_ = 3.6 Hz, 1H, H-5), 3.24 (dd, ^3^*J*_H,H_ = 11.5, ^4^*J*_H,H_ = 3.4 Hz, 1H, H-5), 0.88 (s, 9H, H-13), 0.77 (s, 9H, H-13), 0.10 (s, 3H, H-11), 0.06 (s, 3H, H-11), -0.03 (s, 3H, H-11), -0.06 (s, 3H, H-11). ^**13**^**C-NMR**: *δ* [ppm] (126 MHz, DMSO-d_6_): 174.3 (C-1), 142.9 (C-1), 128.0 (C-8, C-9) 127.3 (C-10), 86.9 (C-6), 83.9 (C-4), 71.4 (C-3), 69.8 (C-2), 62.0 (C-5), 25.5 (C-13), 25.4 (C-13), 17.9 (C-12), 17.6 (C-12), –5.0 (C-11), –4.9 (C-11). – **HRMS** (ESI^+^, m/z): calcd.: 641. 3089 [M+Na^+^], found: 641.3110 [M+Na^+^].

#### 2′,3′-Bis-*O*-*tert*-Butyldimethylsilyl-5′-*O*-trityl-D-ribose

The reaction was performed according to Drown and coworkers^32^. ^**1**^**H-NMR**: *δ* [ppm] (500 MHz, CDCl_3_): 7.44 – 7.41 (m, 5H, H-8, H-9), 7.33 – 7.28 (m, 7H, H-8, H-9), 7.27 – 7.22 (m, 3H, H-10), 5.12 (dd, ^3^*J*_H,H_ = 11.3, ^4^*J*_H,H_ = 4.3 Hz, 1H, H-1´), 4.23 (d, ^3^*J*_H,H_ = 11.4 Hz, 1H, OH), 4.19 (m, 1H, H-4´), 4.15 (m, 1H, H-2´), 3.91 (m, 1H, H-3´), 3.26 (dd, ^3^*J*_H,H_ = 10.3, ^4^*J*_H,H_ = 4.9 Hz, 1H, H-5´), 3.11 (dd, ^3^*J*_H,H_ = 10.3, ^4^*J*_H,H_ = 3.4 Hz, 1H, H-5´), 0.92 (s, 9H, H-13), 0.84 (s, 9H, H-13), 0.12 (s, 3H, H-11), 0.10 (s, 3H, H-11), 0.04 (s, 3H, H-11), -0.02 (s, 3H, H-11). – ^**13**^**C-NMR** *δ* [ppm] (126 MHz, CDCl_3_): 143.8 (C-7), 128.8 (C-8, C-9), 128.0 (C-8, C-9), 127.3 (C-10), 97.9 (C-1), 87.1 (C-6), 84.4 (C-4), 74.6 (C-3), 72.6 (C-2), 63.8 (C-5), 26.1 (C-13), 25.9 (C-13), 18.2 (C-12), –4.68 (C-11), –4,92 (C-11). – **HRMS** (ESI^+^, m/z): calcd.: 643.3245 [M+Na^+^], found: 643.3255 [M+Na^+^].

#### 2′,3′-Bis-*O*-*tert*-butyldimethylsilyl-1′-*O-tert*-butyldiphenylsilyl-5′-*O*-trityl-D-ribose

The reaction was performed under nitrogen atmosphere and dry conditions. First, 0.95 g (1.5 mmol, 1.0 eq.) of the ribose, 0.26 g (3.8 mmol, 2.5 eq.) imidazole and 1.3 g (4.7 mmol, 3.0 eq.) *tert*-butyldiphenylsilyl chloride were dissolved in 20 mL DMF. The reaction mixture was stirred for 24 h at room temperature. Subsequently, the reaction was diluted with Et_2_O and a saturated NH_4_Cl solution was added. The organic layer was washed three times with a saturated NaHCO_3_ solution and once with brine. The combined organic layers were dried over Na_2_SO_4_ and the solvent was removed under reduced pressure. The crude product was purified by silica gel chromatography (PE/EtOAc 50:1 → 10:1) to afford 0.84 g (0.97 mmol) of the product as a colourless solid (64 %). ^**1**^**H-NMR** (400 MHz, CDCl_3_): *δ* [ppm] = 7.83–7.76 (m, 2H, H-2), 7.69-7.65 (m, 2H, H-2), 7.58-7.52 (m, 6H, H-9), 7.45-7.40 (m, 2H, H-4), 7.39-7.33 (m, 4H, H-3), 7.28.7.21 (m, 6H, H-10), 7.21-7.15 (m, 3H, H-11), 5.14 (s, 1H, H-1′), 4.36 (dd, ^3^*J*_H,H_ = 8.5 Hz, ^4^*J*_H,H_ = 3.6 Hz, 1H, H-3′), 4.18-4.09 (m, 1H, H-4′), 3.77 (d, ^4^*J*_H,H_ = 3.5 Hz, 1H, H-2′), 3.41 (dd, ^2^*J*_H,H_ = 10.1 Hz, ^4^*J*_H,H_ = 2.0 Hz, 1H, H-5′), 3.13 (dd, ^2^*J*_H,H_ = 10.1 Hz, ^3^*J*_H,H_ = 6.2 Hz, 1H, H-5′), 1.00 (s, 9H, H-6), 0.75 (s, C(C**H**_3_)_3_-a), 0.74 (s, 9H, C(C**H**_3_)_3_-b), -0.04 (s, 3H, Si(C**H**_3_)_2_-a), -0.14 (s, 3H, Si(C**H**_3_)_2_-a), -0.16 (s, 3H, Si(C**H**_3_)_2_-b), -0.42 (s, 3H, Si(C**H**_3_)_2_-b). – ^**13**^**C-NMR** (101 MHz, CDCl_3_): *δ* [ppm] = 144.3 (C-8), 135.9, 135.7 (C-2), 134.1, 132.9 (C-1), 130.0, 129.9 (C-4), 129.0 (C-9), 128.0 (C-3), 127.8 (C-10), 126.9 (C-11), 102.1 (C-1′), 86.5 (C-7), 80.6 (C-4′), 77.8 (C-2′), 72.1 (C-3′), 65.4 (C-5′), 26.9 (C-6), 26.0 (C(**C**H_3_)_3_-a), 25.9 (C(**C**H_3_)_3_-b), 19.3 (C-5), 18.2 (**C**(CH_3_)_3_-a), 18.0 (**C**(CH_3_)_3_-b), -4.0, -4.6, -4.9, -5.0 (Si(**C**H_3_)_2_-a, Si(**C**H_3_)_2_-b). – **HRMS** (ESI^+^, m/z): calcd.: 881.4423 [M+Na]^+^, found: 881.4421 [M+Na]^+^.

#### 2′,3′-Bis-*O*-*tert*-butyldimethylsilyl-1′-*O-tert*-butyldiphenylsilyl-D-ribose

The reaction was performed under nitrogen atmosphere and dry conditions. First, 0.25 g (0.29 mmol, 1.0 eq.) of the fully protected ribose was dissolved in CH_2_Cl_2_. Next, 0.16 mL (1.2 mmol, 4.0 eq.) TFAA was dissolved in 0.54 mL CH_2_Cl_2_ and added to the ribose. Subsequently, 0.067 mL (0.90 mmol, 3.0 eq.) trifluoroacetic acid (TFA) was dissolved in 2 mL CH_2_Cl_2_ and added to the ribose dropwise. The reaction mixture was stirred for 25 min at room temperature and then neutralised by addition of 0.52 mL (3.8 mmol, 13 eq.) triethylamine and 1.1 mL MeOH. The mixture was stirred for one more hour and then added to into a saturated NH_4_Cl solution. It was extracted twice with CH_2_Cl_2_, dried over Na_2_SO_4_ and the solvent was removed under reduced pressure. The crude product was purified by silica gel chromatography (PE/EtOAc 15:1) to afford 0.096 g (0.16 mmol) of the product as a colourless solid (53 %). ^**1**^**H-NMR** (500 MHz, CDCl_3_): *δ* [ppm] = 7.72–7.67 (m, 2H, H-2), 7.65-7.60 (m, 2H, H-2), 7.48-7.41 (m, 3H, H-3, H-4), 7.41-7.35 (m, 3H, H-3, H-4), 5.04 (s, 1H, H-1′), 4.60 (dd, ^3^*J*_H,H_ = 8.3 Hz, ^4^*J*_H,H_ = 3.8 Hz, 1H, H-3′), 4.03 (ddd, ^3^*J*_H,H_ = 8.3 Hz, ^3^*J*_H,H_ = 2.5 Hz, ^3^*J*_H,H_ = 2.5 Hz, 1H, H-4′), 3.86 (dd, ^2^*J*_H,H_ = 12.1 Hz, ^3^*J*_H,H_ = 2.5 Hz, 1H, H-5′), 3.78 (d, ^4^*J*_H,H_ = 3.6 Hz, 1H, H-2′), 3.60 (dd, ^2^*J*_H,H_ = 12.2 Hz, ^3^*J*_H,H_ = 2.5 Hz, 1H, H-5′), 1.07 (s, 9H, H-6), 0.91 (s, 9H, C(C**H**_3_)_3_-a), 0.75 (s, 9H, C(C**H**_3_)_3_-b), 0.14 (s, 3H, Si(C**H**_3_)_2_-a), 0.10 (s, 3H, Si(C**H**_3_)_2_-a), -0.11 (s, 3H, Si(C**H**_3_)_2_-b), -0.34 (s, 3H, Si(C**H**_3_)_2_-b). – ^**13**^**C-NMR** (125 MHz, CDCl_3_): *δ* [ppm] = 135.7 135.8 (C-2), 133.3, 132.6 (C-1), 128.1, 128.1 (C-3, C-4), 130.3, 130.2 (C-3, C-4), 101.8 (C-1′), 82.0 (C-4′), 78.2 (C-2′), 70.3 (C-3′), 60.8 (C-5′), 26.9 (C-6), 26.1 (C(**C**H_3_)_3_-a), 25.8 (C(**C**H_3_)_3_-b), 19.2 (C-5), 18.3 (**C**(CH_3_)_3_-a), 18.1 (**C**(CH_3_)_3_-b), -4.1, -4.2, -4.7, -4.8 (Si(**C**H_3_)_2_-a, Si(**C**H_3_)_2_-b). – **HRMS** (ESI^+^, m/z): calcd.: 639.3328 [M+Na]^+^, found: 639.3326 [M+Na]^+^.

#### Bis-*O*-(9*H*-fluoren-9-ylmethyl-2′,3′-bis-*O*-tert-butyldimethylsilyl-1′-*O-tert*-butyldiphenylsilyl-D-ribose-5′-monophosphate

The reaction was performed under nitrogen atmosphere and dry conditions. 0.090 (0.14 mmol, 1.0 eq.) ribose and 0.093 g (0.18 mmol, 1.2 eq.) Fm-amidite were dissolved in 2.9 mL CH_2_Cl_2_. 1.2 mL 4,5-Dicyanoimidazole (0.25 mol/L in acetonitrile) was added dropwise and the reaction mixture was stirred for 3 h at room temperature. Subsequently, the reaction mixture was cooled to 0 °C and 0.15 mL (1.4 mmol, 7.8 eq.) *tert*-butyl hydroperoxide was added dropwise. The reaction was stirred for another 1 hour, water was added and then extracted with CH_2_Cl_2_ three times. The combined organic layers were dried over Na_2_SO_4_ and the solvent was removed in vacuo. The crude product was purified by silica gel chromatography (PE/EtOAc 20:1) to afford 0.15 g (0.14 mmol) of the product as a colourless oil (98 %). ^**1**^**H-NMR** (600 MHz, CDCl_3_): *δ* [ppm] = 7.74-7.65 (m, 6H, H-4′′, H-5′′, H-2), 7.61-7.58 (m, 2H, H-2), 7.51-7.42 (m, 4H, H-3), 7.41-7.26 (m, 10H, H-4, H-1′′, H-3′′, H-6′′, H-8′′), 7.24-7.16 (m, 4H, H-2′′, H-7′′), 5.01 (s, 1H, H-1′), 4.38-4.34 (m, 1H, H-3′), 4.34-4.28 (m, 1H, H-5′), 4.28-4.17 (m, Fm-C**H**_2_), 4.17-4.05 (m, 4H, H-4′, H-5′, H-9′′), 3.82 (d, ^4^*J*_H,H_ = 3.6 Hz, 1H, H-2′), 1.01 (s, 9H, H-6), 0.89 (s, 9H, C(C**H**_3_)_3_-a), 0.74 (s, 9H, C(C**H**_3_)_3_-b), 0.10 (s, 3H, Si(C**H**_3_)_2_-a), 0.09 (s, 3H, Si(C**H**_3_)_2_-a), -0.09 (s, 3H, Si(C**H**_3_)_2_-b), -0.34 (s, 3H, Si(C**H**_3_)_2_-b). – ^**13**^**C-NMR** (151 MHz, CDCl_3_): *δ* [ppm] = 143.3, 143.2 (C-4′′, C-4b′′), 141.9, 141.5 (C-8a′′, C-9a′′), 135.9, 135.7 (C-2), 133.4, 132.8 (C-1), 130.1 (C-4), 130.1, 130.0, 128.0, 127.9, 127.8 (C-1′′, C-3′′, C-6′′, C-8′′), 127.2, (C-2′′ oder C-7′′), 125.5, 125.4 (C-3), 120.1, 120.0 (C-4′′, C-5′′), 102.0 (C-1′), 79.7 (C-4′), 77.8 (C-2), 72.1 (C-3′), 69.4 (d, ^2^*J*_C.P_ = 7.8 Hz, Fm-**C**H_2_), 68.9 (d, ^2^*J*_C.P_ = 5.3 Hz, C-5′), 48.0 (C-9′′), 26.9 (C-6), 26.1 (C(**C**H_3_)_3_-a), 25.8 (C(**C**H_3_)_3_-b), -4.1, -4.5 (Si(**C**H_3_)_2_-a), -4.6, -4.8, (Si(**C**H_3_)_2_-b). – ^**31**^**P-NMR** (243 MHz, CDCl_3_): *δ* [ppm] = -1.35. – **HRMS** (ESI^+^, m/z): calcd.: 1075.4556 [M+Na]^+^, found: 1075.4556 [M+Na]^+^.

#### **2′**,3′-Bis-*O*-*tert*-butyldimethylsilyl-1′-*O-tert*-butyldiphenylsilyl-D-ribose-5′-monophosphate

The reaction was performed under nitrogen atmosphere and dry conditions. First, 0.15 g (0.14 mmol, 1.0 eq.) of the Fm protected phosphate was dissolved in 1 mL CH_3_CN and then 0.25 mL Et_3_N were added (CH_3_CN/Et_3_N 4:1). The reaction mixture was stirred for 42 h at room temperature. Subsequently, all volatiles were removed in vacuo and coevaporated with toluol thrice. The crude product was purified by automated RP18 flash chromatography (H_2_O/CH_3_CN 0-100 % Vol., 0-50 min; flow rate 20 mL/min) to afford 0.038 g (0.046 mmol) of the product as a colourless oil (34 %). ^**1**^**H-NMR** (600 MHz, CDCl_3_): *δ* [ppm] = 7.74-7.65 (m, 6H, H-4′′, H-5′′, H-2), 7.61-7.58 (m, 2H, H-2), 7.51-7.42 (m, 4H, H-3), 7.41-7.26 (m, 10H, H-4, H-1′′, H-3′′, H-6′′, H-8′′), 7.24-7.16 (m, 4H, H-2′′, H-7′′), 5.01 (s, 1H, H-1′), 4.38-4.34 (m, 1H, H-3′), 4.34-4.28 (m, 1H, H-5′), 4.28-4.17 (m, Fm-C**H**_2_), 4.17-4.05 (m, 4H, H-4′, H-5′, H-9′′), 3.82 (d, ^4^*J*_H,H_ = 3.6 Hz, 1H, H-2′), 1.01 (s, 9H, H-6), 0.89 (s, 9H, C(C**H**_3_)_3_-a), 0.74 (s, 9H, C(C**H**_3_)_3_-b), 0.10 (s, 3H, Si(C**H**_3_)_2_-a), 0.09 (s, 3H, Si(C**H**_3_)_2_-a), -0.09 (s, 3H, Si(C**H**_3_)_2_-b), -0.34 (s, 3H, Si(C**H**_3_)_2_-b). – ^**13**^**C-NMR** (151 MHz, CDCl_3_): *δ* [ppm] = 143.3, 143.2 (C-4′′, C-4b′′), 141.9, 141.5 (C-8a′′, C-9a′′), 135.9, 135.7 (C-2), 133.4, 132.8 (C-1), 130.1 (C-4), 130.1, 130.0, 128.0, 127.9, 127.8 (C-1′′, C-3′′, C-6′′, C-8′′), 127.2, (C-2′′ oder C-7′′), 125.5, 125.4 (C-3), 120.1, 120.0 (C-4′′, C-5′′), 102.0 (C-1′), 79.7 (C-4′), 77.8 (C-2), 72.1 (C-3′), 69.4 (d, ^2^*J*_C.P_ = 7.8 Hz, Fm-**C**H_2_), 68.9 (d, ^2^*J*_C.P_ = 5.3 Hz, C-5′), 48.0 (C-9′′), 26.9 (C-6), 26.1 (C(**C**H_3_)_3_-a), 25.8 (C(**C**H_3_)_3_-b), -4.1, -4.5 (Si(**C**H_3_)_2_-a), -4.6, -4.8, (Si(**C**H_3_)_2_-b). – ^**31**^**P-NMR** (243 MHz, CDCl_3_): *δ* [ppm] = -1.35. – **HRMS** (ESI^+^, m/z): calcd.: 1075.4556 [M+Na]^+^, found: 1075.4556 [M+Na]^+^.

#### 7-Deazaadenosine diphosphate-2′,3′-bis-*O-tert*-butyldimethylsilyl-1′-*O-tert*-butyldiphenylsilyl ribose

The reaction was carried out according to general procedure 2, with 23 mg (0.060 mmol, 1.0 eq.) 7-Deazaadenosine monophosphate, 2.3 mL CH_3_CN, 0.13 mL (1.0 mmol, 16 eq.) triethylamine and 0.080 mL (0.60 mmol, 10 eq.) TFAA, dissolved in 2.3 mL CH_3_CN. The residue was redissolved in 2.3 mL CH_3_CN and 0.080 mL (0.60 mmol, 10 eq.) triethylamine, 0.030 mL (0.36 mmol, 6.0 eq.) NMI were added. After a stirring time of 10 min, 37 mg (0.048 mmol, 0.80 eq.) of the synthesized monophosphate dissolved in 4 mL CH_3_CN, was added dropwise. The reaction mixture was stirred for 7 h at room temperature and then purified by automated RP18 flash chromatography (H_2_O/CH_3_CN 0-100 % Vol., 0-50 min; flow rate 20 mL/min). The product was obtained as a colourless solid (29 mg, 0.027 mmol, 56 %). Not all 13 C signals could be assigned due to insufficient signal intensities. In some cases, the 13 C NMR signal shifts were taken from the 2D NMR spectra (HSQC, HMBC). ^**1**^**H-NMR** (600 MHz, DMSO-*d*_6_): *δ* [ppm] = 8.08-8.03 (m, 1H, H-2), 7.72-7.66 (m, 2H, H-10), 7.62-7.57 (m, 2H, H-10), 7.50-7.37 (m, 6H, H-11, H-12), 6.68-6.63 (m, 1H, H-7 od. H-8), 6.10-6.04 (m, 1H, H7 od. H-8), 5.34-5.29 (m, 1H, H-1′), 4.92 (pseudo-s, 1H, H-1′′), 4.35-4.27 (m, 2H, H-2′, H-3′′), 4.22-4.09 (m, 2H, H-3′, H-5′′), 4.06-3.98 (m, 3H, H-5′, H-4′′), 3.93-3.84 (m, 3H, H-4′, H-2′′, H-5′′), 1.00 (s, 9H, H-14), 0.87 (s, 9H, C(C**H**_3_)_3_-a), 0.74 (s, 9H, C(C**H**_3_)_3_-b), 0.11 (s, 3H, Si(C**H**_3_)_2_-a), 0.08 (s, 3H, Si(C**H**_3_)_2_-a), -0.07 (s, 3H, Si(C**H**_3_)_2_-b), -0.26 (s, 3H, Si(C**H**_3_)_2_-b). – ^**13**^**C-NMR** (151 MHz, DMSO-*d*_6_): *δ* [ppm] = 141.6 (C-2), 136.0, 135.2 (C-10), 128.4 (C-11, C-12), 100.7 (C-1′′), 84.3 (C-4′′), 79.4 (C-3′), 77.3 (C-4′, C-2′′), 75.9, 71.8 (C-2′, C-3′′), 67.2 (C-5′′), 61.3 (C-5′), 26.5 (C-14), 25.8 (C(**C**H_3_)_3_-a), 25.5 (C(**C**H_3_)_3_-b), 18.5 (C-13), 18.0 (**C**(CH_3_)_3_-a), 17.8 (**C**(CH_3_)_3_-b), -4.9 (Si(**C**H_3_)_2_-a), -5.2, (Si(**C**H_3_)_2_-b) – ^**31**^**P-NMR** (243 MHz, DMSO-*d*_6_): *δ* [ppm] = -10.70. – **HRMS** (ESI^-^, m/z): calcd.: 1023.3599 [M-H]^-^, found: 1023.3597 [M-H]^-^.

#### 7-Deazaadenosine DP ribose 22

The reaction was performed under nitrogen atmosphere and dry conditions. First, 25 mg (0.023 mmol, 1.0 eq.) of the protected 7-deaza-ADPR was dissolved in 1 mL THF and 1.4 mL (0.42 mmol, 36 eq.) TREAT-HF was added. The reaction mixture was stirred for 22 h at room temperature. After addition of 4 mL of a 1 M TEAB buffer was purified thrice by automated RP18 flash chromatography (0.050 M TEAB buffer/CH_3_CN 0–100 % Vol., 0-40 min; flow rate 20 mL/min). The product was obtained as a colourless solid (12 mg, 0.010 mmol, 39 %). Not all 13 C signals could be assigned due to insufficient signal intensities. In some cases, the ^13^C NMR signal shifts were taken from the 2D NMR spectra (HSQC, HMBC). ^**1**^**H-NMR** (400 MHz, D_2_O): *δ* [ppm] = 8.31 (s, 1H, H-2), 7.79 (d, ^4^*J*_H,H_ = 3.8 Hz, 1H, H-7 od. H-8), 6.96 (d, ^4^*J*_H,H_ = 3.8 Hz, 1H, H-7 od. H-8), 6.36 (d, ^3^*J*_H,H_ = 6.6 Hz, 1H, H-1′), 5.37–5.22 (m, 1H, H-1′′), 4.75–4.70 (m, 2H, H-2′, H-3′′), 4.57-4.53 (m, 1H, H-3′), 4.43-4.39 (m, 1H, H-4′), 4.34–4.30 (m, 1H, H-4′′), 4.26-4.19 (m, 2H, H-5′), 4.17-4.02 (m, 3H, H-2′′, H-5′′). – ^**13**^**C-NMR** (101 MHz, D_2_O): *δ* [ppm] = 135.1 (C-2), 124.5 (C-7 od. C-8), 103.0 (C-7 or C-8), 86.6 (C-1′), 84.4 (C-4′), 81.1 (C-4′′), 74.8 (C-2′′), 74.4 (C-2′, C-3′′), 70.4 (C-3′), 65.2 (C-5′).– ^**31**^**P-NMR** (162 MHz, D_2_O): *δ* [ppm] = -11.21, -11.42, -11.56. – **HRMS** (ESI^-^, m/z): calcd.: 557.0691 [M-H]^-^, found: 557.0691 [M-H]^-^.

#### Triethylammonium ethyl phosphate

The reaction was carried out according to general procedure 1, with 0.050 mL (0.87 mmol, 1.5 eq.) ethanol, 0.19 mL (0.58 mmol, 1.0 eq.) dibenzyl-*N,N*-di*iso*propyl phosphoramidite 15 mL 4,5-Dicyanoimidazole (0.2 mol/L in acetonitrile), 0.080 mL (0.87 mmol, 1.5 eq.) *tert*-butyl hydroperoxide and 11 mL CH_2_Cl_2_. The crude product was purified by silica gel chromatography (PE/EtOAc 3:1) to afford 0.10 g (0.35 mmol) of the product as a colourless oil (60 %).^**1**^**H-NMR** (600 MHz, CDCl_3_): *δ* [ppm] = 7.38-7.30 (m, 10H, H-Aryl), 5.04-4.99 (m, 4H, H-1), 4.10-4.03 (m, 2H, H-6), 1.27 (t, ^3^*J*_H,H_ = 7.0 Hz, 3H, H-7). – ^**13**^**C-NMR** (151 MHz, CDCl_3_): *δ* [ppm] = 136.1 (C-2), 128.7, 128.6, 128.1 (C-Aryl), 69.3 (d, ^2^*J*_C,P_ = 5.5 Hz, C-1), 64.2 (d, ^3^*J*_C,P_ = 5.7 Hz, C-6), 16.2 (d, ^3^*J*_C,P_ = 7.2 Hz, C-7). – ^**31**^**P-NMR** (243 MHz, CDCl_3_): *δ* [ppm] = -0.90. – **ATR-IR:** *ṽ* [cm^-1^] = 3489, 3065, 3034, 2983, 2897, 1899, 1498, 1456, 1381, 1264, 1214, 1165, 996, 966, 878, 732, 695, 599, 491. – **HRMS** (ESI^+^, m/z): calcd.: 329.0913 [M+Na]^+^, found: 329.0900 [M+Na]^+^.

The debenzylation of the intermediate was carried with 0.10 g (0.33 mmol, 1.0 eq.) dibenzylethyl phosphate, 7.5 mL EtOH, 0.75 mL water (alcohol/water 10:1), 0.090 mL (0.65 mmol, 2.0 eq.) triethylamine and 20 mg (20 wt.-%) palladium hydroxide on activated charcoal and 49 mg (0.26 mmol) of the product was obtained as a colourless oil (80 %). ^**1**^**H-NMR** (600 MHz, DMSO-*d*_6_): *δ* [ppm] = 3.77-3.69 (m, 2H, H-1), 1.13 (t, ^3^*J*_H,H_ = 7.1 Hz, 3H, H-2). – ^**13**^**C-NMR** (151 MHz, DMSO-*d*_6_): *δ* [ppm] = 59.7 (d, ^2^*J*_C,P_ = 5.2 Hz, C-1), 16.4 (d, ^3^*J*_C,P_ = 7.7 Hz, C-2). – ^**31**^**P-NMR** (243 MHz, DMSO-*d*_6_): *δ* [ppm] = -0.08. – **ATR-IR:** *ṽ* [cm^-1^] = 2981, 2905, 2253, 2128, 1688, 1479, 1450, 1393, 1164, 1100, 1050, 1024, 1006, 952, 820, 757, 621, 530, 480. – **HRMS** (ESI^-^, m/z): calcd.: 125.0009 [M-H]^-^, found: 124.9995 [M-H]^-^.

#### β-Ethyl adenosine diphosphate 28

The reaction was carried out according to general procedure 2, with 0.16 g (0.29 mmol, 1.2 eq.) AMP **39**, 5 mL CH_3_CN, 0.53 mL (2.4 mmol, 16 eq.) triethylamine and 0.33 mL (2.4 mmol, 10 eq.) TFAA, dissolved in 5 mL CH_3_CN. The residue was redissolved in 5 mL CH_3_CN and 0.33 mL (2.4 mmol, 10 eq.) triethylamine, 0.11 mL (1.4 mmol, 6.0 eq.) NMI were added. After a stirring time of 10 min, 45 mg (0.24 mmol, 1.0 eq.) of ethyl phosphate, suspended in 5 mL CH_3_CN and 2 mL DMF, was added dropwise. The reaction mixture was stirred for 8 h at room temperature and then purified by automated RP18 flash chromatography (H_2_O/CH_3_CN 0-100 % Vol., 0-50 min; flow rate 20 mL/min). The product was obtained as a colourless solid (46 mg, 0.070 mmol, 30 %) as its triethylammonium salt. ^**1**^**H-NMR** (600 MHz, DMSO-*d*_6_): *δ* [ppm] = 8.47 (S, 1H, H-8), 8.15 (s, 1H, H-2), 5.91 (d, ^3^*J*_H,H_ = 4.9 Hz, 1H, H-1′), 4.50 (t, ^3^*J*_H,H_ = 4.9 Hz, 1H, H-2′), 4.27 (t, ^3^*J*_H,H_ = 4.6 Hz, 1H, H-3′), 4.05 (q, ^3^*J*_H,H_ = 4.0 Hz, 1H, H-4′), 4.01-3.92 (m, 2H, H-5′), 3.81-3.74 (m, 2H, H-1′′), 1.10 (t, ^3^*J*_H,H_ = 7.1 Hz, H-2′′). – ^**13**^**C-NMR** (151 MHz, DMSO-*d*_6_): *δ* [ppm] = 156.0 (C.6), 152.7 (C-2), 149.5 (C-4), 139.4 (C-8), 118.8 (C-5), 87.3 (C-1′), 83.4 (d, ^3^*J*_C,P_ = 7.8 Hz, C-4′), 74.0 (C-2′), 70.0 (C-3′), 64.4 (d, ^2^*J*_C,P_ = 4.9 Hz, C-5′), 60.4 (d, ^2^*J*_C,P_ = 5.9 Hz, C-1′′), 16.3 (d, ^3^*J*_C,P_ = 7.8 Hz, C-2′′). – ^**31**^**P-NMR** (243 MHz, DMSO-*d*_6_): *δ* [ppm] = -11.82 (d, ^2^*J*_P,P_ = 19.1 Hz, P_α_), -12.23 (d, ^2^*J*_C,P_ = 19.1 Hz, P_β_). – **ATR-IR:** *ṽ* [cm^-1^] = 2991, 2634, 2486, 1671, 1476, 1400, 1361, 1316, 1196, 1170, 1121, 1064, 1038, 935, 901, 826, 798, 718, 593, 554, 517, 465, 438, 427. – **HRMS** (ESI^-^, m/z): calcd.: 454.0534 [M-H]^-^, found: 454.0451 [M-H]^-^.

### Cell culture and reagents

CaLu-3 cells (Cytion, 305032), Vero E6 cells (ATCC® CRL-1586) and A549 cells expressing ACE-2 and TMPRSS2 (A549-A/T cells, provided by Krzysztof Pyrć, Virology Laboratory at the Malopolska Centre of Biotechnology of the Jagiellonian University) were kept in high glucose DMEM with Glutamax-I (Gibco, Life Technologies), 10 % foetal calf serum (Biochrom), 100 U/l penicillin, 100 µg/l streptomycin (Invitrogen, Life Technologies), 1 mM non-essential amino acids (Gibco, Life Technologies), 1 mM pyruvate (Gibco, Life Technologies) at 37˚C in a humidified atmosphere with 5% CO_2_. HEK-293T-Ace-2-TMPRSS2 cells (BEI resources, NR-55293) were maintained as described above but with 3% foetal calf serum. A549-A/T cell identity was confirmed by STR profiling (MicroSynth). Cells were authenticated by the supplier by either STR profiling (CaLu-3) or species-specific PCR (Vero E6, HEK-293T-Ace2-TMPRSS2). All cells were routinely tested for mycoplasma contamination.

Human recombinant interferon γ (Cell Guidance Systems) was diluted in sterile water to a concentration of 1000 U/µl as recommended by the manufacturer. Remdesivir (Sigma-Aldrich, Merck) was prepared as a 50 mM stock solution in DMSO.

### Construction of bacterial expression plasmids

The expression vector pETM33_Nsp3b_ADRP for the isolated Mac1 domain of NSP3 from SARS-CoV-2, which was a gift from Ylva Ivarsson (Addgene plasmid #156468; http://n2t.net/addgene:156468; RRID:Addgene_156468)^70^ was the basis for the construction of all other constructs. Mac1 variants were created by site-directed mutagenesis PCR using the QuikChange Protocol (Agilent Technologies) according to the manufacturer’s instructions and were amplified in XL1blue *Escherichia coli* (Agilent, 200249). For Mac1 Phe132Ala the primers used were: 5′-GCTGAGCGCGGGTATCGCGGGTGCGGACCCGATTC-3′ and 5′-GAATCGGGTCCGCACCCGCGATACCCGC GCTCAGC-3′, for Mac1-mod2 the primers used were: 5′-GGTTAGCAGCTTTCTGGAAATGAAAAGC GAAAAATAAGAATTCGAGCTCC-3′ and 5′-GGAGCTCGAATTCTTATTTTTCGCTTTTCATTTCCAGAAAGCTG CTAACC-3′.

### Expression and purification of Mac1

For expression of GST-6xHis-tagged Mac1 fusion proteins *E.coli* Rosetta2 (Merck Millipore, 71402) were transformed with expression vectors for Mac1, Mac1 Phe132Ala or Mac1-mod2 by heat-shock. Bacteria were grown in ZYM-5052 auto-induction medium^71^ with 60 µg/ ml kanamycin and 34 µg/ ml chloramphenicol in a shaker (Infors HT) at 180 rpm and 37 °C over night. Cells were pelleted and resuspended in immobilized metal ion affinity chromatography (IMAC) buffer (50 mM NaH_2_PO_4_, 500 mM NaCl, 5 mM imidazole, pH 7.8), supplemented with 50 U/ ml benzonase (Sigma-Aldrich) and protease inhibitor (Roche Complete EDTA-free) prior to lysis by a French press (CF1 cell disruptor, I&L Biosystems) at 1.8 kbar. Cleared lysates were loaded on a pre-equilibrated 1 mL His-Trap FF column (Cytiva) attached to an Äkta Pure FPLC system (Cytiva). Affinity-bound proteins were eluted in a gradient from 5 to 500 mM imidazole in 10 column volumes. The GST-6xHis tag was cleaved using His-tagged 3 C protease, provided by Susanne Witt (CSSB, Hamburg, Germany), and removed together with the protease by passing through a 1 mL His-Trap FF column. Isolated Mac1 was further purified by size exclusion chromatography on a Superdex 75 Increase 10/ 300 GL column (Cytiva) in 20 mM HEPES, 500 mM NaCl and pH 7.8. Finally, the protein samples were concentrated using a centrifugal filter device (3 kDa Amicon, Sigma Aldrich) before being snap-frozen and stored at −80 °C.

### HPLC assay for enzymatic activity of Mac1, human MacroD1 and human MacroD2

Enzyme reactions were started by dilution of 10x enzyme stocks or storage buffer (enzyme control) directly in reaction buffer (20 mM KH_2_PO_4_, 4 mM MgCl_2_, 400 µM DTT, pH 7.2) supplemented with 10 µM α-NAD^+^ **7** as substrate and with or without inhibitor (vehicle control) in 1.5 ml reaction tubes (Sarstedt). The final concentrations were 3 µM for Mac1, 1 µM MacroD1, or 3 µM MacroD2. Reaction mixtures were incubated at 37 °C in a thermomixer (Eppendorf). After 2 h, samples were 5-fold diluted in ice-cold water, followed by immediate snap-freezing in liquid N_2_ and stored at −80 °C until further analysis by HPLC as described above. Enzyme inhibition was quantified by ADPR **1** formation using the following formula:

1 $${{{\rm{Inhibition}}}}\left(\%\right)=100\times \frac{{n\left({{{\rm{ADPR}}}}\right)}_{{{{\rm{vehicle\; control}}}}}-{n\left({{{\rm{ADPR}}}}\right)}_{{{{\rm{sample}}}}}}{{n\left({{{\rm{ADPR}}}}\right)}_{{{{\rm{vehicle\; control}}}}}-{n\left({{{\rm{ADPR}}}}\right)}_{{{{\rm{enzyme\; control}}}}}}$$

### Fluorimetric assay for Mac1 activity

If not stated otherwise, reaction conditions, enzyme and reagent concentrations were analogous to the HPLC assay. In brief, 10x concentrated enzyme stocks or storage buffer (enzyme control) was prepared in 96-well twin.tec semi-skirted PCR plates (Eppendorf). Reaction buffer supplemented with α-NAD^+^ **7** with or without inhibitor (vehicle control) was added to start the reactions. Plates were sealed (Sarstedt), briefly spun down and incubated at 37 °C for 2 h in a Mastercycler (Eppendorf). Enzyme activity was quantified by a post-reaction derivatisation reaction of the remaining substrate as described by Wazir and coworkers^24^.

Briefly, reaction mixtures were transferred to black 96-well polypropylene flat-bottom plates (Sarstedt) containing KOH (final ~0.6 M). Next, acetophenone was added to the samples (final ~7%) and incubated for 10 min at room temperature (RT). Immediately after that, reactions were neutralized by addition of formic acid (final: ~30%) and let stand at RT for another 30 min. Finally, the fluorescently derivatized α-NAD^+^ **7** was detected with an Infinite M plex plate reader (Tecan) using excitation at 377 nm and emission at 444 nm. Enzyme inhibition was calculated from the mean fluorescence intensity (F) of 3 technical triplicates using the formula:

2 $${{{\rm{Inhibition}}}}\,\left(\%\right)=100\times \left(1-\frac{{F}_{{{{\rm{enzyme\; control}}}}}-{F}_{{{{\rm{sample}}}}}}{{F}_{{{{\rm{enzyme\; control}}}}}-{F}_{{{{\rm{vehicle\; control}}}}}}\right)$$

### Isothermal titration calorimetry (ITC)

For determination of binding isotherms, both ligands and proteins were dissolved in binding buffer (20 mM HEPES, 150 mM NaCl, pH 7.5). Thus, 750 µM ligand (syringe) was titrated to 50 µM protein (cell) in a total of 19 injections (starting with a single injection of 0.5 µl, followed by 18 injections of 2 µl). Thermograms were recorded at 25 °C under continuous stirring at 750 rpm using a MicroCal iTC200 (Malvern Panalytical) with the reference power set to 5 µcal/ s for wildtype Mac1, whereas a PEAQ-ITC (Malvern Panalytical) was used for titration of ST161 as well as for binding studies with Mac1 Phe132Ala under the same conditions but a reference power of 10 µcal/ s.

### Crystallization and structure determination

Crystals of the Mac1/ligand complexes were grown by sitting drop vapor diffusion technique. Ligands (at 2 mM final concentration) were added to purified Mac1-mod2 (13.6 mg/ mL final concentration) before mixing 1 µL of the protein-ligand complex with 1 µL of precipitant (1.8 M K_2_HPO_4_/NaH_2_PO_4_, pH 8.2). Needle-shaped crystals appeared after one to two days and were used to generate seeds in order to ensure consistent nucleation and larger crystals formation. A dilution series (10^−1^ to 10^−7^) of the seed stock was generated and added (0.2 µL) to a fresh Mac1/ligand-precipitant mixture.

For Mac1-mod2/ β-methyl-GS-441524-DP **4** complexes, additionally a Hampton additive screen was performed (HT-27, Hampton Research) to improve a higher crystal quality. Final crystallization conditions for this complex were: 90% v/v mother solution (as described above, but without seeding) + 10 % v/v formamide (40 % v/v). Mac1-mod2 crystals co-crystallized with β-ethyl-GS-441524 phosphonate-phosphate (ST161) **42** were grown in 24-well plates using 1.8 M K_2_HPO_4_ / NaH_2_PO_4_ pH 8.3 buffer saturated with ligand (ST161 **42**) at 5 mM concentration as crystallization buffer at 19 °C. Usually, crystals grew within one day, were cryoprotected with 20 % glycerol and directly flash-frozen in liquid nitrogen for storage and data collection.

X-ray diffraction data were collected at 100 K at the PETRA III/EMBL P13 beamline^72^. Raw data were processed using the AUTOPROC^73^ pipeline for data reduction and scaling. Phases were generated by molecular replacement with PHASER^74^ using the apo Mac1 structure (PDB: 6WEN) as the search model. Iterative model building rounds with structural refinements was carried out in Coot^75^ and PHENIX.refine^76^ with a final model optimisation before deposition using PDB-REDO^77^. All data collection and structure refinement statistics are summarized in Supplementary Table 3. Structural coordinates and structural factors have been deposited in the RCSB Protein Data Bank under the accession numbers 8AZC, 8AZD, 8AZI, 8AZL, 8AZM, 8AZO, 8AZP, 9RHO, 9RHN.

### Cellular uptake assays

Approximately 40.000 CaLu-3 cells were seeded per cavity of a transparent sterile 96-well flat bottom cell culture plate (Sarstedt) and grown until ~90% confluency. Then, supernatants were removed and cells were washed with pre-warmed isotonic phosphate buffer (10 mM KH_2_PO_4_, 5.5 mM glucose, 140 mM NaCl, 1 mM MgSO_4_,5 mM KCl, pH 7.4) 3 times prior to treatment with the inhibitors (solved in isotonic phosphate buffer) or without compound (mock) at 37 °C in a cell culture incubator. At the indicated time points, cells were kept on ice and supernatants were removed and washed 3 times with ice-cold hypotonic buffer (10 mM KH_2_PO_4_, pH 7.4) prior to swelling in hypotonic buffer on ice for 5 min. Then cells were lysed by addition of a concentrated detergent solution (final: 1% Triton X100, 3 mM MgCl_2_). Cell lysates were immediately passed through a 10 kDa size exclusion filter (Pall) and stored at −80 °C until further analysis by HPLC as described above.

### Cell viability assay

For testing of cytotoxic effects by the compounds, CaLu-3 cells were treated with the compounds for 1 h as described in the uptake assay above. Next, supernatants were removed and a MTS-assay was conducted by incubating the cells or empty wells (blank) for another hour with cell culture media supplemented with CellTiter 96® AQueous One solution (Promega) according to the manufacturer recommendations. Lastly, absorbance at 490 nm (A_490_) was measured by a Tecan Infinite M plex plate reader. Cell viability was calculated by the mean A_490_ values of technical triplicates using the formula:

3 $${{{\rm{Viability}}}}\,\left(\%\right)=100\times \frac{{A}_{490{{{\rm{nm}}}},{{{\rm{sample}}}}}-{A}_{490{{{\rm{nm}}}},{{{\rm{blank}}}}}}{{A}_{490{{{\rm{nm}}}},{{{\rm{mock}}}}}-{A}_{490{{{\rm{nm}}}},{{{\rm{blank}}}}}}$$

### Construction and rescue of recombinant SARS-CoV-2

Based on a previously described cDNA clone encoding for a luciferase (NanoLuc) in orf7^78^, two type IIS restriction enzyme sites (*Bsa*I-sites) were initially deleted to allow for a golden-gate cloning strategy as described^79^. For that purpose, a total of 10 PCR fragments, including 9 fragments in the region of the viral genome plus a linker-fragment spanning the vector sequence were amplified using a high fidelity polymerase (Q5 polymerase, NEB). To generate the F132A cDNA clone, primers gg_F132A_ fwd (5´-ggctacggtctcgtggtgctgaccctatacattct and gg_F132A_rev (5´- ggctacggtctcgaccagcaataccagctgataataatg-3´) were used. Details on primers are given in Supplementary Table 4. The resulting PCR products of correct lengths were purified (AmPureXP beads, Beckman Coulter) and sequenced (Microsynth) prior to further processing. For golden gate assembly, the NEBridge Golden Gate Assembly Kit (NEB) was used according to the manufacturer’s instructions (30 cycles of 5 min 37 °C and 5 min 16 °C). The ligation product was electroporated into competent *E.coli* (TransforMax EPI300, Lucigen, EC100110). Clones with the desired cDNA sequence were used to produce and purify cDNA (Nucleobond Xtra Midi, Macherey Nagel) for subsequent rescue of recombinant viruses.

To rescue the recombinant viruses, the cDNA was transfected into HEK-293-Ace-2-TMPRSS2 cells seeded to 80% confluency in 6-well plates using a 1:3 DNA/transfection reagent ratio (FuGeneHD, Promega) diluted in reduced-serum medium (Opti-MEM, Gibco). 2–4 days after transfection, a transfer to Vero E6 cells seeded to 80–90 % confluency in 6-well plates was performed (passage 0). Cultures were evaluated for occurrence of a cytopathic effect (CPE) every 2 days. RT-qPCR^80,81^ or luciferase reporter assay^78^ were performed to verify virus growth on CPE positive Vero E6 cells.

Successfully rescued recombinant viruses were grown in T75 flasks on Vero E6 (passage 1) to generate virus stocks. Stock titres were verified by plaque assay using a 1.2% Avicel overlay^82^.

### SARS-CoV-2 infection assays

All work with infectious virus was carried out in a BSL-3 safety lab with appropriate safety equipment.

For the infection experiments, cells were seeded in 96-well plates (TPP) and infected with SARS-CoV-2 at MOI = 1. After 1 h of adsorption at 37 °C, the inhibitor was added and the cells were incubated for another 2 h at 37 °C. After that, the supernatant was removed and replaced with fresh medium, and the cells were then incubated further at 37 °C. To determine viral growth, reporter assays, RT-qPCR and/or plaque assays were performed at the indicated time points. IFNγ stock solution (Cell Guidance Systems) was diluted in medium and the cells were preincubated for 20 h prior to infection.

For transcript analysis, CaLu-3 cells were seeded in 6-well plates and either left untreated or were treated with IFNγ prior to infection (MOI = 1). If not stated otherwise, ST166 **6** was added at indicated concentration and time after adsorption as described above. 48 h after infection, cells were lysed in 1 ml of trizol reagent (Invitrogen). RNA purification was performed according to the manufacturer’s instructions. RT-qPCR for genes Hprt1, IFNβ and IFITM1 was performed using TaqMan assays (Thermo Scientific, Hs02800695_m1, Hs01077958_s1, Hs00705137_s1), the detection of viral genomic RNA used primers and probes as described^83^ with the LightCycler Multiplex RNA Virus Master (Roche) and 5 µl of the extracted RNA. Amplification, detection and analysis were performed on the LightCycler 480II (Roche).

### Quantification of cellular NAD^+^

To determine Mac1 inhibitor-dependent effects on NAD^+^ levels, CaLu-3 cells were seeded and infected with SARS-CoV-2 as described above or left uninfected. To assess IFN-dependent effects, cells were pre-stimulated with IFNγ (100 U/ ml) or left unstimulated prior to infection. Inhibitor (0.3 µM prodrug **6** or 10 nM NAMPT inhibitor FK866 ^87^as control) was added after adsorption as described above.

Cellular NAD^+^ levels were determined after 2 and 48 h using a commercially available kit (MAK460, Merck) following the manufacturers instruction with the only exception that cell extracts were heated for 30 min at 60 °C (instead of 5 min at 37 °C) for virus inactivation.

For compensation of cell number variations, NAD^+^ level were normalized to total protein amount in the extract, quantified by commercially available BCA-assay kit (Thermo Scientific) according to manufacturers instructions^84^.

### SARS CoV-2 RTC nucleotide incorporation assay

Expression and purification of proteins nsp12, 7, 8 constituting the minimal SARS-CoV-2 RTC were done as described in Sama and coworkers^85^. For the RNA elongation assays, the RTC was prepared by doing a premix1 of nsp7 and nsp8 (100 µM of each) and premix2 of nsp8 and nsp12 (100 µM of nsp8 and 33.3 µM of nsp12 (bearing the P323L mutation) both in complex buffer (25 mM HEPES, pH 8, 300 mM NaCl, 10% glycerol, 5 mM TCEP, 5 mM MgCl_2_). After incubation for 30 min at room temperature (RT), equal volumes of both premixes were mixed and this final complex was further incubated for 15 min at RT. Then the concentrations were adjusted to 2 µM nsp12 (6 µM nsp7 and 12 µM nsp8 in reaction buffer 20 mM HEPES, pH 7.5, 50 mM NaCl, 5 mM MgCl_2_). An elongation complex was then formed by adding the same volume of 2 µM fluorescently labelled primer 6Fam-GUCAUUCUCC-3′ annealed to template 5′-UAGCUUCUAUGGAGAAUGAC-3′ (Biomers.net) in reaction buffer. After 10 min incubation at 30 °C, reactions were started by adding the same volume of NTP or NA or an NTP/NA mix in reaction buffer. Final concentrations in the reactions were 0.5 µM nsp12 (1.5 µM nsp7 and 3 µM nsp8; (1:3:6)) and 0.5 µM P/T. Composition and concentration of NTP/NA are given in the figure legend for each reaction. Samples of 6 µl were taken at given time points and mixed 1:3 (v/v) with FBD (formamide, 10 mM EDTA and bromophenol blue). For the 0-time point, first 3 µl of elongation mix was added to the FBD stop solution and then 3 µl of NTP/NA mix was added. Samples were heated for 10 min at 70 °C and cooled on ice for 2 min before analysis by denaturing polyacrylamide gel electrophoresis (PAGE) using 20% acrylamide, 7 M Urea in TBE buffer at 45 W. Separated product bands were then visualized using an Amersham™ Typhoon™ Biomolecular Imager (Cytiva).

### Statistics and reproducibility

ChemStation Software (Rev. C.01.05, Agilent Technologies) was used for HPLC peak area integration. Microplate reader was operated with i-Control 2.0 (Tecan). Visualization and analysis of microplate assay as well as HPLC data (chromatograms and integrals) were performed using GraphPad Prism 9. PEAQ-ITC data were analyzed and visualized using MicroCal PEAQ-ITC software or MicroCal iTC200 analysis software (Malvern Panalytics) and Origin 2019 (OriginLab) respectively. NMR spectra were collected with TOPSPIN (v3.6.4, Bruker). Mass spectra (MS) were collected with Mass Hunter Workstation LC/MS Data Aquisition for 6200 series TOF/6500 series Q-TOF (v10.1, Agilent Technologies). NMR and MS data were analysed with MestReNova software (v14.0.1-23559, Mestrelab Research S.L.). Chemical structures were drawn using ChemSketch (2020.2.1., ACD/ Labs) or ChemDraw (Revvity Signals). Crystallography data and related molecular graphics and analyses were performed with either UCSF Chimera (1.17.3 or version X)^86^ or the PyMOL Molecular Graphics System, (v3.1.0, Schrödinger, LLC) for processing and analysis of electron densities from 2mFo-DFc maps, respectively. Real-time PCR analysis was conducted with LightCycler 480 SW software (v1.5.1, Roche). If not stated otherwise, all the data are presented as mean ± SD from at least three independent experiments. Although no formal statistical method was used to predetermine sample size, plate assay robustness was checked (Supplementary Fig. 1f). No data were excluded from the analyses. Alternating pipetting schemes were used for enzymatic compound testing to minimize sampling bias in multi-well formats. The investigators were not blinded to allocation during experiments and outcome assessment. Statistical analysis was performed using GraphPad Prism (v 9.2.0 and 10.6.1). For concentration response curves data were normalized and fitted by nonlinear regression to a sigmoidal model. For all statistical testing an α of 0.05 was adopted.

### Reporting summary

Further information on research design is available in the Nature Portfolio Reporting Summary linked to this article.

## Supplementary information

Supplementary Information

Description of Additional Supplementary Files

Supplementary Data

Reporting Summary

Transparent Peer Review file

## Source data

Source Data

## Data Availability

Source data are provided within the Source Data File. Mass spectrometry data is found in Supplementary Data. Structural coordinates have been deposited in the RCSB Protein Data Bank under accession numbers 8AZC (Mac1 apo form), 8AZD (Mac1 + ADPR **1**), 8AZL (Mac1 + 2´-deoxy-2´-F-ADPR **17**), 8AZM (Mac1 + 8-Br-ADPR **13**), 8AZI (Mac1 + 2´-deoxy-ADPR **16**), 8AZO (Mac1 + β-ethyl-ADP **28**), 8AZP (Mac1 + β-methyl-ADP **29**), 9RHO (Mac1 + β-methyl-GS-441524-diphosphate **4**), 9RHN (Mac1 + β-ethyl-phosphate-phosphonate GS-441524 **42**). Source data are provided with this paper.

## References

[1] Karras, G. I. et al. The macro domain is an ADP-ribose binding module. *EMBO J.* **24**, 1911–1920 (2005). 10.1038/sj.emboj.7600664 PMC1142602 15902274

[2] Rack, J. G. M., Perina, D. & Ahel, I. Macrodomains: structure, function, evolution, and catalytic activities. *Annu Rev. Biochem.* **85**, 431–454 (2016). 10.1146/annurev-biochem-060815-014935 26844395

[3] Jankevicius, G. et al. A family of macrodomain proteins reverses cellular mono-ADP-ribosylation. *Nat. Struct. Mol. Biol.* **20**, 508–514 (2013). 10.1038/nsmb.2523 PMC7097781 23474712

[4] Suskiewicz, M. J., Prokhorova, E., Rack, J. G. M. & Ahel, I. ADP-ribosylation from molecular mechanisms to therapeutic implications. *Cell* **186**, 4475–4495 (2023). 10.1016/j.cell.2023.08.030 PMC10789625 37832523

[5] Fehr, A. R., Jankevicius, G., Ahel, I. & Perlman, S. Viral macrodomains: unique mediators of viral replication and pathogenesis. *Trends Microbiol.* **26**, 598–610 (2018). 10.1016/j.tim.2017.11.011 PMC6003825 29268982

[6] Li, C. et al. Viral macro domains reverse protein ADP-ribosylation. *J. Virol.* **90**, 8478–8486 (2016). 10.1128/JVI.00705-16 PMC5021415 27440879

[7] Alhammad, Y. M. O. & Fehr, A. R. The viral macrodomain counters host antiviral ADP-ribosylation. *Viruses* **12**, 1–12 (2020). 10.3390/v12040384 PMC7232374 32244383

[8] Rack, J. G. M. et al. Viral macrodomains: a structural and evolutionary assessment of the pharmacological potential. *Open Biol.* **10**, 200237 (2020). 10.1098/rsob.200237 PMC7729036 33202171

[9] Zimmermann, L. et al. SARS-CoV-2 nsp3 and nsp4 are minimal constituents of a pore spanning replication organelle. *Nat. Commun.* **14**, 7894 (2023). 10.1038/s41467-023-43666-5 PMC10689437 38036567

[10] Lei, J., Kusov, Y. & Hilgenfeld, R. Nsp3 of coronaviruses: structures and functions of a large multi-domain protein. *Antivir. Res* **149**, 58–74 (2018). 10.1016/j.antiviral.2017.11.001 PMC7113668 29128390

[11] O’Connor, J. J., Ferraris, D. & Fehr, A. R. An update on the current state of SARS-CoV-2 Mac1 inhibitors. *Pathogens* **12**, 1221 (2023). 10.3390/pathogens12101221 PMC10610136 37887737

[12] Russo, L. C. et al. The SARS-CoV-2 Nsp3 macrodomain reverses PARP9/DTX3L-dependent ADP-ribosylation induced by interferon signaling. *J. Biol. Chem.* **297**, 101041 (2021). 10.1016/j.jbc.2021.101041 PMC8332738 34358560

[13] Schuller, M. et al. Fragment binding to the Nsp3 macrodomain of SARS-CoV-2 identified through crystallographic screening and computational docking. *Sci. Adv.* **7**, 1–24 (2021). 10.1126/sciadv.abf8711 PMC8046379 33853786

[14] Dasovich, M. et al. High-throughput activity assay for screening inhibitors of the SARS-CoV-2 Mac1 macrodomain. *ACS Chem. Biol.* **17**, 17–23 (2022). 10.1021/acschembio.1c00721 34904435

[15] Roy, A. et al. Discovery of compounds that inhibit SARS-CoV-2 Mac1-ADP-ribose binding by high-throughput screening. *Antivir. Res.* **203**, 105344 (2022). 10.1016/j.antiviral.2022.105344 PMC9119168 35598780

[16] Gahbauer, S. et al. Iterative computational design and crystallographic screening identifies potent inhibitors targeting the Nsp3 macrodomain of SARS-CoV-2. *Proc. Natl. Acad. Sci. USA* **120**, e2212931120 (2023). 10.1073/pnas.2212931120 PMC9926234 36598939

[17] Lee, A. A. et al. Discovery of potent SARS-CoV-2 nsp3 macrodomain inhibitors uncovers lack of translation to cellular antiviral response. 2024.08.19.608619 Preprint at 10.1101/2024.08.19.608619 (2024).

[18] Wazir, S. et al. Discovery of 2-amide-3-methylester thiophenes that target SARS-CoV-2 Mac1 and repress coronavirus replication, validating Mac1 as an antiviral target. *J. Med. Chem.* **67**, 6519–6536 (2024). 10.1021/acs.jmedchem.3c02451 PMC11144470 38592023

[19] Pfannenstiel, J. J. et al. Identification of a series of pyrrolo-pyrimidine-based SARS-CoV-2 Mac1 inhibitors that repress coronavirus replication. *mBio* **16**, e03865-24 (2025). 10.1128/mbio.03865-24 PMC12153294 40407321

[20] Suryawanshi, R. K. et al. The Mac1 ADP-ribosylhydrolase is a Therapeutic Target for SARS-CoV-2. *eLife* **14**, RP103484 (2025). 10.7554/eLife.103484 PMC12629595 41258893

[21] Ni, X. et al. Structural insights into plasticity and discovery of remdesivir metabolite GS-441524 binding in SARS-CoV-2 macrodomain. *ACS Med. Chem. Lett.* **12**, 603–609 (2021). 10.1021/acsmedchemlett.0c00684 PMC7986975 33850605

[22] Alhammad, Y. M. O. et al. The SARS-CoV-2 conserved macrodomain is a mono-ADP-ribosylhydrolase. *J. Virol.* **95**, e01969-20 (2021). 10.1128/JVI.01969-20 PMC7925111 33158944

[23] Stevens, L. A. et al. The ARH and macrodomain families of α-ADP-ribose-acceptor hydrolases catalyze α-NAD+ hydrolysis. *ACS Chem. Biol.* **14**, 2576–2584 (2019). 10.1021/acschembio.9b00429 PMC8388552 31599159

[24] Wazir, S., Maksimainen, M. M., Alanen, H. I., Galera-Prat, A. & Lehtiö, L. Activity-based screening assay for mono-ADP-ribosylhydrolases. *SLAS Discov. Adv. Life Sci. R. D.* **26**, 67–76 (2021). 10.1177/2472555220928911 32527186

[25] Suydam, I. T. & Strobel, S. A. Fluorine substituted adenosines as probes of nucleobase protonation in functional RNAs. *J. Am. Chem. Soc.* **130**, 13639–13648 (2008). 10.1021/ja803336y PMC2633102 18803382

[26] Tavale, S. S. & Sobell, H. M. Crystal and molecular structure of 8-bromoguanosine and 8-bromoadenosine, two purine nucleosides in the *syn* conformation. *J. Mol. Biol.* **48**, 109–123 (1970). 10.1016/0022-2836(70)90222-6 5448585

[27] Westhof, E., Röder, O., Croneiss, I. & Lüdemann, H.-D. Ribose Conformations in the Common Purine(ß)ribosides, in Some Antibiotic Nucleosides, and in Some Isopropylidene Derivatives: A Comparison. *Z. Naturforsch. C.* **30**, 131–140 (1975). 10.1515/znc-1975-3-401 125961

[28] Ts’o, P. O. P., Kondo, N. S. & Schweizer, M. P. Studies of the conformation and interaction in dinucleoside mono- and diphosphates by proton magnetic resonance. *Biochemistry* **8**, 997–1029 (1969). 10.1021/bi00831a033 5781031

[29] Zhang, N., Zhang, S. & Szostak, J. W. Activated ribonucleotides undergo a sugar pucker switch upon binding to a single-stranded RNA template. *J. Am. Chem. Soc.* **134**, 3691–3694 (2012). 10.1021/ja212027q PMC3448298 22296305

[30] Salmaso, V. & Jacobson, K. A. Survey of ribose ring pucker of signaling nucleosides and nucleotides. *Nucleosides Nucleotides Nucleic Acids* **39**, 322–341 (2020). 10.1080/15257770.2019.1658115 PMC7047539 31460850

[31] Ikeda, H. et al. The effect of two antipodal fluorine-induced sugar puckers on the conformation and stability of the dickerson-drew dodecamer duplex [d(CGCGAATTCGCG)]2. *Nucleic Acids Res.* **26**, 2237–2244 (1998). 10.1093/nar/26.9.2237 PMC147537 9547286

[32] Drown, B. S., Shirai, T., Rack, J. G. M., Ahel, I. & Hergenrother, P. J. Monitoring poly(ADP-ribosyl)glycohydrolase activity with a continuous fluorescent substrate. *Cell Chem. Biol.* **25**, 1562–1570.e19 (2018). 10.1016/j.chembiol.2018.09.008 PMC6309520 30318463

[33] Gollnest, T. et al. Membrane-permeable Triphosphate Prodrugs of Nucleoside Analogues. *Angew. Chem. Int. Ed. Engl.* **55**, 5255–5258 (2016). 10.1002/anie.201511808 27008042

[34] Meier, C. & Balzarini, J. Application of the *cyclo*Sal-prodrug approach for improving the biological potential of phosphorylated biomolecules. *Antivir. Res.* **71**, 282–292 (2006). 10.1016/j.antiviral.2006.04.011 16735066

[35] Jessen, H. J., Schulz, T., Balzarini, J. & Meier, C. Bioreversible protection of nucleoside diphosphates. *Angew. Chem. Int. Ed.* **47**, 8719–8722 (2008). 10.1002/anie.200803100 18833560

[36] Pahnke, K. & Meier, C. Synthesis of a bioreversibly masked lipophilic adenosine diphosphate ribose derivative. *ChemBioChem* **18**, 1616–1626 (2017). 10.1002/cbic.201700232 28589630

[37] Mohamady, S. & Taylor, S. D. General procedure for the synthesis of dinucleoside polyphosphates. *J. Org. Chem.* **76**, 6344–6349 (2011). 10.1021/jo200540e 21688809

[38] Rijpkema, K. J. et al. Synthesis of structural ADP-ribose analogues as inhibitors for SARS-CoV-2 macrodomain 1. *Org. Lett.* **26**, 5700–5704 (2024). 10.1021/acs.orglett.4c01792 PMC11249776 38935522

[39] Taha, T. Y. et al. A single inactivating amino acid change in the SARS-CoV-2 NSP3 Mac1 domain attenuates viral replication in vivo. *PLOS Pathog.* **19**, e1011614 (2023). 10.1371/journal.ppat.1011614 PMC10499221 37651466

[40] Kerr, C. M. et al. Mutation of a highly conserved isoleucine residue in loop 2 of several β-coronavirus macrodomains indicates that enhanced ADP-ribose binding is detrimental for replication. *J. Virol.* **98**, e0131324 (2024). 10.1128/jvi.01313-24 PMC11575489 39387584

[41] Elliott, T. S., Slowey, A., Ye, Y. & Conway, S. J. The use of phosphate bioisosteres in medicinal chemistry and chemical biology. *Med. Chem. Commun.* **3**, 735 (2012).

[42] Agnew-Francis, K. A. & Williams, C. M. Squaramides as bioisosteres in contemporary drug design. *Chem. Rev.* **120**, 11616–11650 (2020). 10.1021/acs.chemrev.0c00416 32930577

[43] Baszczyňski, O. et al. Synthesis of phosphonoacetate analogues of the second messenger adenosine 5′-diphosphate ribose (ADPR). *RSC Adv.* **10**, 1776–1785 (2020). 10.1039/c9ra09284f PMC6957348 31934327

[44] Eckstein, F. Phosphorothioates, essential components of therapeutic oligonucleotides. *Nucleic Acid Ther.* **24**, 374–387 (2014). 10.1089/nat.2014.0506 25353652

[45] Tsika, A. C. et al. Binding adaptation of GS-441524 Diversifies macro domains and downregulates SARS-CoV-2 de-MARylation capacity. *J. Mol. Biol.* **434**, 167720 (2022). 10.1016/j.jmb.2022.167720 PMC9284540 35839840

[46] Fu, W. et al. The search for inhibitors of macrodomains for targeting the readers and erasers of mono-ADP-ribosylation. *Drug Discov. Today* **26**, 2547–2558 (2021). 10.1016/j.drudis.2021.05.007 34023495

[47] Jia, X., Schols, D. & Meier, C. Lipophilic triphosphate prodrugs of various nucleoside analogues. *J. Med. Chem.* **63**, 6991–7007 (2020). 10.1021/acs.jmedchem.0c00358 32515595

[48] Jia, X., Ganter, B. & Meier, C. Chapter One—Improving properties of the nucleobase analogs T-705/T-1105 as potential antiviral. in *Annual Reports in Medicinal Chemistry* (ed. Seley-Radtke, K.) vol. 57 1–47 (Academic Press, 2021). 10.1016/bs.armc.2021.08.002 PMC8553380 34728864

[49] Huchting, J. et al. Prodrugs of the phosphoribosylated forms of hydroxypyrazinecarboxamide pseudobase T-705 and its de-fluoro analogue T-1105 as potent influenza virus inhibitors. *J. Med. Chem.* **61**, 6193–6210 (2018). 10.1021/acs.jmedchem.8b00617 29906392

[50] Gollnest, T., de Oliveira, T. D., Schols, D., Balzarini, J. & Meier, C. Lipophilic prodrugs of nucleoside triphosphates as biochemical probes and potential antivirals. *Nat. Commun.* **6**, 8716 (2015). 10.1038/ncomms9716 PMC4640093 26503889

[51] Meier, C., Jessen, H. J. & Balzarini, J. Nucleoside diphosphate prodrugs. *Nucleic Acids Symp. Ser.* **52**, 83–84 (2008). 10.1093/nass/nrn042 18776264

[52] Meier, C. Nucleoside diphosphate and triphosphate prodrugs—An unsolvable task?. *Antivir. Chem. Chemother.* **25**, 69–82 (2017). 10.1177/2040206617738656 PMC5890512 29096525

[53] Zhao, C., Weber, S., Schols, D., Balzarini, J. & Meier, C. Prodrugs of γ-alkyl-modified nucleoside triphosphates: improved inhibition of HIV reverse transcriptase. *Angew. Chem. Int. Ed.* **59**, 22063–22071 (2020). 10.1002/anie.202003073 PMC7756582 32379948

[54] Jia, X., Weber, S., Schols, D. & Meier, C. Membrane permeable, bioreversibly modified prodrugs of nucleoside diphosphate-γ-phosphonates. *J. Med. Chem.* **63**, 11990–12007 (2020). 10.1021/acs.jmedchem.0c01294 32991174

[55] Tao, S. et al. Comparison of anti-SARS-CoV-2 activity and intracellular metabolism of remdesivir and its parent nucleoside. *Curr. Res. Pharmacol. Drug Discov.* **2**, 100045 (2021). 10.1016/j.crphar.2021.100045 PMC8357487 34870151

[56] Kerr, C. M. et al. Mutation of a highly conserved isoleucine residue in loop 2 of several β-coronavirus macrodomains indicates that enhanced ADP-ribose binding is detrimental for replication. *J. Virol.* **98**, e01313–e01324 (2024). 10.1128/jvi.01313-24 PMC11575489 39387584

[57] Sherrill, L. M. et al. Design, synthesis and evaluation of inhibitors of the SARS-CoV-2 nsp3 macrodomain. *Bioorg. Med. Chem.* **67**, 116788 (2022). 10.1016/j.bmc.2022.116788 PMC9093066 35597097

[58] Michalska, K. et al. Crystal structures of SARS-CoV-2 ADP-ribose phosphatase: From the apo form to ligand complexes. *IUCrJ* **7**, 814–824 (2020). 10.1107/S2052252520009653 PMC7467174 32939273

[59] Correy, G. J. et al. The mechanisms of catalysis and ligand binding for the SARS-CoV-2 NSP3 macrodomain from neutron and X-ray diffraction at room temperature. *Sci. Adv.* **8**, eabo5083 (2022). 10.1126/sciadv.abo5083 PMC9140965 35622909

[60] Kumler, W. D. & Eiler, J. J. The acid strength of mono and diesters of phosphoric acid. The n-alkyl esters from methyl to butyl, the esters of biological importance, and the natural guanidine phosphoric acids. *J. Am. Chem. Soc.* **65**, 2355–2361 (1943).

[61] Engel, R. Phosphonates as analogues of natural phosphates. *Chem. Rev.* **77**, 349–367 (1977).

[62] Rasmussen, H. B. et al. Cellular uptake and intracellular phosphorylation of GS-441524: implications for its effectiveness against COVID-19. *Viruses* **13**, 1369 (2021). 10.3390/v13071369 PMC8310262 34372575

[63] Vatandaslar, H. A systematic study on the optimal nucleotide analogue concentration and rate limiting nucleotide of the SARS-CoV-2 RNA-dependent RNA polymerase. *Int. J. Mol. Sci.* **23**, 8302 (2022). 10.3390/ijms23158302 PMC9369030 35955442

[64] Alhammad, Y. M. et al. SARS-CoV-2 Mac1 is required for IFN antagonism and efficient virus replication in cell culture and in mice. *Proc. Natl. Acad. Sci. USA* **120**, e2302083120 (2023). 10.1073/pnas.2302083120 PMC10468617 37607224

[65] Zhu, J. et al. The potential protective role of GS-441524, a metabolite of the prodrug remdesivir, in vaccine breakthrough SARS-CoV-2 infections. *Intensive Care Res.* **2**, 49–60 (2022). 10.1007/s44231-022-00021-4 PMC9645326 36407474

[66] Moreau, C. et al. Structure-activity relationship of adenosine 5′-diphosphoribose at the transient receptor potential melastatin 2 (TRPM2) channel: rational design of antagonists. *J. Med. Chem.* **56**, 10079–10102 (2013). 10.1021/jm401497a PMC3873810 24304219

[67] Fliegert, R. et al. Ligand-induced activation of human TRPM2 requires the terminal ribose of ADPR and involves Arg1433 and Tyr1349. *Biochem. J.* **474**, 2159–2175 (2017). 10.1042/BCJ20170091 PMC5473349 28515263

[68] Baszczyňski, O. et al. Synthesis of terminal ribose analogues of adenosine 5′-diphosphate ribose as probes for the transient receptor potential cation channel TRPM2. *J. Org. Chem.* **84**, 6143–6157 (2019). 10.1021/acs.joc.9b00338 PMC6528165 30978018

[69] Weising, S., Sterrenberg, V., Schols, D. & Meier, C. Synthesis and antiviral evaluation of TriPPPro-AbacavirTP, TriPPPro-CarbovirTP, and Their 1′,2′-cis-disubstituted analogues. *ChemMedChem* **13**, 1771–1778 (2018). 10.1002/cmdc.201800361 29943432

[70] Mihalič, F. et al. Identification of motif-based interactions between SARS-CoV-2 protein domains and human peptide ligands pinpoint antiviral targets. *Nat. Commun.* **14**, 5636 (2023). 10.1038/s41467-023-41312-8 PMC10499821 37704626

[71] Studier, F. W. Protein production by auto-induction in high density shaking cultures. *Protein Expr. Purif.* **41**, 207–234 (2005). 10.1016/j.pep.2005.01.016 15915565

[72] Cianci, M. et al. P13, the EMBL macromolecular crystallography beamline at the low-emittance PETRA III ring for high- and low-energy phasing with variable beam focusing. *J. Synchrotron Rad.* **24**, 323–332 (2017). 10.1107/S1600577516016465 PMC5182027 28009574

[73] Vonrhein, C. et al. Data processing and analysis with the autoPROC toolbox. *Acta Cryst. D.* **67**, 293–302 (2011). 10.1107/S0907444911007773 PMC3069744 21460447

[74] McCoy, A. J. Solving structures of protein complexes by molecular replacement with Phaser. *Acta Cryst. D.* **63**, 32–41 (2007). 10.1107/S0907444906045975 PMC2483468 17164524

[75] Emsley, P., Lohkamp, B., Scott, W. G. & Cowtan, K. Features and development of Coot. *Acta Cryst. D.* **66**, 486–501 (2010). 10.1107/S0907444910007493 PMC2852313 20383002

[76] Afonine, P. V. et al. Towards automated crystallographic structure refinement with phenix.refine. *Acta Cryst. D.* **68**, 352–367 (2012). 10.1107/S0907444912001308 PMC3322595 22505256

[77] Joosten, R. P., Long, F., Murshudov, G. N. & Perrakis, A. The PDB_REDO server for macromolecular structure model optimization. *IUCrJ* **1**, 213–220 (2014). 10.1107/S2052252514009324 PMC4107921 25075342

[78] Rihn, S. J. et al. A plasmid DNA-launched SARS-CoV-2 reverse genetics system and coronavirus toolkit for COVID-19 research. *PLoS Biol.* **19**, e3001091 (2021). 10.1371/journal.pbio.3001091 PMC7906417 33630831

[79] Taha, T. Y. et al. Enhanced RNA replication and pathogenesis in recent SARS-CoV-2 variants harboring the L260F mutation in NSP6. *PLoS Pathog.* **21**, e1013020 (2025). 10.1371/journal.ppat.1013020 PMC11981139 40163530

[80] Pfefferle, S., Reucher, S., Nörz, D. & Lütgehetmann, M. Evaluation of a quantitative RT-PCR assay for the detection of the emerging coronavirus SARS-CoV-2 using a high throughput system. *Eurosurveillance* **25**, 2000152 (2020). 10.2807/1560-7917.ES.2020.25.9.2000152 PMC7068162 32156329

[81] Tang, H. T. et al. Analytical and clinical validation of a novel, laboratory-developed, modular multiplex-PCR panel for fully automated high-throughput detection of 16 respiratory viruses. *J. Clin. Virol.* **173**, 105693 (2024). 10.1016/j.jcv.2024.105693 38820916

[82] Herzog, P., Drosten, C. & Müller, M. A. Plaque assay for human coronavirus NL63 using human colon carcinoma cells. *Virol. J.* **5**, 138 (2008). 10.1186/1743-422X-5-138 PMC2603006 19014487

[83] Corman, V. M. et al. Detection of 2019 novel coronavirus (2019-nCoV) by real-time RT-PCR. *Eurosurveillance* **25**, 2000045 (2020). 10.2807/1560-7917.ES.2020.25.3.2000045 PMC6988269 31992387

[84] Hasmann, M. & Schemainda, I. FK866, a highly specific noncompetitive inhibitor of nicotinamide phosphoribosyltransferase, represents a novel mechanism for induction of tumor cell apoptosis. *Cancer Res.* **63**, 7436–7442 (2003). 14612543

[85] Sama, B. et al. The effects of Remdesivir’s functional groups on its antiviral potency and resistance against the SARS-CoV-2 polymerase. *Antivir. Res.* **232**, 106034 (2024). 10.1016/j.antiviral.2024.106034 39510431

[86] Pettersen, E. F. et al. UCSF Chimera—a visualization system for exploratory research and analysis. *J. Comput. Chem.* **25**, 1605–1612 (2004). 10.1002/jcc.20084 15264254

