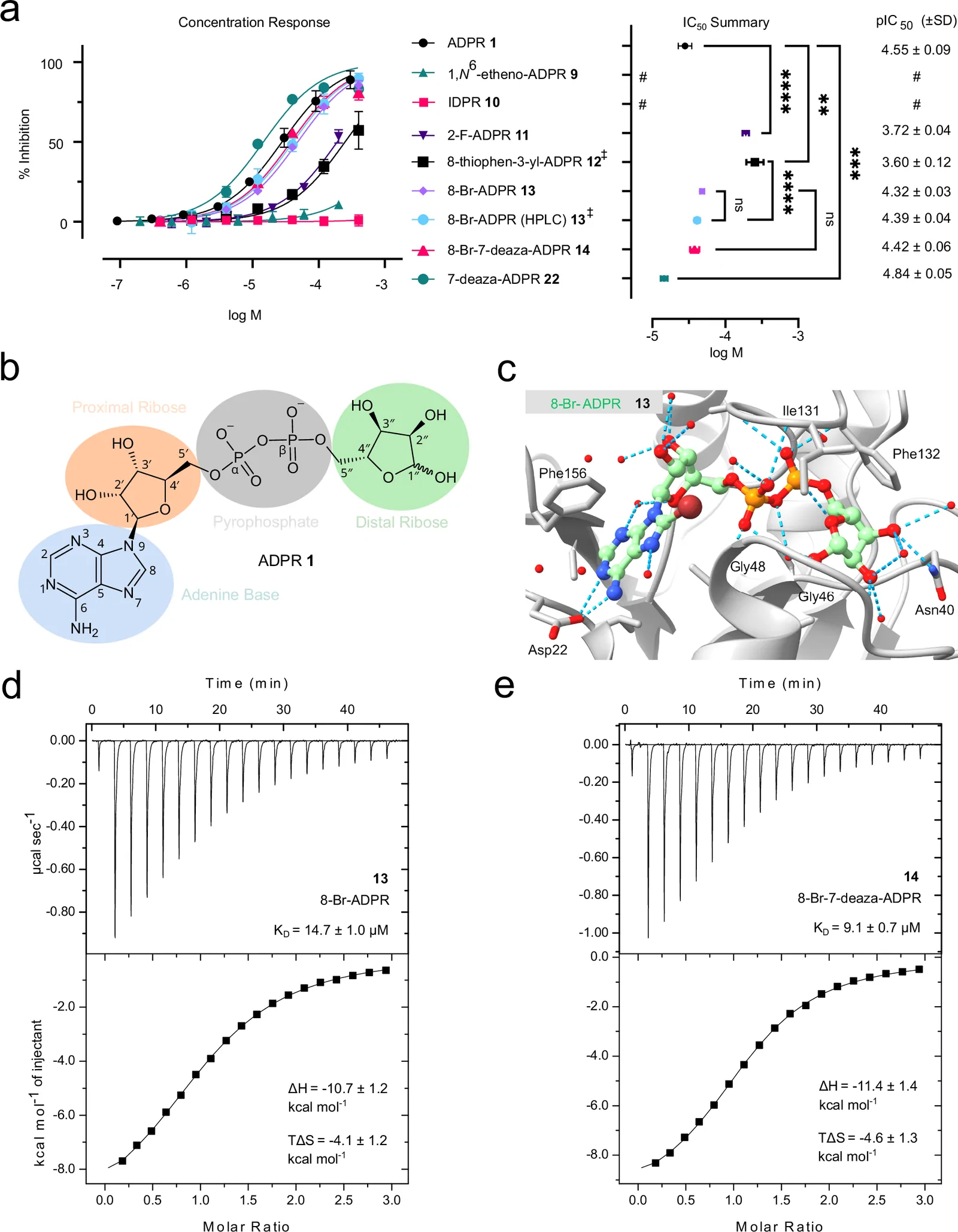# Publisher Correction: Design, structure-based optimization and antiviral evaluation of potent inhibitors for the macrodomain Mac1 of SARS-CoV-2

**DOI:** 10.1038/s41467-026-77726-3

**Published:** 2026-09-15

**Authors:** Maximilian Sandmann, Sahra Tajdar, Simon Sander, David Ruiz Carrillo, Benedikt Ganter, Celine Fischer, Marina Ocenas, Stefanie Etzold, Neele Pekarek, Julia Berger, Toni Luise Meister, Barbara Selisko, Bruno Canard, Joanna M. Watt, Ondřej Baszczyňski, Barry VL Potter, Maria Garcia Alai, Tidow Henning, Pfefferle Susanne, Chris Meier, Ralf Fliegert

**Affiliations:** 1 Department of Biochemistry and Molecular Cell Biology, University Medical Center Hamburg-Eppendorf, Hamburg, Germany; 2 Organic Chemistry, Department of Chemistry, University of Hamburg, Hamburg, Germany; 3 The Hamburg Advanced Research Center for Bioorganic Chemistry (HARBOR) & Department of Chemistry, Institute for Biochemistry and Molecular Biology, University of Hamburg, Hamburg, Germany; 4 European Molecular Biology Laboratory Hamburg, Hamburg, Germany; 5 Institute for Medical Microbiology, Virology and Hygiene, University Medical Center Hamburg-Eppendorf, Hamburg, Germany; 6 Bernhard Nocht Institute, Leibniz Institute for Tropical Medicine, Hamburg, Germany; 7 Institute for Infection Research and Vaccine Development (IIRVD), Centre for Internal Medicine, University Medical Centre Hamburg-Eppendorf (UKE), Hamburg, Germany; 8 German Centre for Infection Research (DZIF), Partner site Hamburg-Lübeck-Borstel-Riems, Hamburg, Germany; 9 Laboratoire Architecture et Fonction des Macromolécules Biologiques (AFMB), CNRS, Aix-Marseille Université, UMR7257, Marseille, France; 10 Department of Life Sciences, University of Bath, Bath, UK; 11 Medicinal Chemistry & Drug Discovery, Department of Pharmacology, University of Oxford, Oxford, UK; 12 Centre for Structural Systems Biology (CSSB), DESY Campus, Hamburg, Germany

**Keywords:** Enzymes, Drug discovery, SARS-CoV-2, X-ray crystallography, Molecular conformation

Correction to: *Nature Communications* https://doi.org/10.1038/s41467-026-75835-7, published online 27 July 2026

In this article, the single line connecting the “1” and “2” labels in the Figure 1 (ADPR) structure was missing. The figure should have appeared as shown below. The original article has been corrected.

**Incorrect figure 1**

**Figure:**
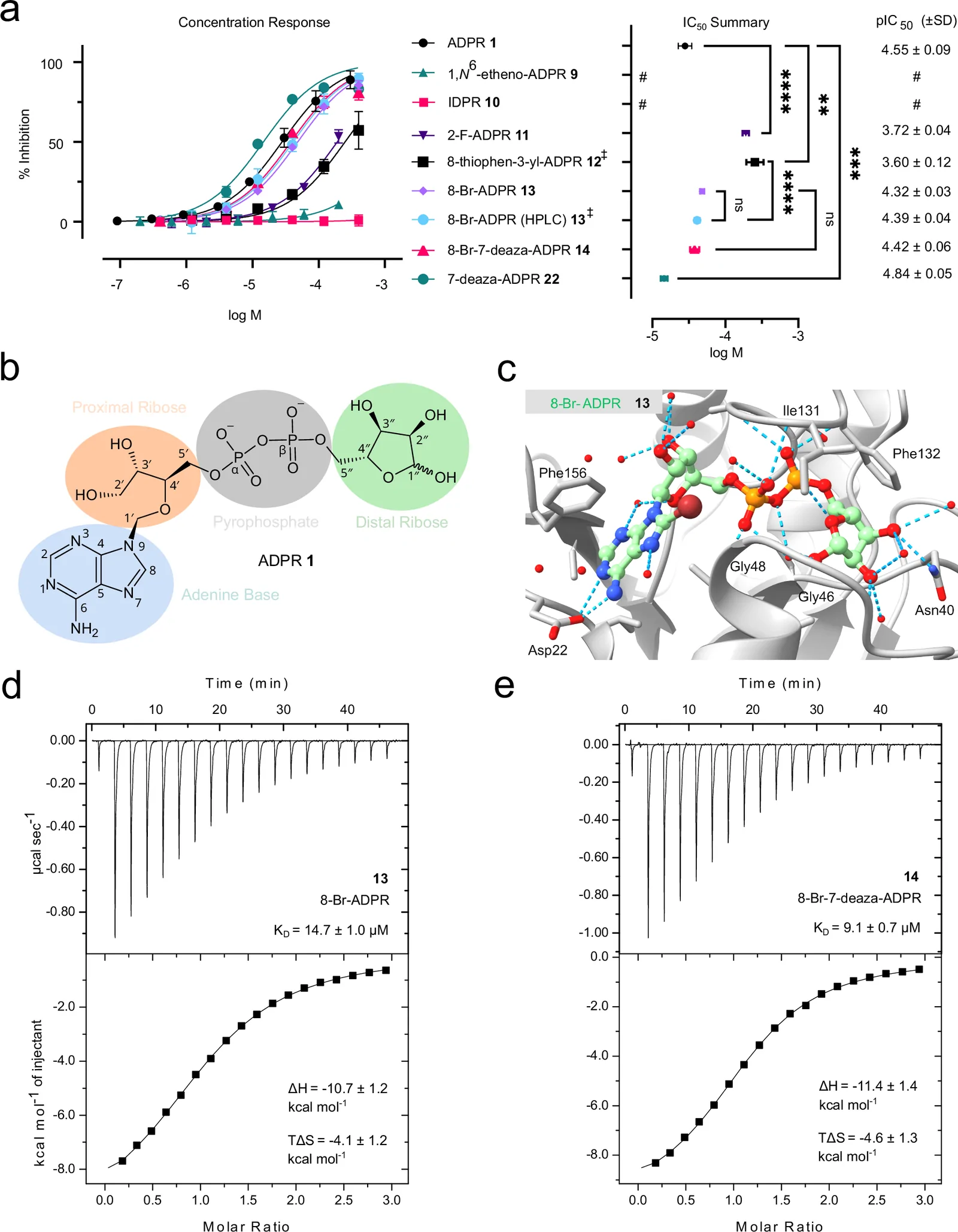

**Correct figure 1**

**Figure:**