# Publisher Correction: Design, structure-based optimization and antiviral evaluation of potent inhibitors for the macrodomain Mac1 of SARS-CoV-2

**DOI:** 10.1038/s41467-026-78176-7

**Published:** 2026-09-25

**Authors:** Maximilian Sandmann, Sahra Tajdar, Simon Sander, David Ruiz Carrillo, Benedikt Ganter, Celine Fischer, Marina Ocenas, Stefanie Etzold, Neele Pekarek, Julia Berger, Toni Luise Meister, Barbara Selisko, Bruno Canard, Joanna M. Watt, Ondřej Baszczyňski, Barry VL Potter, Maria Garcia Alai, Henning Tidow, Susanne Pfefferle, Chris Meier, Ralf Fliegert

**Affiliations:** 1 Department of Biochemistry and Molecular Cell Biology, University Medical Center Hamburg-Eppendorf, Hamburg, Germany; 2 Organic Chemistry, Department of Chemistry, University of Hamburg, Hamburg, Germany; 3 The Hamburg Advanced Research Center for Bioorganic Chemistry (HARBOR) & Department of Chemistry, Institute for Biochemistry and Molecular Biology, University of Hamburg, Hamburg, Germany; 4 European Molecular Biology Laboratory Hamburg, Hamburg, Germany; 5 Institute for Medical Microbiology, Virology and Hygiene, University Medical Center Hamburg-Eppendorf, Hamburg, Germany; 6 Bernhard Nocht Institute, Leibniz Institute for Tropical Medicine, Hamburg, Germany; 7 Institute for Infection Research and Vaccine Development (IIRVD), Centre for Internal Medicine, University Medical Centre Hamburg-Eppendorf (UKE), Hamburg, Germany; 8 German Centre for Infection Research (DZIF), Partner site Hamburg-Lübeck-Borstel-Riems, Hamburg, Germany; 9 Laboratoire Architecture et Fonction des Macromolécules Biologiques (AFMB), CNRS, Aix-Marseille Université, UMR7257, Marseille, France; 10 Department of Life Sciences, University of Bath, Bath, UK; 11 Medicinal Chemistry & Drug Discovery, Department of Pharmacology, University of Oxford, Oxford, UK; 12 Centre for Structural Systems Biology (CSSB), DESY Campus, Hamburg, Germany

**Keywords:** Enzymes, Drug discovery, SARS-CoV-2, X-ray crystallography, Molecular conformation

Correction to: *Nature Communications* https://doi.org/10.1038/s41467-026-75835-7, published online 27 July 2026

In the version of this article initially published, the first and last names of Henning Tidow and Susanne Pfefferle were both interchanged, while in Fig. 1b, a bond was missing in the lower-left section of the structure shown for proximal ribose in ADPR 1. The figure is now amended in the HTML and PDF versions of the article.

