# Supplementary material for: Design, structure-based optimization and antiviral evaluation of potent inhibitors for the macrodomain Mac1 of SARS-CoV-2
Source: Nat Commun. 2026 Jul 27;17:7416. doi: 10.1038/s41467-026-75835-7 (PMC13408504; doi:10.1038/s41467-026-75835-7)
Supplement: Supplementary file 1 — Supplementary Information [file 41467_2026_75835_MOESM1_ESM.pdf]

Supplementary Table 1: Compound ID in this study.

ID	Compound (full name)	Abbreviation(s)
1	Adenosine 5′-*O*-diphosphoribose	ADP-ribose, ADPR
2	β-Nicotinamide adenine dinucleotide	β-NAD^+
3	GS-441524	GS-441524
4	β-methyl-GS-441524 diphosphate	β-methyl-GS-441524 DP
5	C_{11}-acyloxybenzyl-β-methyl-GS-441524 diphosphate	ST135, C_{11}-AB-β-methyl-GS-441524 DP
6	β-(Dodecanoyloxybenzyl)-(ethyl)-GS-441524 phosphate phosphonate	ST166, C_{11}-AB-β-ethyl-phosphonate-phosphate-GS-441524
7	α-Nicotinamide adenine dinucleotide	α-NAD^+
8	Nicotinamide	Nicotinamide, NicAmide
9	1,N^6-ethenoadenosine-5′-*O*-diphosphoribose	1,N^6-etheno-ADPR
10	Inosine-5′-*O*-diphosphoribose	IDPR
11	2-Fluoroadenosine 5′-*O*-diphosphoribose	2F-ADPR
12	8-Thiophen-3-yl-adenosine 5′-*O*-diphosphoribose	8-thiophen-3-yl-ADPR
13	8-Bromoadenosine 5′-*O*-diphosphoribose	8-Br-ADPR
14	8-Bromo-7-deazaadenosine 5′-*O*-diphosphoribose	8-Br-7-deaza-ADPR
15	2′-Phosphoadenosine 5′-*O*-diphosphoribose	ADPR2′-phosphate, ADPRP
16	2′-Deoxyadenosine 5′-*O*-diphosphoribose	2′-deoxy-ADPR
17	2′-Fluoro-2′-deoxyadenosine 5′-*O*-diphosphoribose	2′-deoxy-2′-F-ADPR
18	Adenosine 5′-*O*-diphosphate	ADP
19	2′-Deoxyadenosine 5′-*O*-diphosphate	2′-deoxy-ADP
20	7-Deazaadenosine 5′-*O*-diphosphate	7-deaza-ADP
21	7-Deaza-2′-deoxyadenosine 5′-*O*-diphosphate	7-deaza-2′-deoxy-ADP
22	7-Deazaadenosine diphosphate ribose	7-deaza-ADPR
23	Adenosine 5′-*O*-diphosphoglucose	ADP-glucose
24	2′-3″-Dideoxyadenosine 5′-*O*-diphosphoribose	2′,3″-dideoxy-ADPR
25	1″,2′-Dideoxyadenosine 5′-*O*-diphosphoribose	1″,2′-dideoxy-ADPR
26	β Tetrahydrafuranyl-adenosine 5′-*O*-diphosphate	THF-ADP
27	β Cyclopentyl-adenosine 5′-*O*-diphosphate	Cyclopentyl-ADP
28	β-Ethyl-adenosine 5′-*O*-diphosphate	β-ethyl-ADP
29	β-Methyl-adenosine 5′-*O*-diphosphate	β-methyl-ADP
30	5-ribosyl-squaryl-adenosine	/
31	Adenosine-5′-*O*-(2-phosphoryl)acetate ribose	A-acetyl-PR
32	Adenosine-5′-phosphonoacetyl-ribose	AMP-acetyl-R
33	α-β methylene-adenosine 5′-*O*-diphosphoribose	CH_2-ADPR, AMPcPR
34	α-β methylene-adenosine 5′-*O*-diphosphate	CH_2-ADP, AMPcP
35	Adenosine-5'-*O*-(2-thiodiphosphate)	ADP-β-S
36	(*R*p)-adenosine 5'-(1-thiodiphosphate)	(*R*p)-ADP-α-S
37	(*S*p)-adenosine 5'-(1-thiodiphosphate)	(*S*p)-ADP-α-S
38	Adenosine	/
39	Adenosine 5′-*O*-monophosphate	AMP
40	GS-441524-monophosphate	GS-441524 MP

41	GS-441524-diphosphate	GS-441524 DP
42	β-ethyl-phosphonate-phosphate-GS-441524	ST161
43	GS-441524 triphosphate	GS-441524-TP
44	β-Nicotinamide mononucleotide	β-NMN
45	4-(hydroxymethyl)phenyldodecanoate	/
46	Triethylammonium ethylphosphonate	/
47	Triethylammonium (4-dodecanoyloxybenzyl)-ethyl phosphonate	/

Supplementary Table 2: Summary of IC_{50} and K_D values.

Figure	ID	Compound	Macro-domain	activity assay		binding assay	
				type[§]	IC_{50} (µM)	type*	K_D (µM)
1	1	ADPR	Mac1 WT	P	28	/	/
	10	IDPR	Mac1 WT	P	>400	/	/
	9	1,*N*[6]-etheno-ADPR	Mac1 WT	P	>200	/	/
	11	2-F-ADPR	Mac1 WT	P	191	/	/
	13	8-Br-ADPR	Mac1 WT	P	48	M	14.7
				H	41		
	12	8-thiophen-3-yl-ADPR	Mac1 WT	H	254	/	/
	14	8-Br-7-deaza-ADPR	Mac1 WT	P	38	M	9
	22	7-deaza-ADPR	Mac1 WT	P	15	/	/
2	1	ADPR	Mac1 WT	P	25	/	/
	15	ADPRP	Mac1 WT	P	37	M	35.2
	16	2´-deoxy-ADPR	Mac1 WT	P	40	M	12.6
	17	2´-deoxy-2´-F-ADPR	Mac1 WT	P	49	M	20.3
3	1	ADPR	Mac1 WT	P	24	/	/
	23	ADP-glucose	Mac1 WT	P	>120	/	/
	16	2´-deoxy-ADPR	Mac1 WT	P	40	/	/
	25	1″-,2´-dideoxy-ADPR	Mac1 WT	P	149	/	/
	24	2´,3″-dideoxy-ADPR	Mac1 WT	P	111	/	/
	26	THF-ADP	Mac1 WT	P	260	/	/
	27	Cyclopentyl-ADP	Mac1 WT	P	1614	/	/
	29	β-methyl-ADP	Mac1 WT	P	13	M	6.7
	28	β-ethyl-ADP	Mac1 WT	P	40	M	19.9
5a	1	ADPR	Mac1 WT	P	28	/	/
	39	AMP	Mac1 WT	P	>2000	/	/
	38	Adenosine	Mac1 WT	P	>400	/	/
	3	GS-441524	Mac1 WT	P	73	/	/
	40	GS-441524 MP	Mac1 WT	P	44	/	/
	41	GS-441524 DP	Mac1 WT	P	26	M	10.91
5b-f, 7d	1	ADPR	Mac1 WT	P	23	/	/
	13	8-Br-ADPR	MacroD1	P	19	/	/
			MacroD2		32	/	/
	4	β-methyl-GS-441524 DP	Mac1 WT	P	0.24	M	0.17
			MacroD1		>240	/	/
			MacroD2		>240	/	/
	42	ST161	Mac1 WT	P	1.54	Q	0.86
7[$]	1	ADPR	Mac1 WT	P	22	/	/
	18	ADP	Mac1 WT	P	3656	/	/
	20	7-deaza-ADP	Mac1 WT	P	1208	/	/
	19	2´-deoxy-ADP	Mac1 WT	P	5834	/	/
	21	7-deaza-2´-deoxy-ADP	Mac1 WT	P	2667	/	/
9[$]	1	ADPR	Mac1 Phe132Ala	/	/	Q	4.9

	29	β-methyl-ADP	Mac1 Phe132Ala	/	/	Q	3.3
	28	β-ethyl-ADP	Mac1 Phe132Ala	/	/	Q	2.9
10$	1	ADPR	Mac1 WT	P	22	/	/
	13	8-Br-ADPR	Mac1 WT	H	45	/	/
	30	5-Ribosyl-squaryl-adenosine	Mac1 WT	H	>400	/	/
	32	AMP-acetyl-R	Mac1 WT	H	>400	/	/
	31	A-acetyl-PR	Mac1 WT	H	>400	/	/
	33	AMPcPR	Mac1 WT	H	>300	/	/
	18	ADP	Mac1 WT	P	3828	/	/
	34	AMPcP	Mac1 WT	P	>4000	/	/
	35	ADP-β-S	Mac1 WT	P	3926	/	/
	36	(*R*p)-ADP-α-S	Mac1 WT	P	2576	/	/
	37	(*S*p)-ADP-α-S	Mac1 WT	P	1770	/	/
17$	4	β-methyl-GS-441524-DP	Mac1 Phe132Ala			Q	0.03
	42	ST161	Mac1 Phe132Ala			Q	0.54

$Supplementary Fig.
§P = plate, H = HPLC

*M = MicroCal iTC200, Q = PEAQ ITC

Supplementary Table 3: Crystal data collection and structure refinement statistics.

	apo	ADPR **1**	2´-deoxy-ADPR **16**	2´F-2´deoxy-ADPR **17**	8-Br-ADPR **13**	β-ethyl-ADP **28**	β-methyl-ADP **29**	β-methyl-GS-441524-DP **4**	ST161 **42**
PDB code	8AZC	8AZD	8AZI	8AZL	8AZM	8AZO	8AZP	9RHO	9RHN
Wave-length	0.799	0.976	0.976	0.976	0.976	0.976	0.976	0.976	0.976
Resolution range	26.47 - 0.93 (0.96 - 0.93)	48.35 - 2.0 (2.07 - 2.0)	41.38 - 1.9 (1.97 - 1.9)	48.14 - 2.2 (2.28 - 2.2)	46.51 - 2.1 (2.18 - 2.1)	40.77 - 1.9 (1.97 - 1.9)	48.35 - 1.6 (1.66 - 1.6)	35.85 - 1.6 (1.66 - 1.6)	36.16 - 1.95 (2.02 - 1.95)
Space group	C 1 2 1	P 21 21 21	P 21 21 21	P 21 21 21	P 21 21 21	P 21 21 21	P 21 21 21	P 31	P 31
Unit cell	129.8 30.2 39.5 90 96.8 90	59.5 76.0 82.9 90 90 90	59.4 74.8 82.8 90 90 90	59.3 74.6 82.6 90 90 90	59.4 74.8 82.8 90 90 90	59.6 75.6 82.9 90 90 90	59.5 75.1 82.8 90 90 90	82.8 82.8 41.3 90 90 120	83.5 83.5 41.4 90 90 120
Total reflections	200707 (16965)	864677 (25929)	383124 (38782)	246162 (24483)	284246 (26031)	324542 (23783)	646750 (57685)	429669 (45349)	460999 (46702)
Unique reflections	100821 (8769)	26042 (2553)	29698 (2925)	19152 (1888)	22133 (2185)	29803 (2892)	49126 (4759)	41759 (4194)	23526 (2344)
Multiplicity	2.0 (1.9)	33.1 (13.6)	12.9 (13.3)	12.9 (13.0)	12.8 (11.9)	10.9 (8.2)	13.2 (12.1)	10.3 (10.8)	19.6 (19.9)
Complete-ness (%)	98.57 (86.12)	99.39 (99.18)	99.25 (99.22)	99.11 (99.00)	99.52 (99.54)	98.34 (97.50)	98.64 (97.38)	99.99 (100.00)	99.85 (100.00)
Mean I/sigma(I)	15.51 (3.37)	9.2 (1.7)	9.84 (1.71)	6.48 (1.51)	9.70 (1.92)	8.52 (1.97)	17.03 (3.17)	24.86 (7.78)	17.02 (3.26)
Wilson B-factor	6.39	27.71	29.00	34.24	30.54	23.30	19.62	18.21	20.86
R-merge	0.024 (0.177)	1.02 (1.44)	0.13 (1.45)	0.23 (1.38)	0.196 (1.46)	0.164 (1.17)	0.074 (0.803)	0.066 (0.277)	0.193 (1.086)
R-meas	0.034 (0.250)	1.044 (1.554)	0.141 (1.51)	0.237 (1.44)	0.204 (1.525)	0.172 (1.249)	0.077 (0.838)	0.069 (0.290)	0.198 (1.115)
R-pim	0.023 (0.177)	0.233 (0.581)	0.0389 (0.413)	0.066 (0.395)	0.057 (0.439)	0.0498 (0.421)	0.021 (0.238)	0.022 (0.088)	0.045 (0.249)
CC1/2	0.999 (0.917)	0.983 (0.915)	0.999 (0.899)	0.998 (0.933)	0.998 (0.871)	0.997 (0.828)	0.999 (0.948)	0.999 (0.97)	0.998 (0.869)
Reflections used in refinement	100821 (8767)	26042 (2534)	29698 (2908)	19152 (1872)	22133 (2178)	29803 (2888)	49126 (4749)	41756 (4194)	23522 (2344)
Reflections used for R-free	5037 (438)	1208 (105)	1437 (140)	918 (88)	1111 (97)	1447 (134)	2394 (225)	2023 (197)	1107 (114)
R-work	0.141 (0.172)	0.206 (0.315)	0.216 (0.376)	0.216 (0.299)	0.219 (0.324)	0.189 (0.265)	0.183 (0.239)	0.171 (0.145)	0.185 (0.252)
R-free	0.151 (0.180)	0.250 (0.373)	0.258 (0.399)	0.264 (0.409)	0.279 (0.345)	0.232 (0.311)	0.206 (0.281)	0.196 (0.183)	0.215 (0.277)
Number of non-hydrogen atoms	1633	2893	2832	2774	2848	3001	2999	3005	2973
Macro-molecules	1307	2608	2608	2608	2599	2599	2608	2579	2579

ligands	28	72	70	72	74	58	56	60	60
solvent	312	213	154	94	175	344	335	366	334
Protein residues	169	344	344	344	343	343	344	338	338
RMS (bonds)	0.008	0.007	0.023	0.007	0.007	0.167	0.027	0.008	0.008
RMS (angles)	1.50	0.83	0.95	0.95	0.89	3.22	0.87	1.18	1.11
Ramachan-dran favored (%)	98.80	97.65	97.35	98.82	97.35	97.35	98.53	99.40	99.10
Ramachan-dran allowed (%)	1.20	2.35	2.65	0.88	2.65	2.65	1.47	0.60	0.90
Ramachan-dran outliers (%)	0.00	0.00	0.00	0.29	0.00	0.00	0.00	0.00	0.00
Rotamer outliers (%)	0.00	0.00	0.00	0.00	0.00	0.00	0.00	0.35	0.00
Clashscore	3.40	8.10	16.78	14.89	14.36	10.04	4.91	1.91	2.10
Average B-factor	10.23	31.66	36.24	39.48	36.74	26.24	24.54	24.31	25.18
Macro-molecules	8.60	31.18	36.01	39.50	36.34	25.29	23.38	23.10	24.81
ligands	10.50	29.02	34.37	37.88	39.60	21.86	19.30	15.01	18.64
solvent	17.08	38.38	40.94	40.37	41.33	34.14	34.46	34.33	29.19

Supplementary Table 4: Primers for construction of recombinant SARS-CoV-2.

Primer name	sequence (5´-3´)	fragment
gg_1_fwd	GGCTACGGTCTCGTCCCAGGTAACAAACCAACCAACTTTCG	golden gate 1
gg_1_rev	GGCTACGGTCTCCCCACAACACAGGCGAACTC	golden gate 1
gg_2_fwd	GGCTACGGTCTCCGTGGCAGATGCTGTCATAAAAAC	golden gate 2
gg_2_rev	GGCTACGGTCTCCTCTCCTACAACTTCGGTAG	golden gate 2
gg_3_fwd	GGCTACGGTCTCCGAGACATTATACTTAAACCAGC	golden gate 3
gg_3_rev	GGCTACGGTCTCCGCCATTTTTCTAAAACCAC	golden gate 3
gg_4_fwd	GGCTACGGTCTCCTGGCATTCCCATCTGGTAAAG	golden gate 4
gg_4_rev	GGCTACGGTCTCCAAAGTAAGAATCAATTAAATTGTCATCTTCG	golden gate 4
gg_5_fwd	GGCTACGGTCTCCCTTTGTAGTTAAGAGACACAC	golden gate 5
BsaI del orf 1 rev	GGCTACGGTCTCGCCTATCAGACATTATGCAAAGTAT	golden gate 5
BsaI del orf 1fwd	GGCTACGGTCTCGTAGGGACCTTTATGACAAGTTGCA	golden gate 6
gg_6_rev	GGCTACGGTCTCGTAATGTGTTTAAATATTGACACAG	golden gate 6
gg_7_fwd	GGCTACGGTCTCGATTAACATTAGCTGTACCCTATAATATG	golden gate 7
Bsa del spike rev	GGCTACGGTCTCGGTCCCTAGCAGCAATATCACCAAGGCA	golden gate 7
Bsa del spike fwd	GGCTACGGTCTCGGGACCTCATTTGTGCACAAAAGT	golden gate 8
gg_8_rev	GGCTACGGTCTCCCGGGATGATGACATGGATG	golden gate 8
gg_9_fwd	GGCTACGGTCTCCCCCGTATGAAGGTCTGAG	golden gate 9
gg_9_rev	GGCTACGGTCTCGAAGCTATTAAAATCACATGGGGATAGCACTA	golden gate 9
link gg fwd	GGCTACGGTCTCGGCTTCTTAGGAGAATGACAAAAAAAA	golden gate linker
link gg rev	GGCTACGGTCTCGGGGAAGGTATAAACCTTTAATACGGTTCACTAAACCAGCTCT	golden gate linker
gg_F132A_ fwd	GGCTACGGTCTCGTGGTGCTGACCCTATACATTCT	golden gate F132A
gg_F1532A_rev	GGCTACGGTCTCGACCAGCAATACCAGCTGATAATAATG	golden gate F132A

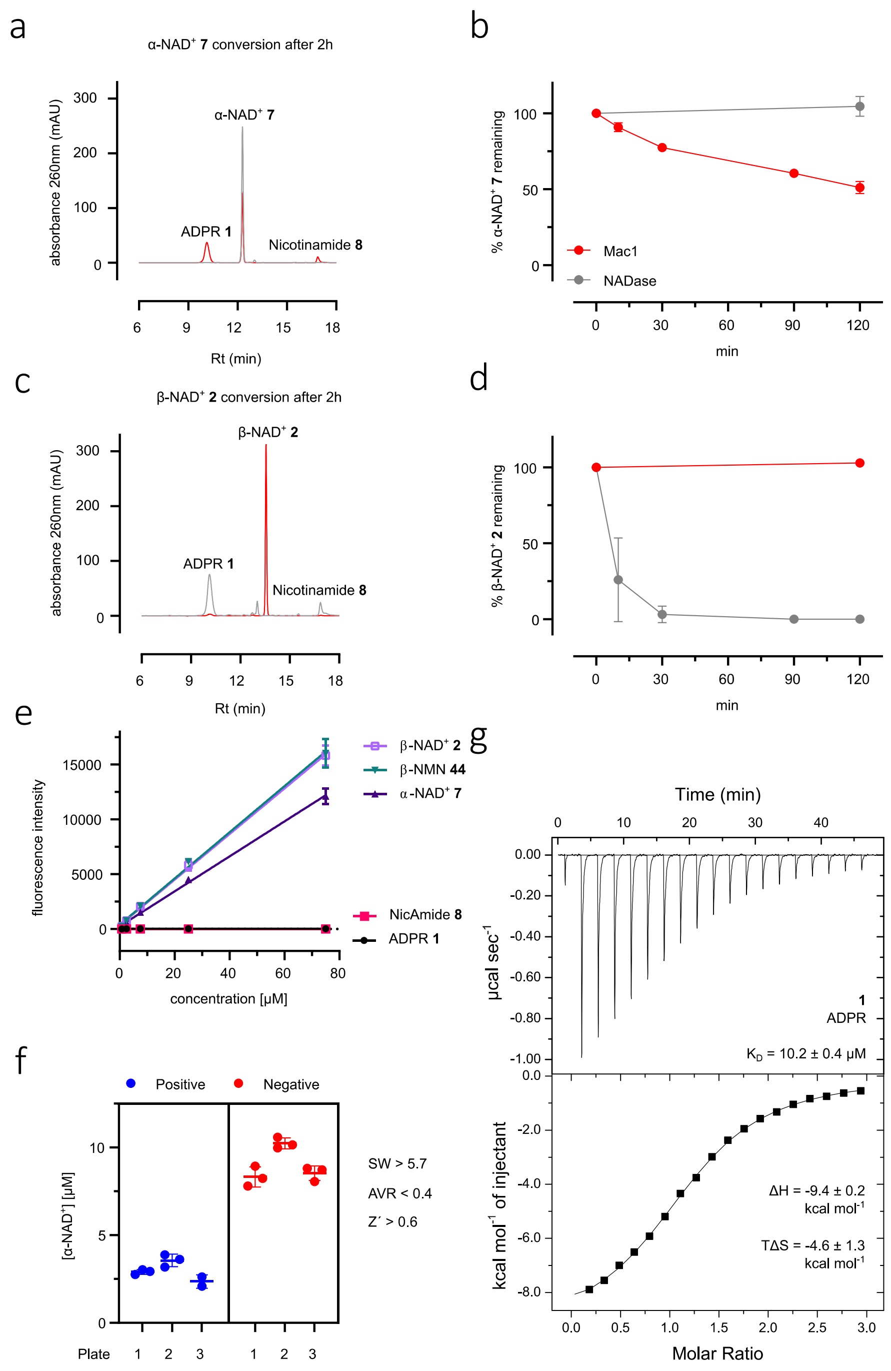

Supplementary Fig. 1: α-NAD$^+$-based assays for Mac1 activity.

(a, c) Representative HPLC chromatograms of the reaction products formed after 2 h incubation of 0.1 mM α-NAD$^+$ **7** (a) or β-NAD$^+$ **2** (c) with wildtype Mac1 (3 µM, red) or *Neurospora crassa* NADase (2 µg/ µl, grey). The experiments were repeated two more times. Rt: retention time. AU: arbitrary unit. (**b, d**) Time course of the reactions of (a) and (c) respectively. **(e)** Test of the specificity and linearity of the microplate derivatisation reaction for different educts and the expected hydrolysis products respectively. Data are shown as mean ± SEM from 3 technical replicates. **(f)** Evaluation of the assay performance in 96-well format. Shown are the remaining α-NAD$^+$ **7** levels after co-incubation with (positive control) or without Mac1 (negative control) of 3 independent assays (plate 1-3) to determine signal window (SW), assay variability ratio (AVR) and robustness (Z´) according to Zhang and coworkers[1S]. **(g)** Representative ITC data of ADPR **1** binding by wildtype Mac1. Top graph shows a thermogram and the bottom graphs display the integrated binding heats fitted to a one-site binding model. The

experiment was repeated two more times yielding K_D, ΔH and $T\Delta S$ as mean ± SD. Source data are provided as a Source Data file.

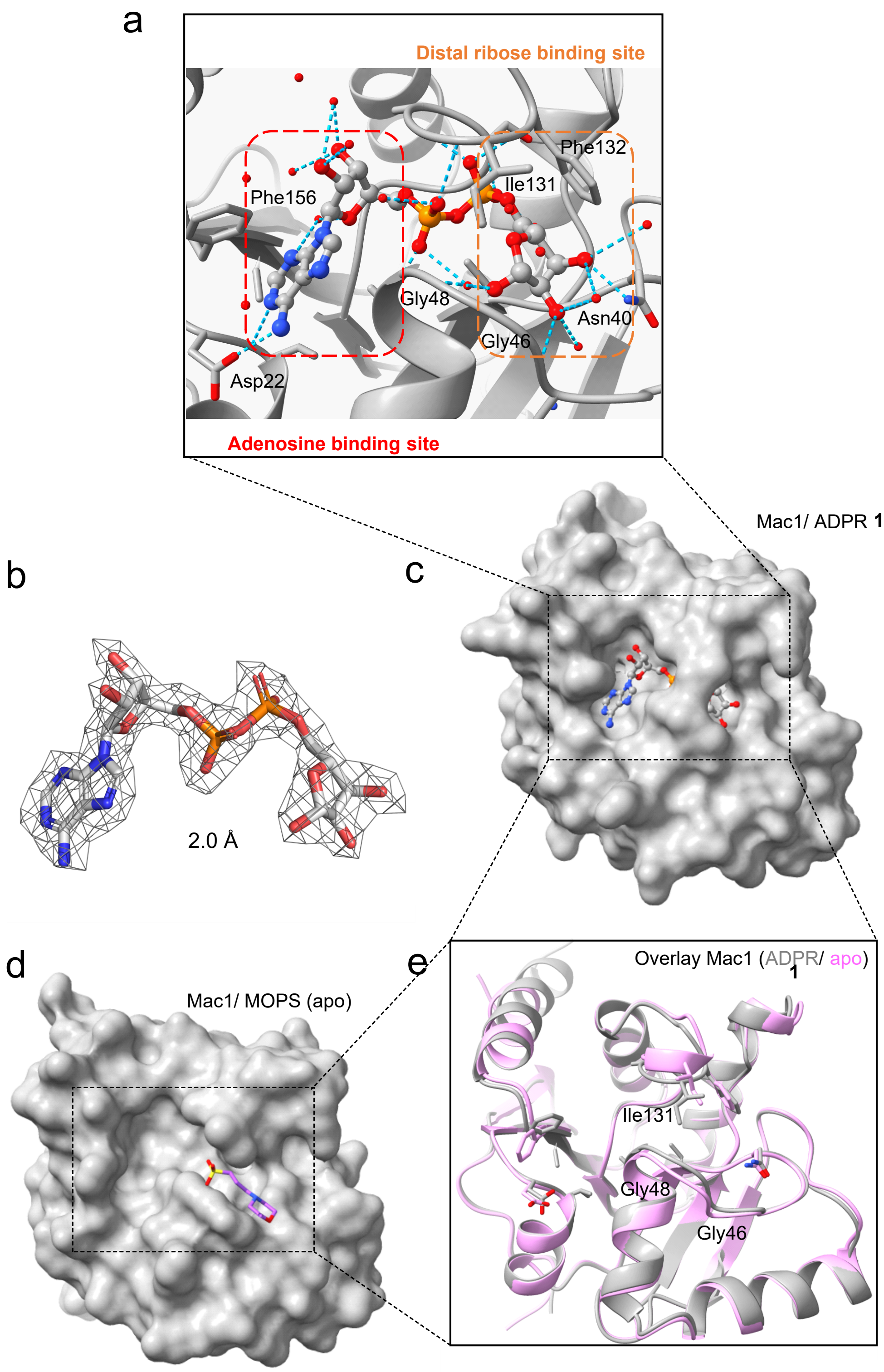

Supplementary Fig. 2: Structure of Mac1 in the presence and absence of ADPR.

(a, c) X-ray crystallography of ADPR **1** in complex with Mac1 (ADPR: PDB 8AZD) as cartoon (a) and surface presentation (c). Dashed blue lines indicate H-bond interactions. **(b)** Electron density (2mFo-DFc, 1σ) for the ligand of a, c. **(d)** X-ray crystallography of MOPS in complex with Mac1 (apo: PDB 8AZC) as surface presentation. **(e)** Superimposition of the Mac1 conformations in the apo form (d) and in complex with ADPR (a, c).

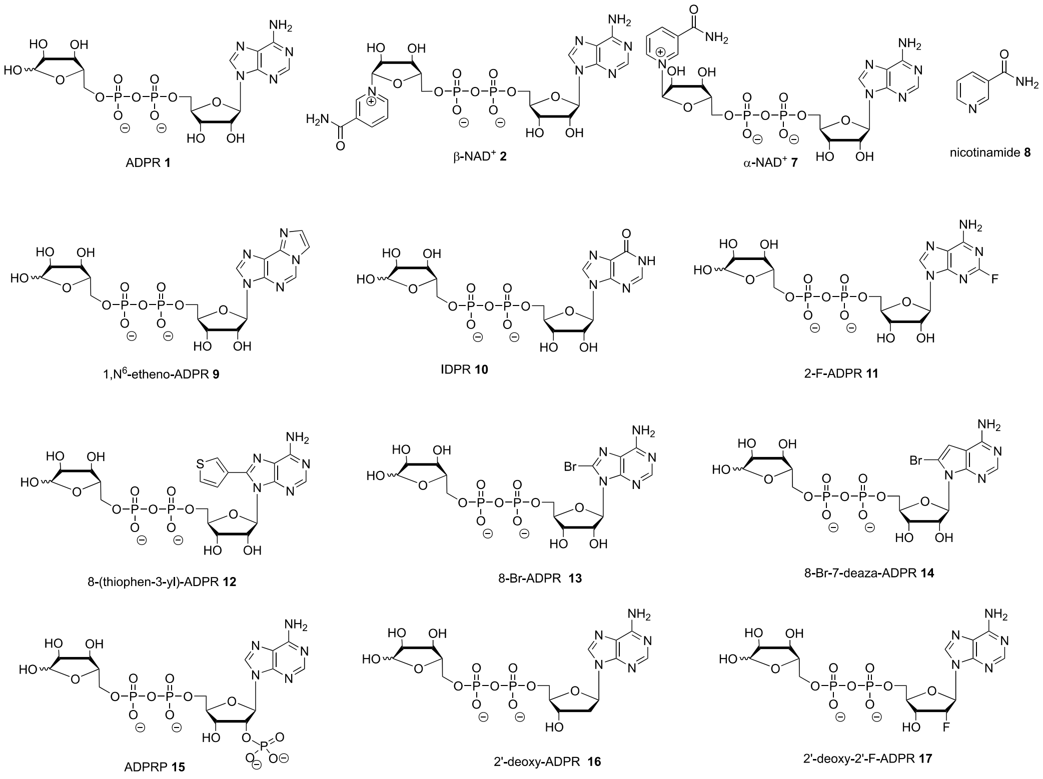

Supplementary Fig. 3: Excerpt of ADP(R) analogs tested in this study.

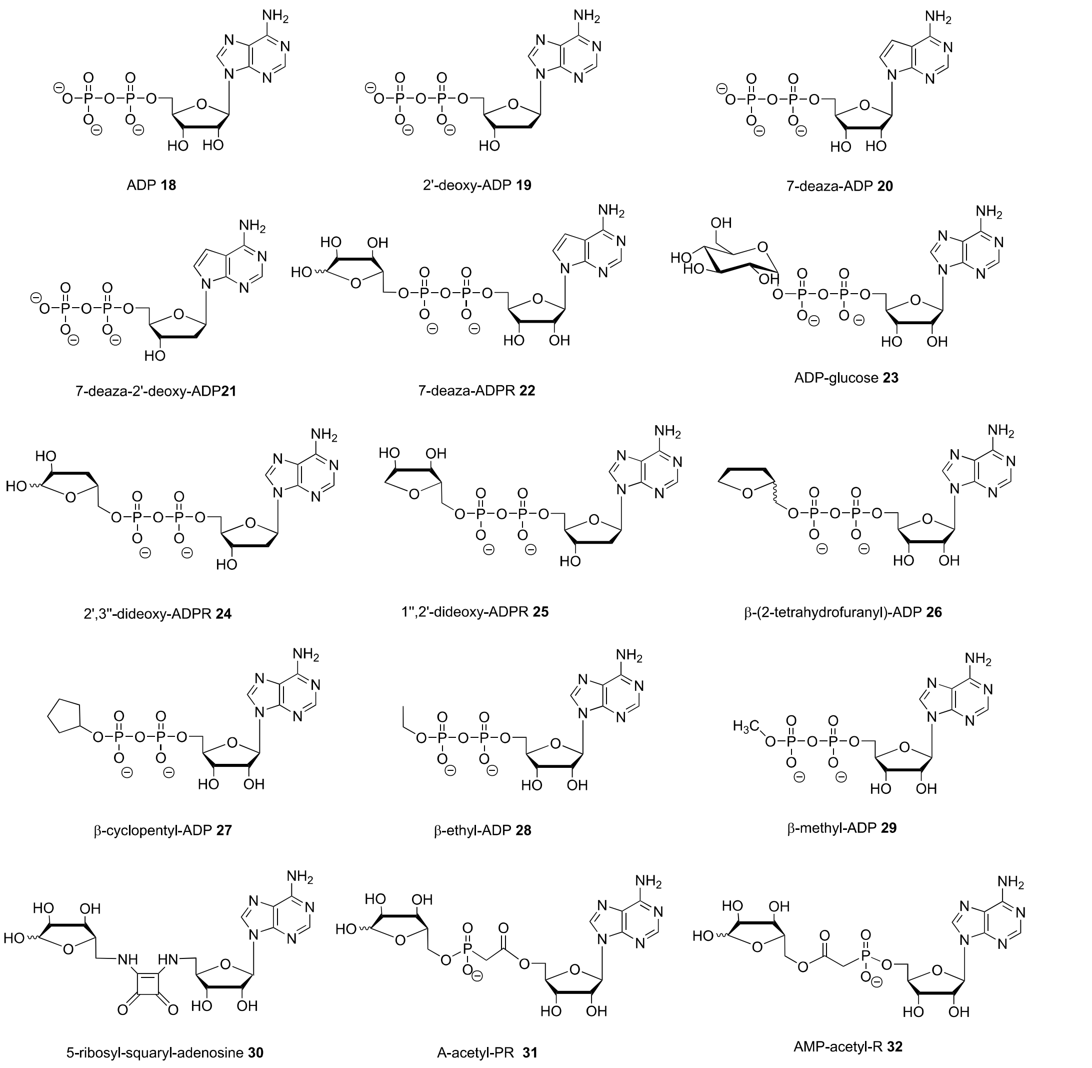

Supplementary Fig. 4: Excerpt of ADP analogs and de-novo synthesized compounds in this study.

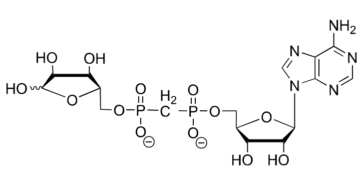

CH$_2$-ADPR **33**

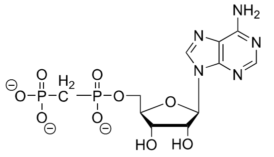

CH$_2$-ADP **34**

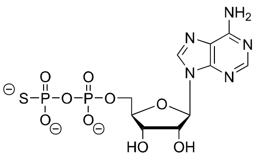

ADP-β-S **35**

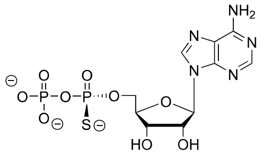

(R$_p$)-ADP-α-S **36**

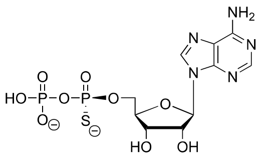

(S$_p$)-ADPα-S **37**

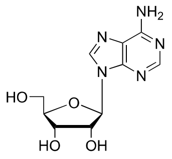

Adenosine **38**

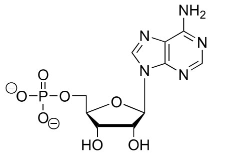

AMP **39**

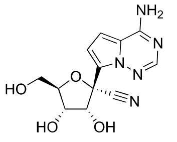

GS-441524 **3**

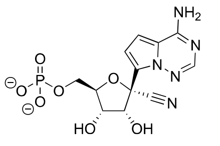

GS-441524-MP **40**

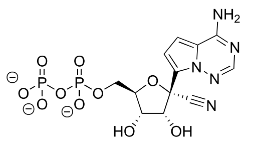

GS-441524-DP **41**

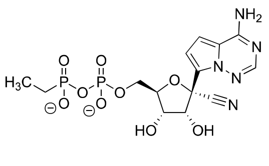

β-ethylphosphonate-phosphate-GS-441524 (ST161) **42**

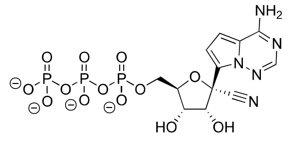

GS-441524-TP **43**

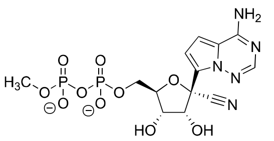

β-methyl-GS-441524-DP **4**

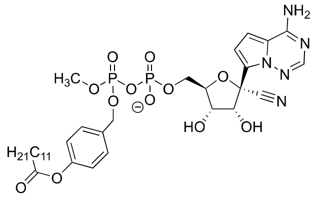

C11-AB-β-methyl-GS-441524-DP (ST135) **5**

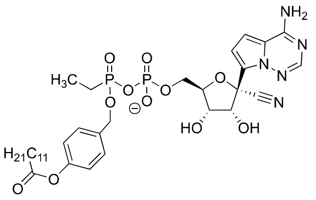

C11-AB-β-ethylphosphonate-phosphate-GS-441524 (ST166) **6**

Supplementary Fig. 5: Excerpt of ADP(R) analogs and de-novo synthesized compounds in this study.

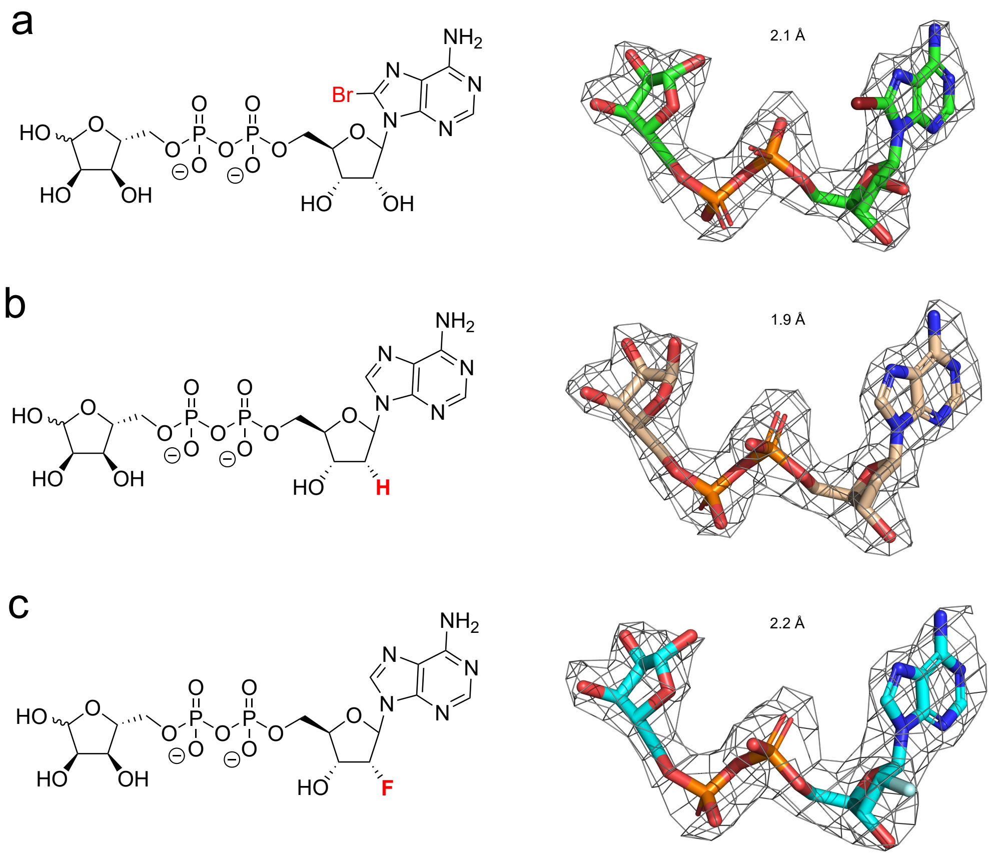

Supplementary Fig. 6: Electron density of Adenosine-modified ADPR derivatives in the Mac1 cocrystals.

Chemical structures and corresponding 2mFo-DFc maps at 1σ for **(a)** 8-Br-ADPR **13** (PDB 8AZM), **(b)** 2´-deoxy-ADPR **16** (PDB 8AZI) and **(c)** 2´-deoxy-2´-F-ADPR **17** (PDB 8AZL).

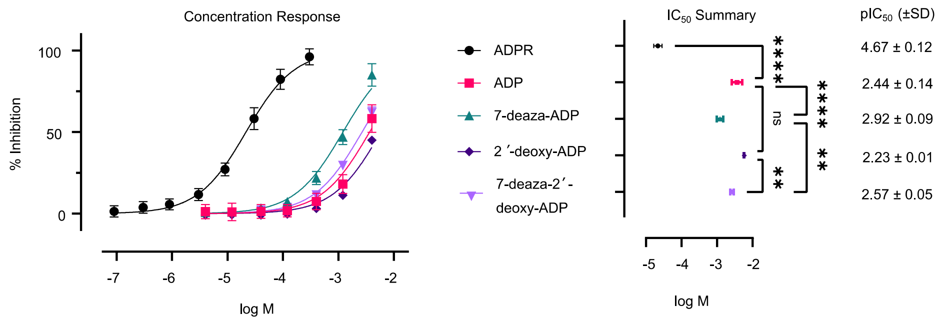

Supplementary Fig. 7: Adenosine-modified ADP derivatives exhibit similar effects on Mac1 activity as their ADPR counterparts.

(a) Concentration-response curves for Mac1 inhibition by Adenosine-modified ADP derivatives. Data were obtained using the microplate assay and the parameters of a sigmoidal model were fitted to the data. The derived pIC_{50} values are shown to the right. Matched experiments using ADPR **1** (black) or ADP **18** (red) as inhibitor were included as control and are shown for comparison. Data are presented as mean ± SD and were tested by one-way ANOVA followed by pair-wise comparison using Šídák's correction. ns: not significant, ** $p \leq 0.01$, **** $p \leq 0.0001$. For all compounds except ADPR **1** and ADP **18**, data are from 3 independent experiments. Since the latter were always included as matched controls, data are from 9 (ADPR **1**) or 5 (ADP **18**) independent experiments respectively. Source data and adjusted p-values are provided as a Source Data file.

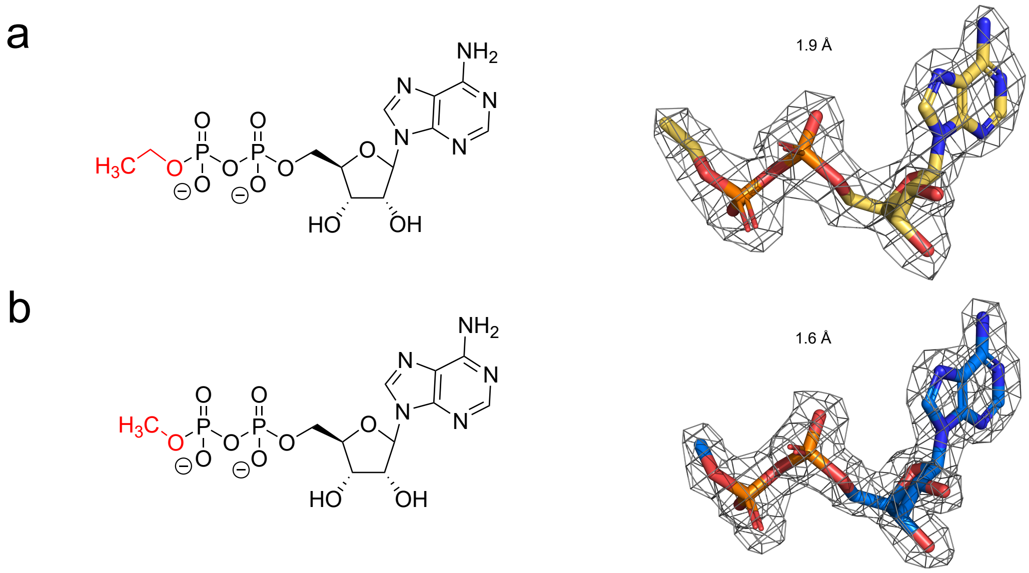

Supplementary Fig. 8: Electron density of β-alkylated adenosine diphosphates in the Mac1 cocrystals.

Chemical structures and corresponding 2mFo-DFc maps at 1σ for **(a)** β-ethyl-ADP **28** (PDB 8AZO) and **(b)** β-methyl-ADP **29** (PDB 8AZP).

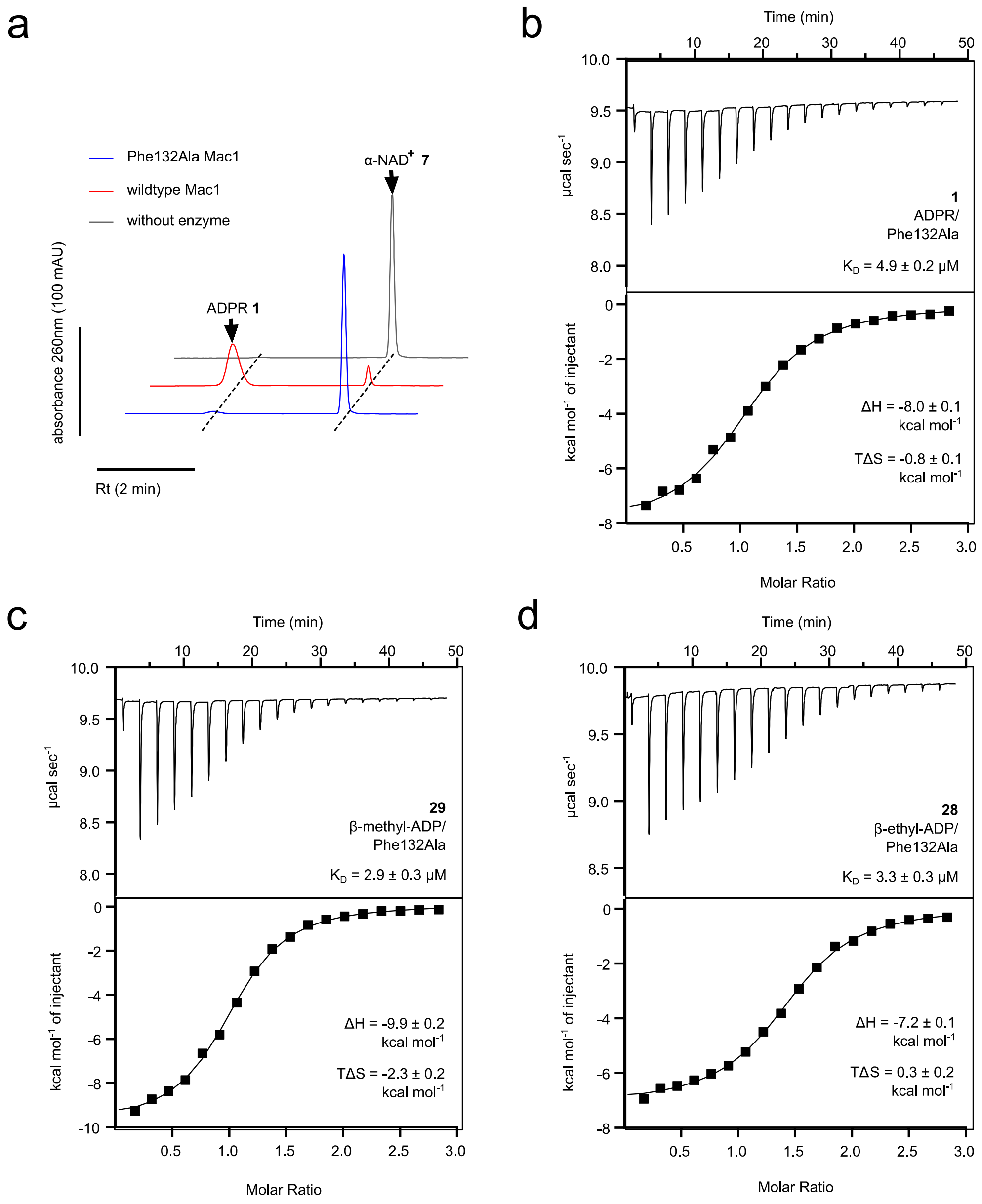

Supplementary Fig. 9: Catalytically inactive Phe132Ala Mac1 binds ADPR, β-methyl- and β-ethyl-ADP with higher affinity than wildtype Mac1.

(a) Representative HPLC chromatogram of the reaction products of 0.1 mM α-NAD$^+$ **7** after incubation with either wildtype Mac1 (red), Phe132Ala Mac1 (blue) or without enzyme (grey) for 20 hours. Dashed lines indicate retention times. The experiment was repeated two more times. Rt: retention time. **(b-d)** Representative ITC data for the binding of ADPR **1** (b), β-methyl-ADP **29** (c) and β-ethyl-ADP **28** (d) to Phe132Ala Mac1. The top graphs show thermograms and the bottom graphs show the integrated values of each titration point fitted to a one-site binding model. The experiments were repeated two more times yielding K_D, ΔH and TΔS as mean ± SD. Source data are provided as a Source Data file.

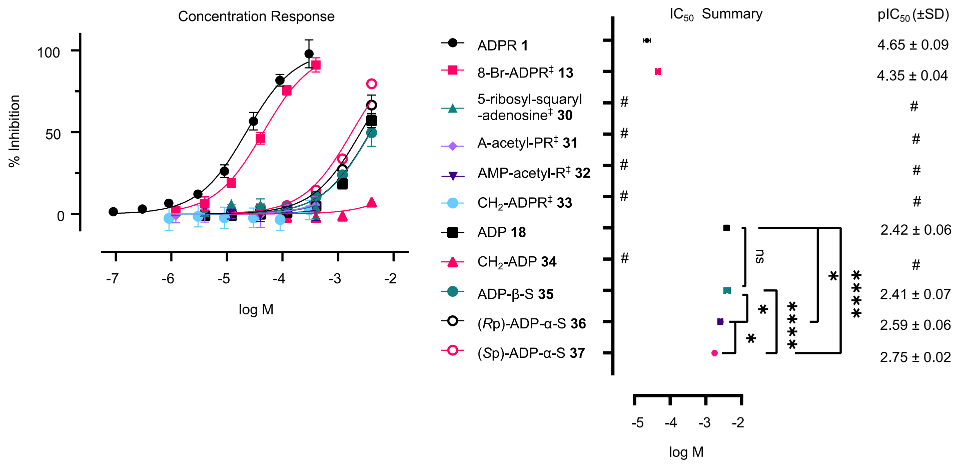

Supplementary Fig. 10: While the pyrophosphate of ADPR can not be replaced by bioisosteres, phosphothioates of ADP show inhibition.

(a) Concentration-response curves for Mac1 inhibition by ADPR derivatives with the pyrophosphate replaced by bioisosteres and ADP derivatives with modified diphosphate. Data were obtained using either the microplate assay or the HPLC assay (‡). The parameters of a sigmoidal model were fitted to the data and the derived pIC_{50} values are shown to the right. In some cases the pIC_{50} are outside the concentration range tested (#). Matched experiments using ADPR **1** or 8-Br-ADPR **13** as inhibitor were included as control and are shown for comparison. Data are presented as mean ± SD and were tested by one-way ANOVA followed by pair-wise comparison using Šídák's correction. ns: not significant, *$p \leq 0.05$, **** $p \leq 0.0001$. For all compounds except ADPR **1** and 8-Br-ADPR **13**, data are from 3 independent experiments. Data for 5-ribosyl-squaryl-adenosine **30** are from 2 independent experiments, while the latter were always included as matched controls, data are from 6 (ADPR **1**) or 5 (8-Br-ADPR **13**) independent experiments respectively. Source data and adjusted p-values are provided as a Source Data file.

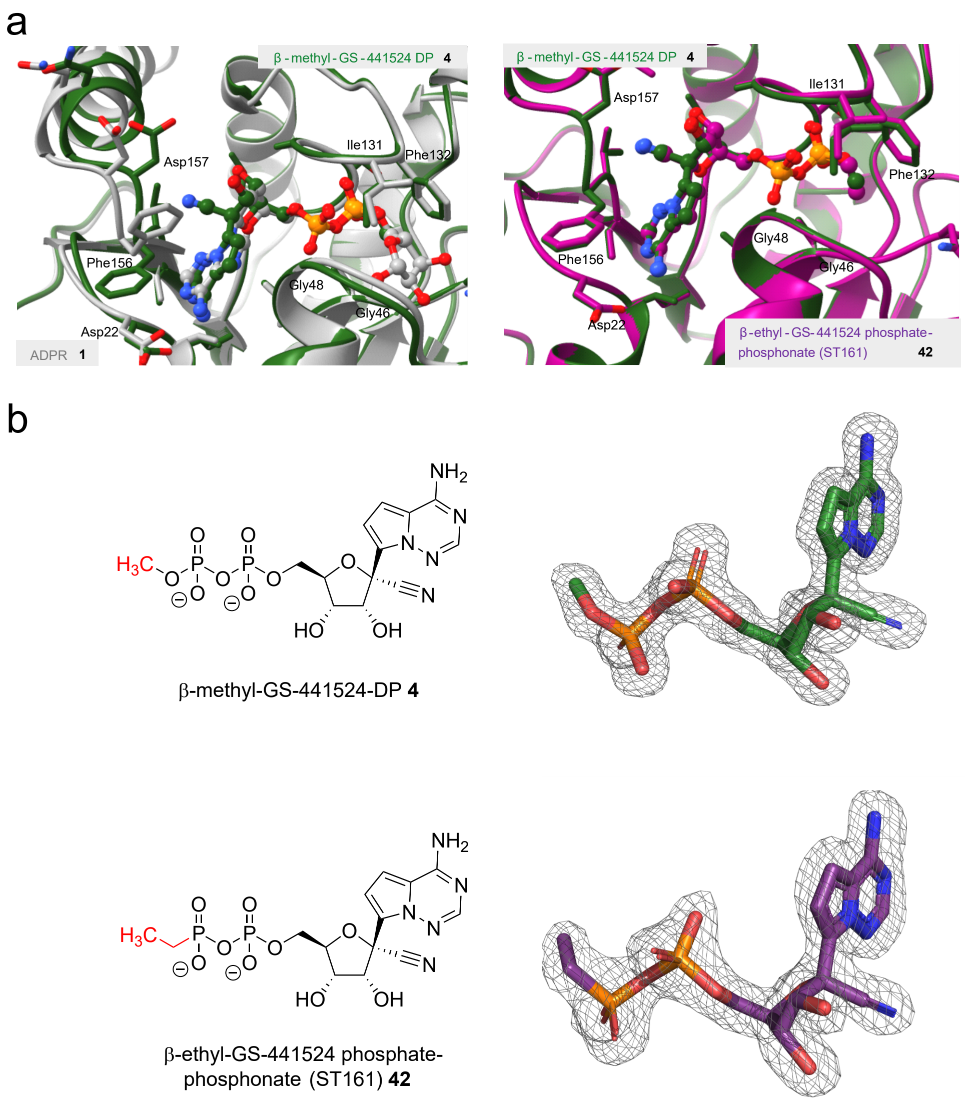

Supplementary Fig. 11: Binding mode and electron density of Mac1 inhibitors.

(a) Structure of the Mac1 complex with β-methyl-GS-441524-diphosphate **4** (green, PDB 9RHO) as superimposition with ADPR **1** (grey, PDB 8AZD) or ST161 **42** (magenta, PDB 9RHN). **(b)** Electron densities (2mFo-DFc, 1σ) of the ligands of the Mac1 cocrystals with β-methyl-GS-441524-diphosphate **4** (green, PDB 9RHO) and ST161 **42** (magenta, PDB 9RHN).

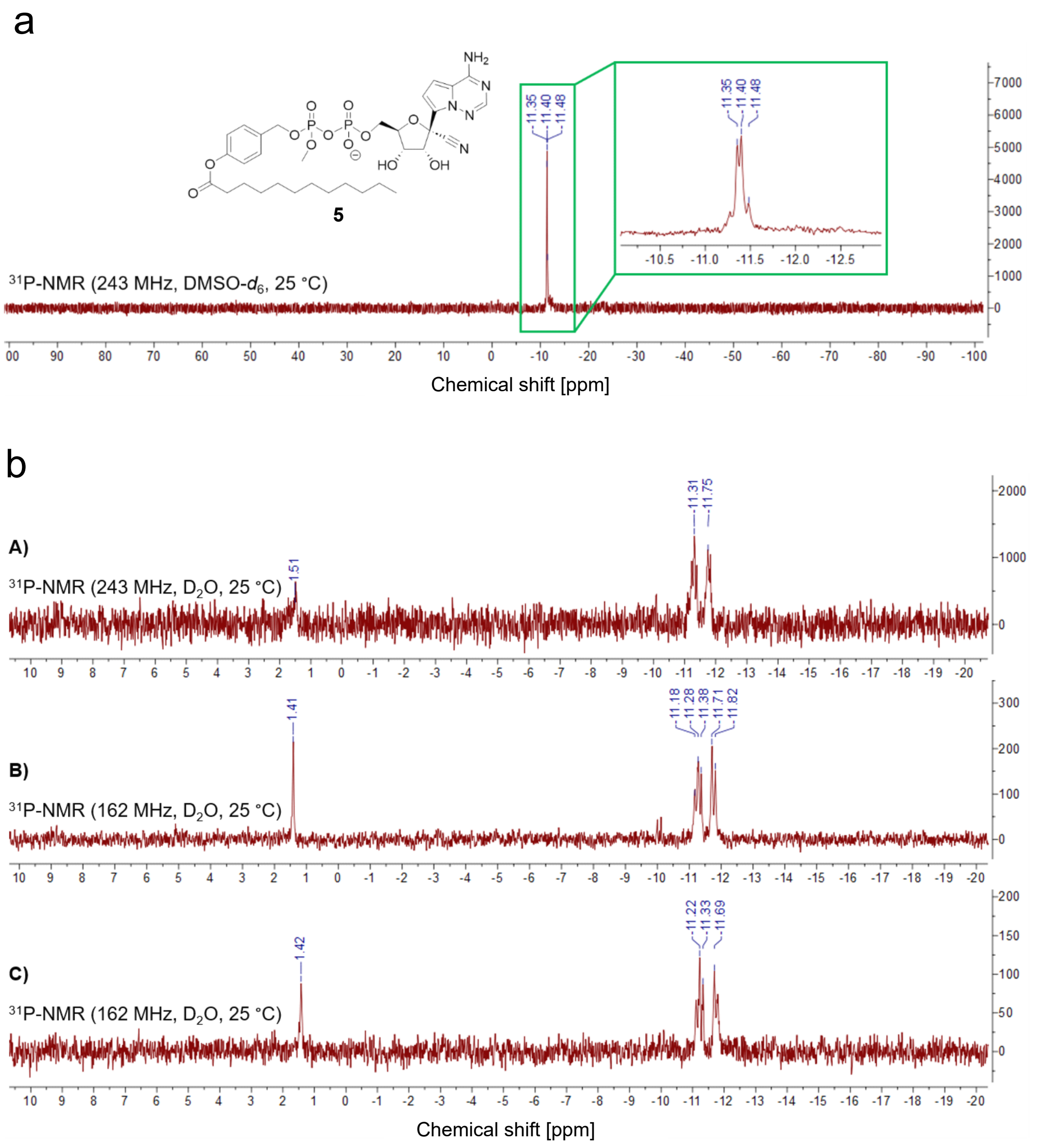

Supplementary Fig. 12: Chemical stability of prodrug 5 during purification.

(a) ^{31}P-NMR spectra of prodrug **5** dissolved in deutero-DMSO and measured at 243 MHz. No more than 5 minutes passed between sample preparation and measurement. The diphosphate signal is highlighted in green. **(b)** ^{31}P-NMR spectra of prodrug **5** after purification by automated reversed phase chromatography on RP-18 silica gel. A) Spectrum after purification of the isolated prodrug **5** for two times. B) Spectrum after purification of the isolated prodrug **5** for three times. C) Spectrum after purification of the isolated prodrug **5** for four times. All spectra were measured in deutero-water and at 243 MHz or 162 MHz. At least 30 minutes elapsed between sample preparation and measurement of diphosphate **5**. Source data are provided as a Source Data file.

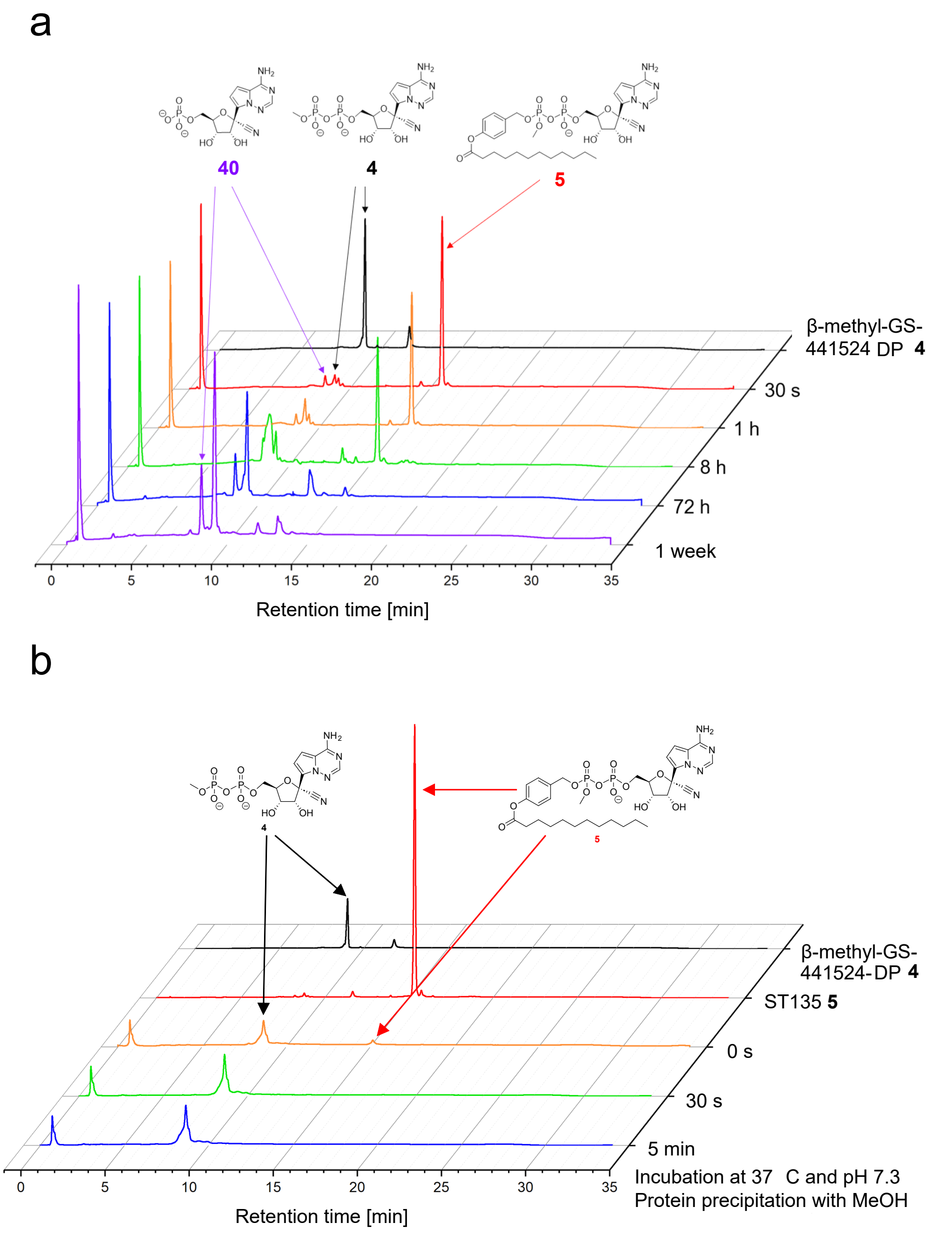

Supplementary Fig. 13: Chemical stability and PLE digest of compound 4 and prodrug 5.

(a) Representative HPLC chromatograms of the PBS hydrolysis study with prodrug **5**. Hydrolysis solution was incubated with PBS at 37°C and pH 7.3. After indicated times (30s, 1 h, 8 h, 72 h, 1 week) aliquots were taken and analysed via HPLC (n=3). **(b)** Representative HPLC chromatograms of the PLE hydrolysis study with prodrug **5**. Hydrolysis solution was incubated with PLE at 37°C and pH 7.3. After indicated times (0s, 30 s, 5 min) aliquots were taken and analysed via HPLC (n=3). Source data are provided as a Source Data file.

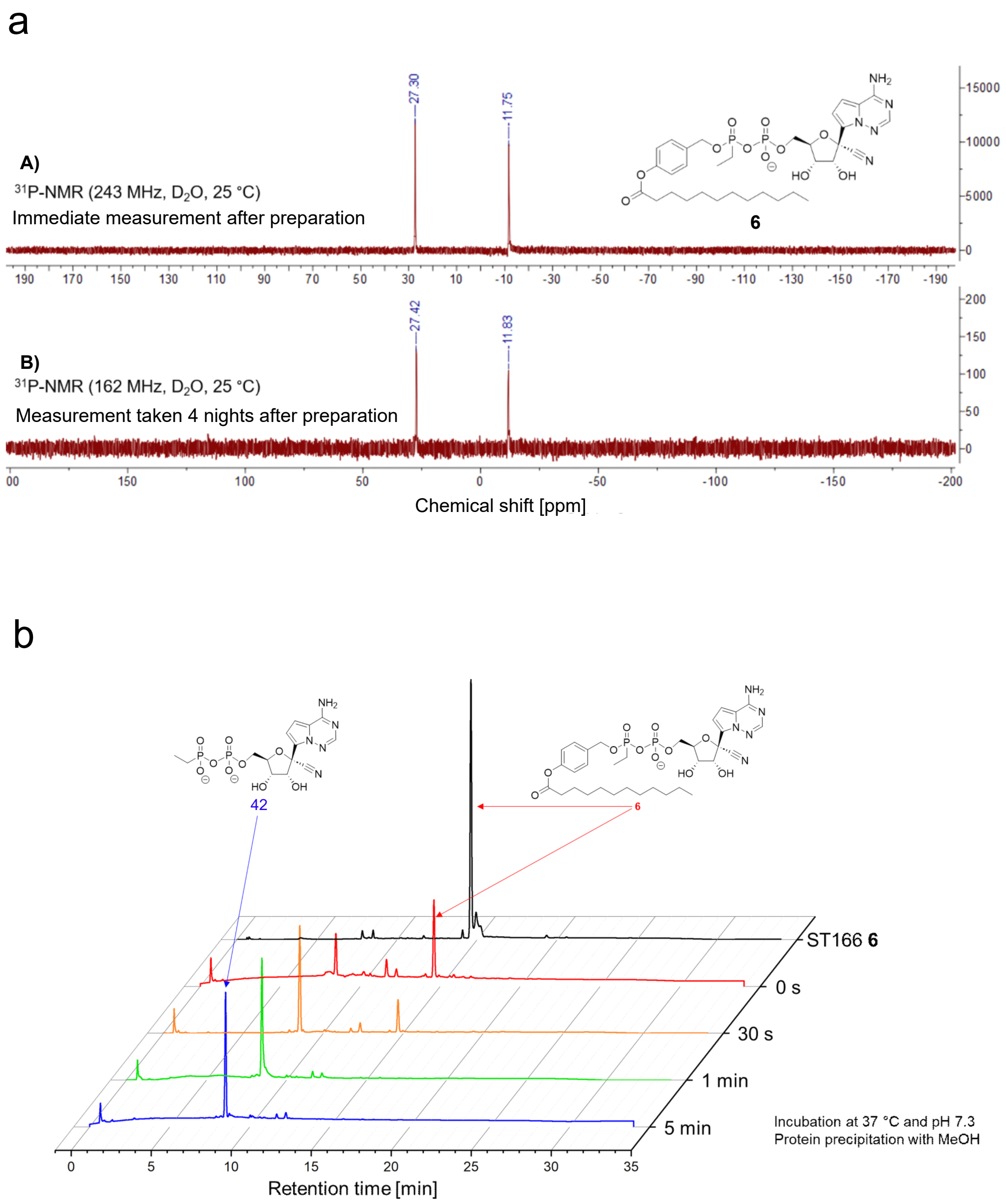

Supplementary Fig. 14: Prodrug 6 is chemically stable.

(a) ^{31}P-NMR spectra of prodrug **6**. A) Spectrum measurement directly after sample preparation. ^{31}P-NMR spectrum was measured in deutero-water and at 243 MHz. B) Spectrum measurement after 4 nights following sample preparation. ^{31}P-NMR spectrum was measured in deutero-water and at 162 MHz. **(b)** Representative HPLC chromatograms of the PLE hydrolysis study with Prodrug **6**. Hydrolysis solution was incubated with PLE at 37°C and pH 7.3. After indicated times (0s, 30 s, 1 min, 5 min) aliquots were taken and analyzed via HPLC (n=3). Source data are provided as a Source Data file.

a

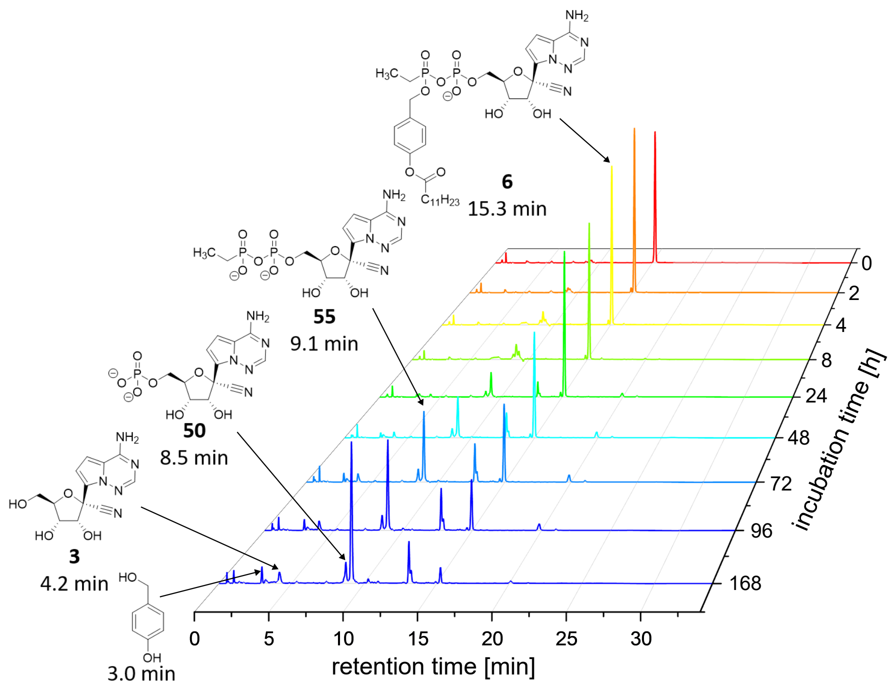

b

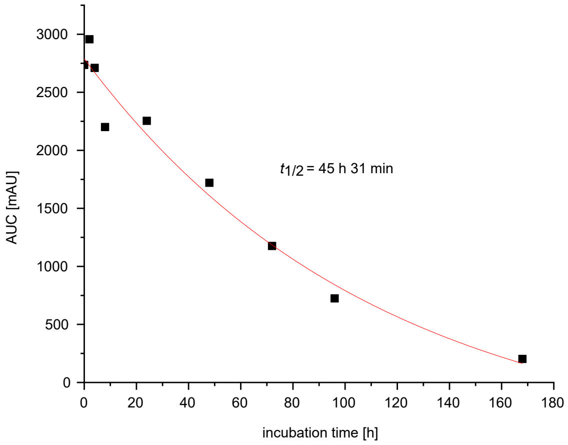

Supplementary Fig. 15: Chemical stability of prodrug 6 in cell medium.

(a) HPLC chromatograms of the hydrolysis study with prodrug 6 (ST166) in RPMI medium. Hydrolysis solution was incubated at 37°C and pH 7.3. After indicated time (2 h, 4 h, 8 h, 24 h, 72 h, 96 h 168 h) aliquots were taken and analysed via HPLC. Signals were recorded at 270 nm absorbance. (b) Hydrolysis kinetics from peak integrals (AUC) of the educt. Source data are provided as a Source Data file.

a

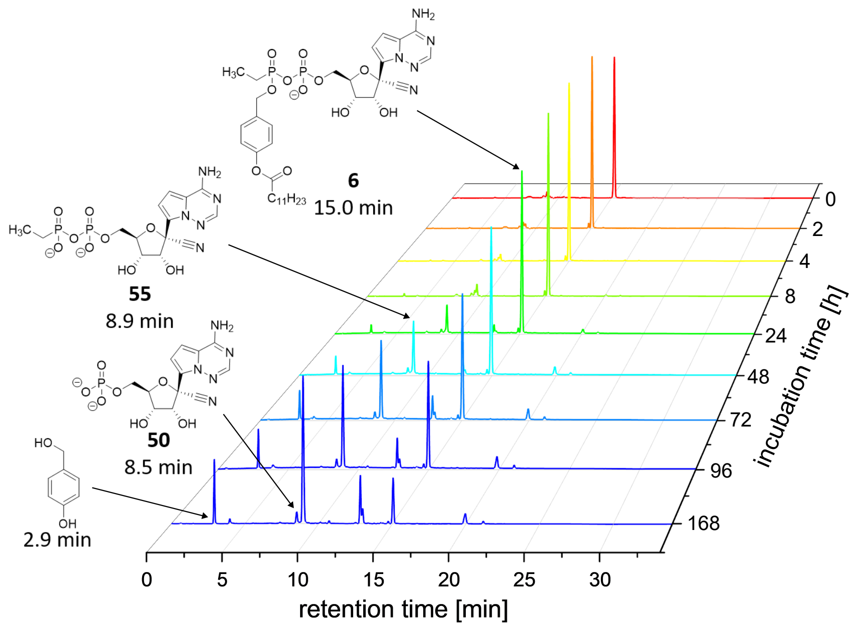

b

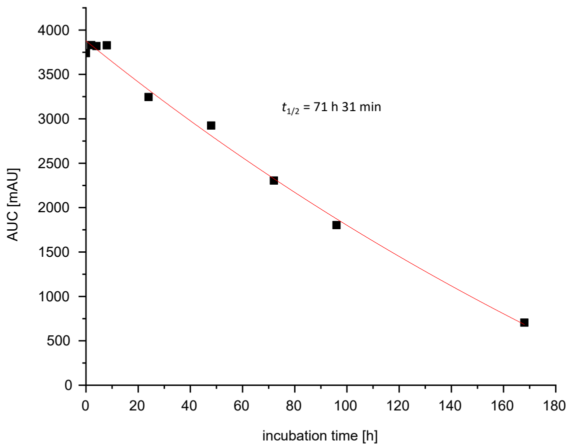

Supplementary Fig. 16: Chemical stability of prodrug 6 in PBS.

(a) HPLC chromatograms of the hydrolysis study with prodrug 6 (ST166) in PBS. Hydrolysis solution was incubated at 37°C and pH 7.3. After indicated time (2 h, 4 h, 8 h, 24 h, 72 h, 96 h 168 h) aliquots were taken and analysed via HPLC. Signals were recorded at 270 nm absorbance. (b) Hydrolysis kinetics from peak integrals (AUC) of the educt. Source data are provided as a Source Data file.

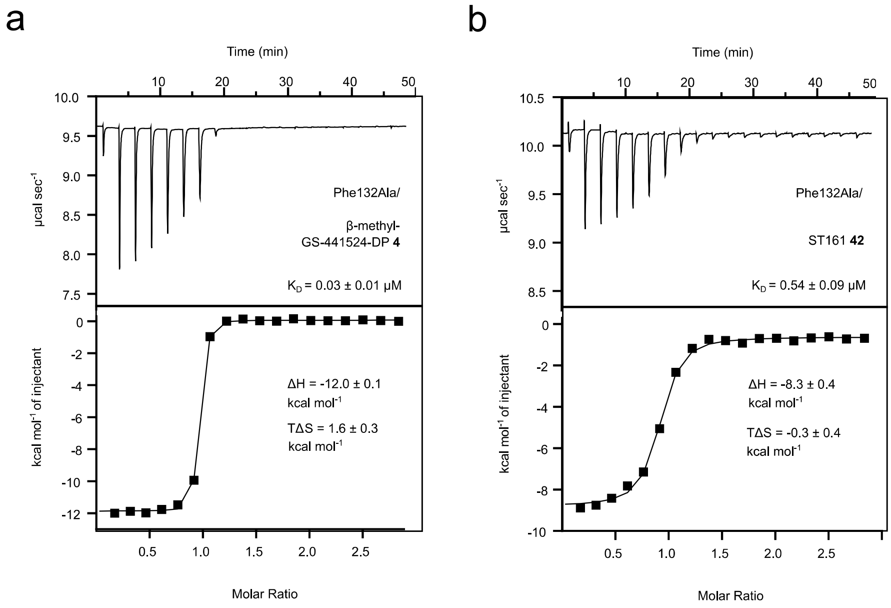

Supplementary Fig. 17: Catalytically inactive Phe132Ala Mac1 binds β-methyl-GS-441524-DP and ST161.

ITC data for the binding of **(a)** β-methyl-GS-441524-DP **4** and **(b)** phosphate phosphonate derivative (ST161) **42** to Phe132Ala Mac1. The top graphs show thermograms and the bottom graphs show the integrated values of each titration point fitted to a one-site binding model. The experiments were repeated two more times yielding KD, ΔH and TΔS as mean ± SD (n=3 experiments). Source data are provided as a Source Data file.

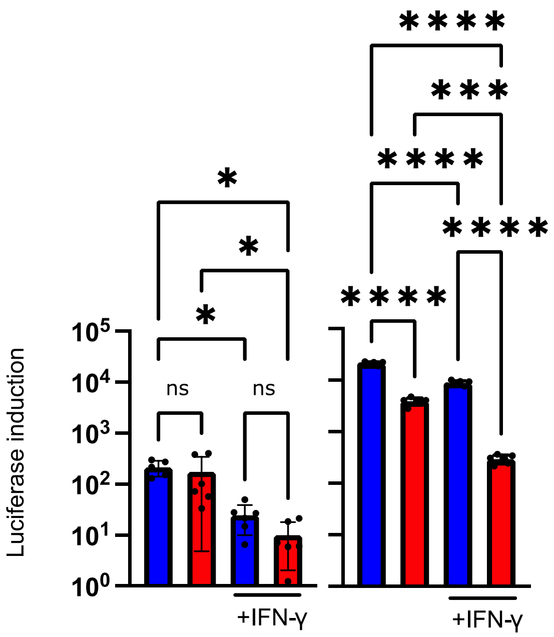

Supplementary Fig. 18: Luciferase activity in cells infected with recombinant wildtype SARS-CoV-2 or the Mac1-inactive mutant with or without IFN-γ pretreatment.

Transcriptional activity measured by the increase in luciferase (LUC) reporter activity of recombinant viruses rWT (blue bars) and rF132A (rF, red bars) 24 hpi on CaLu-3 cells (left) or A549-A/T cells (right) +/- stimulation by IFN-γ (250 U) prior to infection. Individual datapoints from 6 independent experiments are presented, mean and SD are given. Results were tested by two-way ANOVA. ns: not significant, *p ≤ 0.05, *** p ≤ 0.001, **** p ≤ 0.0001. Source data and adjusted p-values are provided as a Source Data file.

a

GUC AUU CUC CAU AGA AGC UA
CAG UAA GAG GUA UCU UCG AU

b

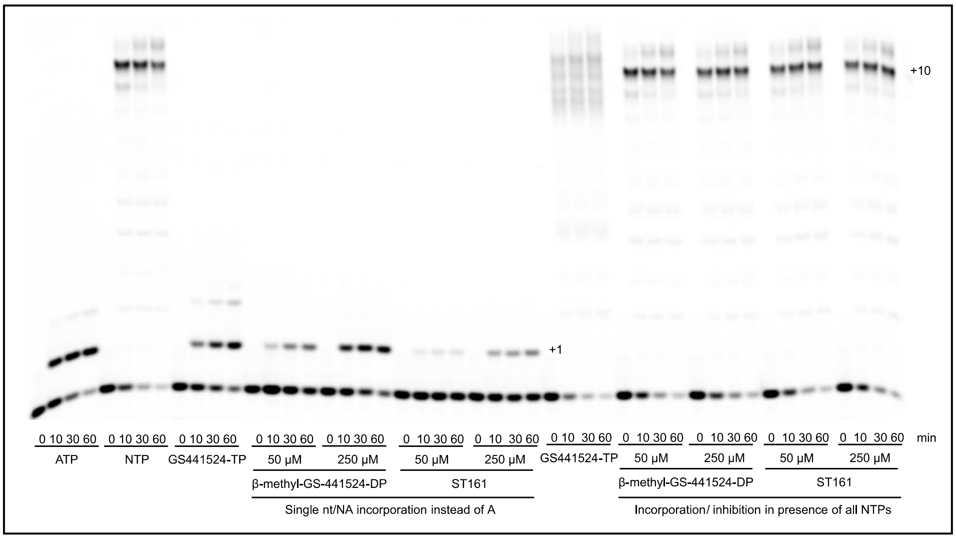

Supplementary Fig. 19: Incorporation of Mac1 inhibitors in comparison to ATP and the remdesivir metabolite (GS-441524-TP) by SARS-CoV-2.

(a) Primer/ template combination used for single or multiple nucleotide elongation (depicted in red). **(b)** PAGE analysis of incorporation reactions. SARS-CoV-2 RTC (500nM) was incubated with the fluorescently labeled primer annealed to the template (500 nM) and 50 µM of NTPs or GS-441524-TP **43** or the given concentrations of β-methyl-GS-441524-DP **4** or ST161 **42** for given time periods. Source data are provided as a Source Data file.

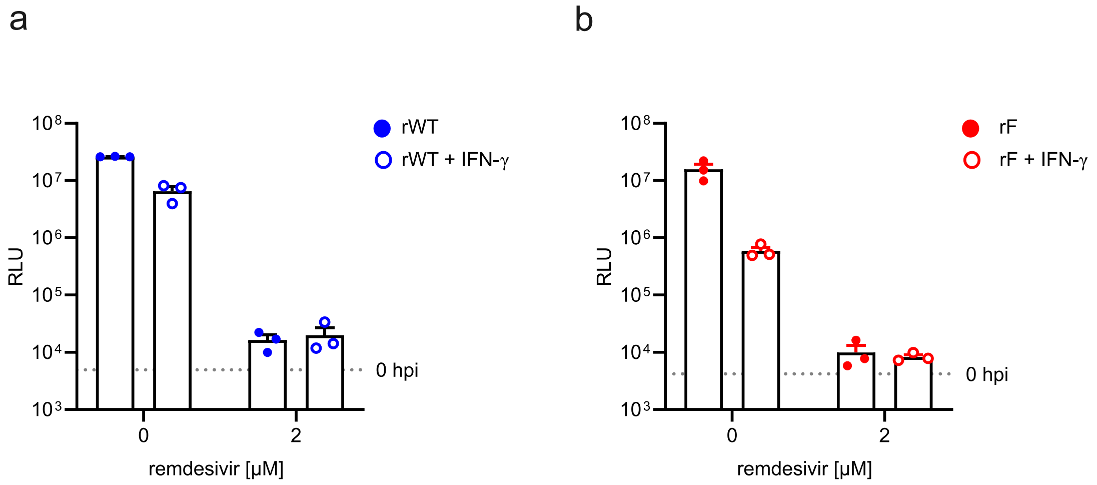

Supplementary Fig. 20: Effect of RdRp Inhibitor remdesivir on luciferase activity of recombinant SARS-CoV-2.

A549-A/T cells were infected with rWT (a) or rF (b) at MOI=1 and treated with Remdesivir with or without IFN-γ (250 U) prestimulation. Raw RLU values were measured 24hpi. A dashed line indicates the luminescence determined at 0 hpi. Bars represent means +/- SEM (n=3). Source data are provided as a Source Data file.

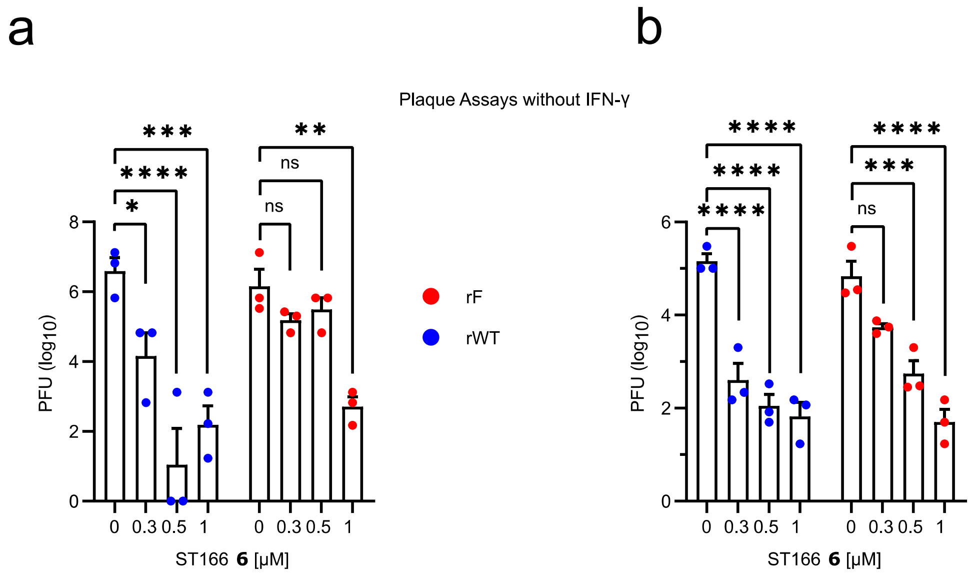

Supplementary Fig. 21: Plaque assay data.

A549-A/T cells were infected with rWT or rF (MOI 0.01) in triplicates, treated with ST166 **6** as indicated, and supernatants were titrated by plaque assay at 48 hpi to obtain infectious viral titers (PFU). Displayed are data from two independent experiments (a,b). Bars represent means +/- SEM. Individual datapoints are shown. Data were tested by one-way ANOVA followed by pair-wise comparison using Šídák's correction. ns: not significant, *$p \leq 0.05$, ** $p \leq 0.01$, *** $p \leq 0.001$, **** $p \leq 0.0001$. Source data and adjusted p-values are provided as a Source Data file.

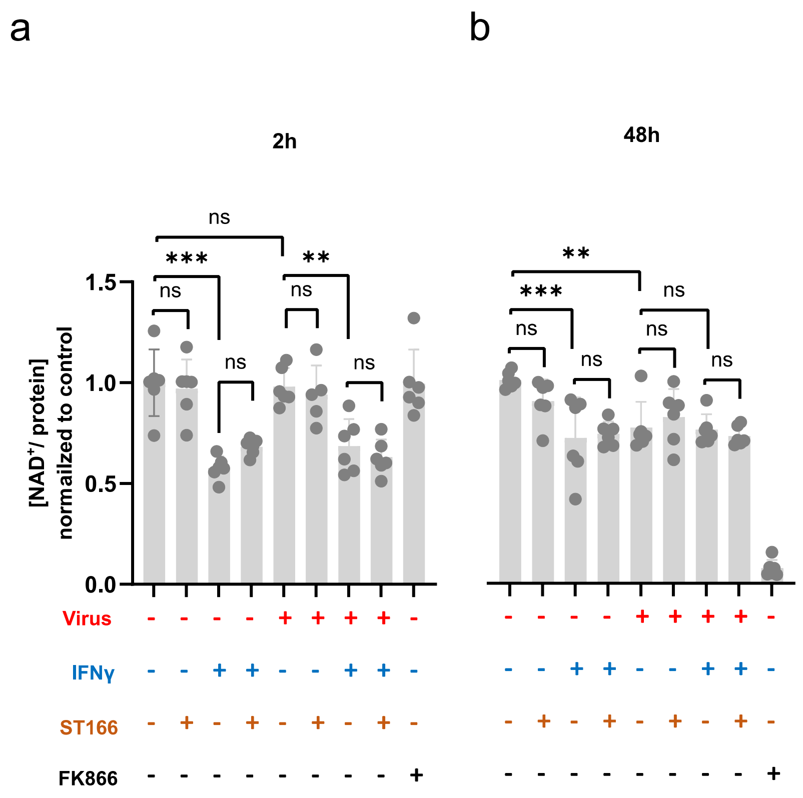

Supplementary Fig. 22: Effect of ST166 treatment on cellular NAD$^+$ levels.

Cellular NAD$^+$ levels were determined by enzymatic cycling assay and divided by total protein amount to compensate for cell number variation. [NAD$^+$/protein] values were normalized to the respective mock controls (uninfected/ untreated) at 2h (a) or 48 h (b) +/- SARS-CoV-2 infection and/or pharmacological intervention (+/- ST166 **6** (0.3 µM), +/- IFN-γ). FK866 (0.01 µM) was used as additional control. Bars represent the mean ± SD (n=5-6 from two experimental days). Data were tested by one-way ANOVA followed by pair-wise comparison using Šídák's correction. ns: not significant, **p ≤ 0.01, *** p ≤ 0.001. Source data and adjusted p-values are provided as a Source Data file.

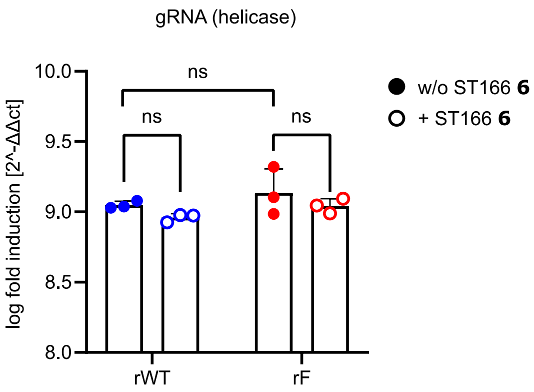

Supplementary Fig. 23: Genomic viral RNA levels upon infection with recombinant SARS-CoV-2 with or without ST166 treatment.

CaLu-3 cells were infected with rWT or rF at MOI=1 with/or withoutST166 **6** (0.5 µM) treatment 16 hpi. Induction of viral helicase (mRNA) as reporter for viral genomic RNA (gRNA) was calculated at 48 hpi relative to uninfected, untreated cells (ct value for viral RNA set to 40 in uninfected cells). Two-way ANOVA with Šídák–corrected post-hoc t-tests of log-transformed data were performed. Bars represent means ± SD (n=3). Datapoints are shown. ns: not significant. Source data are provided as a Source Data file.

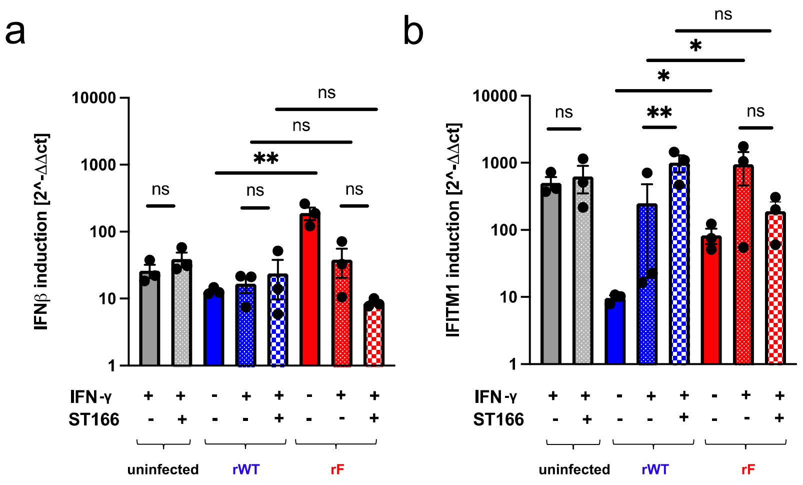

Supplementary Fig. 24: Analysis of IFNβ and IFITM1 transcripts upon infection with recombinant SARS-CoV-2 with or without pharmacological intervention.

(a, b) CaLu-3 cells were either untreated, pretreated with IFN (250 U) and/or infected with rWT (blue bars) or rF (red bars) at MOI=1 and/or additionally treated with 0.1 µM ST166 **6** 1 hpi. Induction of IFNβ (a) or IFITM1 (b) (mRNAs) was calculated at 48hpi by the 2(-ΔΔct) method compared to uninfected, untreated cells. Data points are represented, mean ± SEM are given from 3 independent experiments. Results were tested by two-way ANOVA. ns: not significant, *$p \leq 0.05$, ** $p \leq 0.01$,*** $p \leq 0.001$, **** $p \leq 0.0001$. Source data and adjusted p-values are provided as a Source Data file.

References

[1S] Zhang, JH., Chung, TD. & Oldenburg, KR. A Simple Statistical Parameter for Use in Evaluation and Validation of High Throughput Screening Assays. *J Biomol Screen.* **4**, 67-73 (1999).
