# Supplementary material for: Design, structure-based optimization and antiviral evaluation of potent inhibitors for the macrodomain Mac1 of SARS-CoV-2
Source: Nat Commun. 2026 Jul 27;17:7416. doi: 10.1038/s41467-026-75835-7 (PMC13408504; doi:10.1038/s41467-026-75835-7)
Supplement: Supplementary file 2 — Description of Additional Supplementary Files [file 41467_2026_75835_MOESM2_ESM.pdf]

Description of Additional Supplementary Files

Title: Supplementary Data

Description: Mass Spectrometry data.
