# Supplementary material for: Design, structure-based optimization and antiviral evaluation of potent inhibitors for the macrodomain Mac1 of SARS-CoV-2
Source: Nat Commun. 2026 Jul 27;17:7416. doi: 10.1038/s41467-026-75835-7 (PMC13408504; doi:10.1038/s41467-026-75835-7)
Supplement: Supplementary file 4 — Reporting Summary [file 41467_2026_75835_MOESM4_ESM.pdf]

nature portfolio

Corresponding author(s): Dr. Ralf Fliegert

Last updated by author(s): Jun 29, 2026

Reporting Summary

Nature Portfolio wishes to improve the reproducibility of the work that we publish. This form provides structure for consistency and transparency in reporting. For further information on Nature Portfolio policies, see our Editorial Policies and the Editorial Policy Checklist.

Statistics

For all statistical analyses, confirm that the following items are present in the figure legend, table legend, main text, or Methods section.

n/a	Confirmed	
☐	☒	The exact sample size (*n*) for each experimental group/condition, given as a discrete number and unit of measurement
☐	☒	A statement on whether measurements were taken from distinct samples or whether the same sample was measured repeatedly
☐	☒	The statistical test(s) used AND whether they are one- or two-sided<br>*Only common tests should be described solely by name; describe more complex techniques in the Methods section.*
☒	☐	A description of all covariates tested
☐	☒	A description of any assumptions or corrections, such as tests of normality and adjustment for multiple comparisons
☐	☒	A full description of the statistical parameters including central tendency (e.g. means) or other basic estimates (e.g. regression coefficient) AND variation (e.g. standard deviation) or associated estimates of uncertainty (e.g. confidence intervals)
☒	☐	For null hypothesis testing, the test statistic (e.g. *F*, *t*, *r*) with confidence intervals, effect sizes, degrees of freedom and *P* value noted<br>*Give P values as exact values whenever suitable.*
☒	☐	For Bayesian analysis, information on the choice of priors and Markov chain Monte Carlo settings
☒	☐	For hierarchical and complex designs, identification of the appropriate level for tests and full reporting of outcomes
☒	☐	Estimates of effect sizes (e.g. Cohen's *d*, Pearson's *r*), indicating how they were calculated

Our web collection on statistics for biologists contains articles on many of the points above.

Software and code

Policy information about availability of computer code

Data collection	ChemStation Software (Rev. C.01.05, Agilent Technologies)<br>MicroCal iTC200 analysis software and MicroCal PEAQ-ITC software (Malvern Panalytics)<br>i-Control 2.0 (Tecan)<br>LightCycler 480 SW (v1.5.1, Roche)<br>Agilent OpenLab CDS (Agilent Technologies)<br>TOPSPIN 3.6.4 (Bruker)<br>Mass Hunter Workstation LC/MS Data Aquisition for 6200 series TOF/6500 series Q-TOF (Version 10.1, Agilent Technologies)
Data analysis	ChemStation Software (Rev. C.01.05, Agilent Technologies)<br>GraphPad Prism (v 9.2.0 + 10.6.1)<br>UCSF Chimera (1.17.3 or version X)<br>PyMOL Molecular Graphics System, (v3.1.0, Schrödinger, LLC)<br>AUTOPROC, PHASER, Coot, PHENIX<br>Excel 2016 (Microsoft)<br>Origin 2019 (OriginLab)<br>MestReNova (v14.0.1-23559, Mestrelab Research S.L.)

For manuscripts utilizing custom algorithms or software that are central to the research but not yet described in published literature, software must be made available to editors and reviewers. We strongly encourage code deposition in a community repository (e.g. GitHub). See the Nature Portfolio guidelines for submitting code & software for further information.

Data

Policy information about availability of data

All manuscripts must include a data availability statement. This statement should provide the following information, where applicable:

- Accession codes, unique identifiers, or web links for publicly available datasets
- A description of any restrictions on data availability
- For clinical datasets or third party data, please ensure that the statement adheres to our policy

Source data are provided with this paper. Structural coordinates have been deposited in the RCSB Protein Data Bank under accession numbers 8AZC [https://doi.org/10.2210/pdb8AZC/pdb] (Mac1 apo form), 8AZD [https://doi.org/10.2210/pdb8AZD/pdb] (Mac1 + ADPR 1), 8AZL [https://doi.org/10.2210/pdb8AZL/pdb] (Mac1 + 2´-deoxy-2´-F-ADPR 17), 8AZM [https://doi.org/10.2210/pdb8AZM/pdb] (Mac1 + 8-Br-ADPR 13), 8AZI [https://doi.org/10.2210/pdb8AZI/pdb] (Mac1 + 2´-deoxy-ADPR 16), 8AZO [https://doi.org/10.2210/pdb8AZO/pdb] (Mac1 + β-ethyl-ADP 28), 8AZP [https://doi.org/10.2210/pdb8AZP/pdb] (Mac1 + β-methyl-ADP 29), 9RHO [https://doi.org/10.2210/pdb9rho/pdb] (Mac1 + β-methyl-GS-441524-diphosphate 4), 9RHN [https://doi.org/10.2210/pdb9rhn/pdb] (Mac1 + β-ethyl-phosphate-phosphonate GS-441524 42).

Research involving human participants, their data, or biological material

Policy information about studies with human participants or human data. See also policy information about sex, gender (identity/presentation), and sexual orientation and race, ethnicity and racism.

Reporting on sex and gender	The study does not involve human subjects/clinical trials, therefore gender/sex is not of concern.
Reporting on race, ethnicity, or other socially relevant groupings	The study does not involve human subjects/clinical trials, therefore race/ethnicity and social grouping is not of concern.
Population characteristics	No humans involved therefore not applicable.
Recruitment	No participants were recruited for this study
Ethics oversight	As no human individuals and no human sample material was used, there was no need for ethics oversight.

Note that full information on the approval of the study protocol must also be provided in the manuscript.

Field-specific reporting

Please select the one below that is the best fit for your research. If you are not sure, read the appropriate sections before making your selection.

☒ Life sciences ☐ Behavioural & social sciences ☐ Ecological, evolutionary & environmental sciences

For a reference copy of the document with all sections, see nature.com/documents/nr-reporting-summary-flat.pdf

Life sciences study design

All studies must disclose on these points even when the disclosure is negative.

Sample size	A formal a priori power analysis was not done. However, assay robustness was tested as described in methods and Supplementary Fig. 1f showing that experimental design granted sufficiently low inter- and intra-assay variability and high reproducibilty, according to standard practices in the field while maintaining operational efficiency. From previous publications (Weising et al. 2022 PMID: 36099330, Huchting et al. 2018 PMID: 29906392) we know that for hydrolysis studies the incubation solution need to have at least a concentration of 0.92 mM (chemical hydrolysis) or 0.45 mM (enzymatic hydrolysis) to properly determine the degradation profile of prodrugs via HPLC analysis.
Data exclusions	No data were excluded.
Replication	Experiments were done in replicates as described in methods and figure legends. All repeats were successful.
Randomization	Alternating pipetting schemes were applied for enzymatic compound testing to minimize sampling bias in multi-well formats.
Blinding	No blinding was conducted since analytical instruments readings do not involve subjective assessment.

Reporting for specific materials, systems and methods

We require information from authors about some types of materials, experimental systems and methods used in many studies. Here, indicate whether each material, system or method listed is relevant to your study. If you are not sure if a list item applies to your research, read the appropriate section before selecting a response.

Materials & experimental systems

n/a	Involved in the study	
☒	☐	Antibodies
☐	☒	Eukaryotic cell lines
☒	☐	Palaeontology and archaeology
☒	☐	Animals and other organisms
☒	☐	Clinical data
☐	☒	Dual use research of concern
☒	☐	Plants

Methods

n/a	Involved in the study	
☒	☐	ChIP-seq
☒	☐	Flow cytometry
☒	☐	MRI-based neuroimaging

Eukaryotic cell lines

Policy information about cell lines and Sex and Gender in Research

Cell line source(s)	CaLu-3 cells (Cytion, 305032), Vero E6 cells (ATCC® CRL-1586) and A549-A/T cells (provided by Krzysztof Pyrć, Virology Laboratory at the Malopolska Centre of Biotechnology of the Jagiellonian University), HEK-293T-Ace-2-TMPRSS2 cells (BEI resources, NR-55293)
Authentication	Authentication of all cell lines confirmed by either STR-profiling (CaLu-3) or species-specific PCR (Vero E6,HEK-293T-Ace2-TMPRSS2) by the supplier. A549-A/T were authenticated by STR-profiling (MicroSynth).
Mycoplasma contamination	Cell lines were tested on a regular basis using an enzymatic assay (Lonza MycoAlert) or by PCR (Venor GeM OneStep) and were negative for mycoplasma contamination.
Commonly misidentified lines (See ICLAC register)	None.

Dual use research of concern

Policy information about dual use research of concern

Hazards

Could the accidental, deliberate or reckless misuse of agents or technologies generated in the work, or the application of information presented in the manuscript, pose a threat to:

No	Yes	
☒	☐	Public health
☒	☐	National security
☒	☐	Crops and/or livestock
☒	☐	Ecosystems
☒	☐	Any other significant area

Experiments of concern

Does the work involve any of these experiments of concern:

No	Yes	
☒	☐	Demonstrate how to render a vaccine ineffective
☒	☐	Confer resistance to therapeutically useful antibiotics or antiviral agents
☒	☐	Enhance the virulence of a pathogen or render a nonpathogen virulent
☒	☐	Increase transmissibility of a pathogen
☒	☐	Alter the host range of a pathogen
☒	☐	Enable evasion of diagnostic/detection modalities
☒	☐	Enable the weaponization of a biological agent or toxin
☒	☐	Any other potentially harmful combination of experiments and agents

Plants

Seed stocks

Report on the source of all seed stocks or other plant material used. If applicable, state the seed stock centre and catalogue number. If plant specimens were collected from the field, describe the collection location, date and sampling procedures.

Novel plant genotypes

Describe the methods by which all novel plant genotypes were produced. This includes those generated by transgenic approaches, gene editing, chemical/radiation-based mutagenesis and hybridization. For transgenic lines, describe the transformation method, the number of independent lines analyzed and the generation upon which experiments were performed. For gene-edited lines, describe the editor used, the endogenous sequence targeted for editing, the targeting guide RNA sequence (if applicable) and how the editor was applied.

Authentication

Describe any authentication procedures for each seed stock used or novel genotype generated. Describe any experiments used to assess the effect of a mutation and, where applicable, how potential secondary effects (e.g. second site T-DNA insertions, mosiacism, off-target gene editing) were examined.
