# Supplementary material for: Design, structure-based optimization and antiviral evaluation of potent inhibitors for the macrodomain Mac1 of SARS-CoV-2
Source: Nat Commun. 2026 Jul 27;17:7416. doi: 10.1038/s41467-026-75835-7 (PMC13408504; doi:10.1038/s41467-026-75835-7)
Supplement: Supplementary file 5 — Transparent Peer Review file [file 41467_2026_75835_MOESM5_ESM.pdf]

nature portfolio

Peer Review File

Design, structure-based optimization and antiviral evaluation of potent inhibitors for the macrodomain Mac1 of SARS-CoV-2

Corresponding Author: Dr Ralf Fliegert

This file contains all reviewer reports in order by version, followed by all author rebuttals in order by version.

Version 0:

Reviewer comments:

Reviewer #1

(Remarks to the Author)
“Targeting a viral macrodomain: Design, Structure-Based Optimization and Antiviral Evaluation of Nanomolar Inhibitors of Mac1 of SARS-CoV-2”

Sandmann et al. 2025 Nature Comm

Several groups are currently developing CoV macrodomain inhibitors and exploring their ability to inhibit virus replication and pathogenesis. In this manuscript, the authors explored modifications of ADP-ribose and the common adenine-based metabolite, GS-441524, to further improve its ability to bind and inhibit the SARS-CoV-2 macrodomain (Mac1). The authors walked the reader through each of their modifications, including the ones that did poorly, and ultimately identified a molecule that an pIC50 of 6.62 (~240 nM by my calculation) for the SARS-CoV-2 Mac1 protein and another with a pIC of 5.81 (~1.5 uM). This is notable as there are only a few Mac1 inhibitors that have nanomolar IC50 values (Gahbauer et al. 2023). They used structural biology to demonstrate how each of these (and many other) compounds bind Mac1. Importantly they noted that these compounds would not pass through a cellular membrane, so modifications were made to engineer the more potent molecular, 4 and 42, into pro-drugs. The pro-drugs were successful and so finally the group tested the pro-drugs in antiviral assays and found them to be highly potent against SARS-CoV-2 which some specificity toward Mac1 by using a Mac1 mutant virus.

The authors have done a fine job of identifying novel Mac1 inhibitors and using a pro-drug approach to engineering these compounds to be able to inhibit virus replication. Thus, there are several strong aspects of the paper. However, these are balanced by a few concerns. One issue though is that all of the chemistry described in Figs. 1-3 only minimally contribute to the conclusion of the paper, making the paper harder to read for a broad scientific audience. However, my major concerns are with the virus infection data. It is very clear that these compounds do not just target Mac1, but likely also the polymerase, as the authors even postulated in the discussion. Additional assays are needed to show specificity and provide a more rigorous repression of viral replication. However, it is not imperative clinically that one compound targets just one viral protein. A dual polymerase-Mac1 inhibitor may be more desirable in the clinic than just a Mac1 inhibitor, so the authors may want to think about how they frame the activity of these compounds.

Major Comments
1. Fig. 6a-g: 4 and 42 don’t just target Mac1. A complete deletion of Mac1 only has modest 2-3 fold differences in Calu-3 cells (Alhammad et al. 2023), yet these inhibitors inhibited WT and rF mutant viruses almost completely at concentrations less than 1 uM. Furthermore, considering the EC50 values that they determined are often lower than the IC50 values, it is unclear how much the inhibition of Mac1 in infection contributes to the reduced viral replication, as opposed to inhibiting another viral protein. What is needed, is clear data showing the difference in WT inhibition between untreated and IFN-γ treated cells. As IFN-γ represses Mac1 mutant virus replication, it is also expected that a Mac1-targeting compound would be more effective in the presence of IFN-γ. This does not look to be the case, at least by eyeballing the data. The inhibition of the mutant virus, while supportive, is also complicated by the fact that replication is already dramatically reduced in the presence of IFN-γ (Fig. S16), so it may not be surprising that the inhibitors do not have as great an effect on this virus. In addition, raw luciferase values (like those in Fig. S16) should be shown in the main figures. It is difficult to interpret relative

values when the viruses are at different starting points, for instance, rWT vs rF in the presence of IFN-γ. The authors could at least put one representative figure if luciferase values are highly variable between experiments. Furthermore, an additional virus replication assay, preferably a plaque assay, needs to be done to show the effect on infectious virus. Luciferase levels are often very sensitive and do not necessarily correlate to infectious virus, which is the standard in the field. Again, perhaps the authors could demonstrate that these compounds target both polymerase and Mac1 which could be a more interesting and possibly more useful outcome.

2. Fig. 6h-i: This IFN data could provide important specificity data, but it is somewhat perplexing. While the ISG data does suggest inhibition of Mac1 as the inhibitor enhancing their abundance, the IFN data does not, even though it has been well documented that Mac1- mutant viruses lead to enhanced IFN, as is shown here with the F132A virus. One problem may be in the design of the experiment. The infection was done at an MOI of 1 and harvested at 48 hpi, a time point at which the infection should be largely over. It may be beneficial to do the experiment at either a lower MOI or collect at 24 hpi. In addition, it's unclear why in figure 6h and 6i that the authors did not include a condition where ST166 is added without IFN. Furthermore, the pretreatment with IFN only complicates the analysis of IFNs and also the ISGs. The rF virus has an increase in IFN and IFITM1 without IFN (which was also previously published for a Mac1 deletion virus (Alhammad et al. 2023)), so it unnecessary to do this pretreatment. The authors just need to demonstrate that ST166 increases IFN/IFITM1 levels in a WT infection, but not the mutant. If they can do this, that will show the specificity needed for Mac1 during infection even if it's clear the compounds are targeting multiple viral proteins based on the replication assays.

Another option to clearly show specificity would be to identify drug-resistant mutations in Mac1 or do a CETSA assay (though tricky for an infected cell).

3. Fig. 1-3: The paper could have easily started at Fig. 4 and very little would be different. Furthermore, it is already known that GS-441524 binds and inhibits Mac1 (though weakly) (Tsika et al. 2022; Ni et al. 2021), so again the manuscript could have started at that spot, though I understand that some of the things that were learned in Figs. 1-3 contributed to the newer molecules that were highly effective, but it was only a few things. This may be okay for a chemistry journal but I'm not sure it will be okay in a broader interest journal.

Minor Comments:
1. Fig. 6h,i: Please indicate which IFN is used for pretreatment. I would assume its IFN-γ but this should be indicated in the figure legend and on the figure. In addition, it would be helpful to state the concentration of ST166 in the legend so the reader doesn't have to go the Methods section for this information.
2. Title: As only 1 of the inhibitors throughout the study demonstrated a nanomolar level of inhibition, the title should read "a nanomolar inhibitor" or "novel inhibitors".
3. Line 252: Tsika et al. previously published that GS-441524 inhibits Mac1 activity, thus it should be cited here and likely in other places.
4. Discussion: While it's not necessary to highlight a bunch of publications that have identified Mac1 inhibitors, the discussion should put this work in the context of additional efforts to identify Mac1 inhibitors and at least reference recent manuscripts that have identified Mac1 targeting inhibitors that also repress virus replication (Wazir et al. 2024; Pfannenstiel et al. 2025, Suryawanshi et al. 2024).

Reviewer #2

(Remarks to the Author)
The manuscript describes a substantial body of work related to the discovery of potent and specific inhibitors of the Mac1 SARS-Cov2 viral microdomain, a macrodomain known to counter ADP-ribosylation based immune response. THe authors develop robust chemical phosphorus chemistry to generate monophosphates, diphosphates, phosphophoponates and masked derivatives to facilitate the transition from in vitro enzymatic inhibition studies to cell-based assay. Through a very systematic approach, the authors have developped a robust SAR using a combination of isothermal measurements, in silico analyses, and protein crystallogrophy. The development of membrane permeable phosphorylated derivatives was also very well thought out and conmprehensively demonstrated that the newly generated nucleotides were active against viral infection by targetting MAc1 specifically and with limited off-target activity. However, the mild cell cytotoxicity observed should be evaluated more thoroughly to establish whether the toxicity is due to the quinone being released during the intracellular release of the phosphorylated inhibitors, or otherwise. A masking-agent containing control molecule should be used in cell based assays. It is surprising that the authors did not probe for A(P)DPR or amino modification like N6-methylation (very different effect on the nucleobase pKa).

The authors have done an excellent job at describing the syntheses developed. There are however occasions where the number of mole of product generated being reported cannot correlate to the number of mole of starting material used. Surely there is something odd there. The % yields should have been determined using UV (adenosine 256nm) for quantification and confirmation of the reaction efficacy.
Finally, the scalability of the reported syntheses is of concerns considering the scale at which the reactions were conducted since less than 20mg of starting materials were used for most synthetic conversions. This is unfortunate.

In the reported syntheses, it is imperative that the form of the phosphate reagents is clearly stated: salt + counter cation or free acid form. The stability study and the release rates should have been carried out in cell culture media and not just in

NMR solvents.

Some of the reported chemicals and reagents are known and published entities for which a literature reference should be provided.

The NAD levels in cells post infection should have been measured with and without the membrane permeable inhibitor, with or witoht iFN induction. The effect of Mac1 inhibition could have very unexpected effects on the cellular NAD levels: Inhibition of Mac1 might promote NAD depletion through unchecked cellular ADPR ribosylation or alternatively or stabilize intracellular NAD levels.

Reviewer #3

(Remarks to the Author)
The authors describe an optimization of a previusly characterized Mac1 inhibitor GS-441524 to a cell active prodrug. Initial view is that the manuscript is a nice medicinal chemistry optimization of the compound. However the manuscript appear as two studies put together.

The first part on the ADP/ADPr analogs is a careful study on how these analogs could bind to the Mac1. This does not appear like a very new idea as ADP-ribose has been tried as a starting point to find macrodomain binding compounds before. The work has clear merits as it is carefully conducted and complemented with co-crystal structures of many analogs. The changes in potency of the compounds are mostly detrimental or show what modifications are tolerated. This however appears not to be connnected to the second part which the only study mentioned in the abstract.

In the second study the authors make small modifications to a previously discovered compounds which has been already been used to create nanomolar fluorescent probes etc. (PMID: 37126856) The value and the main interest in the manuscript is the modification of GS-441524 that made it potent, cell permeable and that has some antiviral activity.

Some major points

In the introduction the authors state that most of the inhibitors have been discovered by virtual screening. This statement is not correct as most of the validated inhibitors have been discovered by a range of experimental techniques including HTS based on competition and activity assays, fragment screening in crystallo etc.

Also the statement that the approach used here would be completely different would not be true. Throughout the text they also refer to studies where ADPr analogs have been tested for Mac1 inhibition.

The virus assays would be more robust if there would be target engagement study included in the work. The work with the catalytically inactive Mac1 should be explained better as why to target Mac1 if the inactivating mutation does not inactivate. Authors imply that there could be other mechanism for the prodrug, even a different target. The mutation likely affects compound binding potency and this is not tested.

Some minor points

Figure 1c is not especially clear presentation due to the color choices and it maybe better to use only one color for proteins carbons in the figure as now it changes in every picture. Different color for different compounds would highlight the compound more and clearly show the differences. The views are well selected though. It would be easier for the reader to compare b and c if the ADPr would be in a roughly similar orientation and some summary schematic on the effect of the changes could be used in the main text and not just in the supplement.

It is nice to see the electron density figures in the supplement. In some cases like for compound 29 the density is not super clear for the resolution cut off so it may halp to include the protein residues and waters also. PDB validation reports were not provided.

Prodrug structures should be included in the main manuscript and not just in the supplement.

In the text it is not clear if the ADPr Mac1 complex was solved again by authors as no reference or comparison to previous structures is given. The structure just appears.

There are some typos and eg. "compunds" in the abstract all the NAD+ are missing the +.

Version 1:

Reviewer comments:

Reviewer #1

(Remarks to the Author)
I want to thank the authors for their attention to detail on the comments, and the manuscript has been improved substantially. I would specifically mention that the new IFN/ISG data increased my confidence that the compound targets Mac1 during infection. However, there are still some issues that need to be further addressed, mostly involving interpretations of the results in Fig. S20 and S21. Most are textual changes, but some additional controls and experimental repeats for the current experiments are also needed.

Comments

1. First, in the title either all terms need to be capitalized, or they all should be lowercase. i.e. viral macrodomain should be Viral Macrodomain based on the way the rest of the title is written.
2. Line 50: Should be active, not activate.
3. Lines 64-69. The final sentences of this paragraph are not as to why they transitioned from ST135 (5) to ST166 (6). It should be stated more clearly that compound 5 had reduced stability and specificity in antiviral assays, necessitating the production of compound 6.
4. Figure 6. From this figure it was unclear exactly which molecule is ST135 (5). This should be labeled.
5. The IFN/IFITM1 is perhaps the best data to show the specificity, and to do this experiment the authors modified their normal infection protocol to start drug-treatment at 16 hpi with a high MOI to likely ensure that the replication of each virus was only minimally impacted considering they used a higher dose of compound (0.5 uM). These changes to their general procedures should be described in the results, not just in the methods. Also, it would be prudent to include viral RNA levels in this assay to confirm that there were no drastic differences in viral RNA amounts with/without treatment or comparing the mutants.
6. Supplemental Figure 20a-b should be included in the main manuscript. These results are critical for the interpretation of the data. Also, what is the limit of detection in these assays? A control using an established drug (i.e. GS441524 – inhibits polymerase activity) should be included. The graphs themselves should probably be shown on a log-scale to better interpret what is happening at the higher concentrations of drug. Is the dynamic range of the assay only 10-fold, or is it several logs? If the dynamic range is not very large than the conclusion that the mutant virus is more resistant to the drug may not be true. But if negative controls are several logs lower than the positive control, then I agree with their assessment that rF is more insensitive to the drug. Also, I can barely read the numbers on the y-axis, these need to be increased in size.
7. I don’t get what is going on with Figure S20c. In the data in S20a-b, the compound at these concentrations had nearly identical effects on rF and rWT in the absence of IFN-g. However, in c, the compound reduced rWT nearly 5-logs while only affecting rF by at most 1-log. The other problem is that if an enzyme dead mutant has no defect without IFN-g then the drug should not impact WT virus replication if it’s targeting this enzyme activity (also the Mac1 deletion virus did not have a defect in these cells). Thus, the impact on WT virus would have to be non-specific, which the authors acknowledged in other parts of the paper. But the compounds also did not impact the mutant virus, which is very weird. The only explanation I can come up with is that the rF virus was either not treated with IFN-g while the rWT virus was, or that different amounts of compounds were used for each virus. The authors need to redo this assay or have a logical explanation for the discrepancy in their results.
8. The data in Fig. S21 is not consistent with a role for Mac1 in maintaining NAD+ levels. While the authors make an observation of lower NAD levels at 2 hpi in the presence of IFN-g and ST166, the difference between that group and the IFN-g only treated samples is likely not significant. Furthermore, viral protein production does not typically begin until 4-6 hpi, so the amount of Mac1 in the cell at that time is either minimal or completely absent. Thus, the conclusions on lines 444-445 should be modified or the experiment should instead be done at about 8 hpi.

Reviewer #2

(Remarks to the Author)
The modifications that have been incorporated in the revised manuscript, including supplementary data, are compeling and present a cohesive and robust case as to the novelty of this new class of pro-drug antiviral compounds and their mechanism of action against an innovative target.
The authors have addressed comprehensively all my comments and concerns. As such, I strongly support its publication.

Reviewer #3

(Remarks to the Author)
The authors have improved the manuscript based on the suggestions and have addressed the concerns well. I have listed some minor improvement suggestions still below.

Although the connection between what initially appeared to be two separate studies has improved, it could still be strengthened to help guide the reader through the narrative. Because the manuscript presents a large amount of data, establishing a clear storyline and smooth flow is particularly important.

The discussion of the ADPR analog analysis remains rather lengthy, as many of the described results do not clearly inform the final conclusions. Figure 4 provides a helpful overview of these findings and is appreciated. However, it is currently underutilized and could be integrated more effectively into the discussion. For example, it would be better to refer back to the figure rather than presenting abrupt statements such as “Next, we synthesized β-methyl-GS-441524-DP 4.”.

The discussion section now acknowledges several potential limitations, but these could be elaborated further. The selectivity analysis is based on only two additional macrodomains, making this aspect relatively limited. This limitation could be more explicitly discussed, as it represents an important area for future work rather than a strength of the current manuscript. This especially as the inhibitors are nucleoside analogs.

Finally, several sections contain multiple one-sentence paragraphs. Some rephrasing and merging of these paragraphs would help improve readability and better connect the experimental results with the corresponding interpretations.

Version 2:

Reviewer comments:

Reviewer #1

(Remarks to the Author)
Thanks for the effort on each of these comments. I have no further concerns and endorse the publication of this manuscript.

Reviewer #3

(Remarks to the Author)
The authors have addressed all my concerns and incorporated the suggested improvements, enhancing the flow of the manuscript.

Open Access This Peer Review File is licensed under a Creative Commons Attribution 4.0 International License, which permits use, sharing, adaptation, distribution and reproduction in any medium or format, as long as you give appropriate credit to the original author(s) and the source, provide a link to the Creative Commons license, and indicate if changes were made.
In cases where reviewers are anonymous, credit should be given to 'Anonymous Referee' and the source.
The images or other third party material in this Peer Review File are included in the article’s Creative Commons license, unless indicated otherwise in a credit line to the material. If material is not included in the article’s Creative Commons license and your intended use is not permitted by statutory regulation or exceeds the permitted use, you will need to obtain permission directly from the copyright holder.
To view a copy of this license, visit https://creativecommons.org/licenses/by/4.0/

Manuscript: Targeting a viral macrodomain: Design, Structure-Based Optimization and Antiviral Evaluation of Potent Inhibitors for Mac1 of SARS-CoV-2

ID: NCOMMS-25-56595-T

Response to reviewers

We would like to thank the reviewers for their constructive comments, which we have all addressed in the revised manuscript. We believe that these comments and our revision have improved the manuscript significantly. Listed below are our general changes to the manuscript as well as our point-by-point responses to the comments of the reviewers.

General changes in the the revised manuscript:

- adjusted the list of authors (incl. affiliations) and the respective contributions.
    - Celine Fischer affiliated to Organic Chemistry, Department of Chemistry, University of Hamburg, 20146 Hamburg, Germany (synthesized compounds and collected and analysed NMR and LC-MS data and performed and analysed chemical hydrolysis and PLE studies)
    - Barbara Selisko and Bruno Canard both affiliated to Laboratoire Architecture et Fonction des Macromolécules Biologiques (AFMB), CNRS, Aix-Marseille Université, UMR7257, Marseille, France (performed SARS-CoV-2 polymerase assays)
- adjusted the abstract to better reflect the SAR part of the manuscript.
- Reorientation of the chemical structure of ADPR in Fig. **1b** to facilitate interpretation of the structure in Fig. 1c.
- Former Supplementary Fig. **5** (SAR) was moved to the main manuscript as new Fig. **4**.
- Former Supplementary Fig. **12** (prodrug concept and synthesis) was moved to the main manuscript as new Fig. **6**.
- adjustment of the color of the protein carbon to grey (better contrasting the coloured ligands) in Figs **1c**, **2e**, **3d**, **5e** (formerly 4e), and **7e** (formerly **5e)**.
- addition of new Supplementary Fig. 19, showing results of experiments to evaluate β-methyl-GS-441524 diphosphate **4** and ST161, β-ethyl-phosphonate phosphate GS-441524 **42** as potential substrates and/or inhibitors of the minimal replication/ transcription complex (RTC) of SARS CoV-2
- addition of new Supplementary Figs 15 and 16, showing stability of prodrug ST166 in cell culture medium and phosphate buffered saline (PBS)
- addition of new Supplementary Fig. 21, showing NAD-levels in CaLu-3 cells under various conditions (+/- IFN-gamma, +/- SARS-CoV-2 infection, +/- ST166, +/- FK-866)
- addition of new Supplementary Fig. 17, showing the ITC data for binding of β-methyl-GS-441524 diphosphate and ST161 to the F132A mutant of Mac1

Reviewer #1:

Several groups are currently developing CoV macrodomain inhibitors and exploring their ability to inhibit virus replication and pathogenesis. In this manuscript, the authors explored modifications of ADP-ribose and the common adenine-based metabolite, GS-441524, to further improve its ability to bind and inhibit the SARS-CoV-2 macrodomain (Mac1). The authors walked the reader through each of their modifications, including the ones that did poorly, and

ultimately identified a molecule that an pIC50 of 6.62 (~240 nM by my calculation) for the SARS-CoV-2 Mac1 protein and another with a pIC of 5.81 (~1.5 uM). This is notable as there are only a few Mac1 inhibitors that have nanomolar IC50 values (Gahbauer et al. 2023). They used structural biology to demonstrate how each of these (and many other) compounds bind Mac1. Importantly they noted that these compounds would not pass through a cellular membrane, so modifications were made to engineer the more potent molecular, 4 and 42, into pro-drugs. The pro-drugs were successful and so finally the group tested the pro-drugs in antiviral assays and found them to be highly potent against SARS-CoV-2 which some specificity toward Mac1 by using a Mac1 mutant virus.

The authors have done a fine job of identifying novel Mac1 inhibitors and using a pro-drug approach to engineering these compounds to be able to inhibit virus replication. Thus, there are several strong aspects of the paper.

Author response (AR): We thank the reviewer for the very detailed and helpful review and comments, as well as acknowledging the novelty of our approach and our contribution to the pressing need for potent Mac1 inhibitors.

However, these are balanced by a few concerns. One issue though is that all of the chemistry described in Figs. 1-3 only minimally contribute to the conclusion of the paper, making the paper harder to read for a broad scientific audience.

AR: We understand the concern about the focus on chemical biology in the first part of the manuscript, but we think that our systematic approach was not only important for identification of initial hit-compounds (and ruling out of inactive compouds) but also for exploration of the ligand-enzyme interaction (SAR), which ultimatly guided our inhibitor design.

However, my major concerns are with the virus infection data. It is very clear that these compounds do not just target Mac1, but likely also the polymerase, as the authors even postulated in the discussion. Additional assays are needed to show specificity and provide a more rigorous repression of viral replication. However, it is not imperative clinically that one compound targets just one viral protein. A dual polymerase-Mac1 inhibitor may be more desirable in the clinic than just a Mac1 inhibitor, so the authors may want to think about how they frame the activity of these compounds.

AR: We share the concern of Reviewer 1 regarding additional effects of our Mac1 inhibitors on the viral RNA-dependent RNA polymerase (RdRp). Thus, we now provide virus titration assay data (Supplementary Fig. 20c), which prove strong inhibition of rWT but not the Mac1 mutant rF. We belive that this provides further evidence that our compound primariliy targets Mac1 rather than acting through non-specific mechanism.

In an additional supplementary experiment we therefore investigated the possibility that β-methyl-GS-441524 diphosphate **4** and ST161, β-ethyl-phosphonate phosphate GS-441524 **42** had an additional effect as substrates and/or inhibitors of the minimal replication/transcription complex (RTC) of SARS CoV-2 consisting of nsp12, nsp7 and nsp8. We tested potential incorporation and inhibition of the two compounds ß-methyl-GS-441524 diphosphate **4** and ST161, β-ethyl-phosphonate phosphate GS-441524 **42** by the RTC (Supplementary figure 19). Single-incorporation experiments (left side) show that SARS-CoV-2 RTC was able to weakly incorporate both diphosphate compounds as ATP analogs. Quantification of primer versus

product bands showed that incorporation efficiency after 10 min was 14% and 7%, respectively, in comparison to ATP incorporation using the same concentration of 50 µM whereas the efficiency of the triphosphate of GS-441524 **43** incorporation was 68% (comparison of reactions 1, 3, 4 and 6). When the compounds were used in a NTP mixture at the same concentration (50 µM, right side of the gel) no sign of incorporation of the compounds was detected in contrast to the control GS-441524 triphosphate (compare reactions 2, 8, 9 and 11). GS-441524 triphosphate **43** incorporation (reaction 8) clearly changes the product pattern and shows multiple product bands stemming from competitive incorporation of ATP and GS-441524 triphosphate **43**. Finally, comparative quantification of primer versus products bands showed no sign of inhibition. The primer was elongated with the same efficiency even when 250 µM of compound was present versus 50 µM ATP (reactions 10 and 12). In conclusion, compounds β-methyl-GS-441524 diphosphate **4** and ST161, β-ethyl-phosphonate phosphate GS-441524 **42** do not have a direct dual activity on Mac1 and the RTC of SARS CoV-2. Indirect but limited dual activity may be present via residual degradation to GS-441524 monophosphate **40** and subsequent phosphorylation of GS-441524 monophosphate **40** by cellular kinases as suggested in the manuscript.

We added the following paragraph to our results section of the manuscript: "Full inhibition of replication of rWT and rF by both prodrugs **5** and **6** indicates that the active metabolites (β-methyl-GS-441524-DP **4** and ST161 **42**) have an additional mode of action. Due to the structural relationship to the nucleoside core of Remdesivir, we suspected that the viral RNA-dependent RNA polymerase (RdRp) could be an additional target of Mac1 inhibitors. We thus assessed if compounds **4** and **42** are substrates and/or inhibitors of the minimal replication/transcription complex (RTC) of SARS CoV-2 *in vitro*. (Supplementary Fig. 19) Primer elongation analysis shows that SARS CoV-2 RTC weakly incorporated both compounds **4** and **42**. However, when the compounds **4** and **42** were part of a NTP mixture, we observed neither incorporation of the compounds nor inhibitory effects (up to 250 µM) in contrast to the Remdesivir-metabolite GS-41524-TP **43.** This shows that the Mac1 inhibitors themselves did not directly affect RTC function."

Major Comments

1. Fig. 6a-g: 4 and 42 don't just target Mac1. A complete deletion of Mac1 only has modest 2-3 fold differences in Calu-3 cells (Alhammad et al. 2023), yet these inhibitors inhibited WT and rF mutant viruses almost completely at concentrations less than 1 uM. Furthermore, considering the EC50 values that they determined are often lower than the IC50 values, it is unclear how much the inhibition of Mac1 in infection contributes to the reduced viral replication, as opposed to inhibiting another viral protein.

AR: We thank the reviewer for raising this important concern regarding target specificity, which we acknowledge as a valid and critical point that requires careful consideration. However, in our experiments, rWT exhibits greater sensitivity to our prodrugs compared to rF carrying the catalytically inactive F132A Mac1 mutation. This differential sensitivity suggests that Mac1 function contributes meaningfully to the observed antiviral effects, though we acknowledge that additional off-target activities cannot be entirely excluded.

The observation that cellular EC_{50} values are lower than biochemical IC_{50} values is characteristic of esterified prodrugs. Following cellular uptake, intracellular esterase-mediated unmasking yields charged species that become effectively trapped within cells, resulting in significant intracellular accumulation. This mechanism has been reported to achieve intracellular concentrations up to ~50-fold higher than extracellular levels (reviewed in Vatandaşlar, IJMS, 2022). Consistent with this, our HPLC-based uptake data for ST135/166 (Fig. 7) demonstrate that incubation with 30 nM prodrug yields intracellular concentrations of ~0.3–0.4 µM (assuming CaLu-3 cell volumes from Min et al., Arch Pharm Res, 2016), which are compatible with Mac1 inhibition by β-methyl-GS-441524 diphosphate 4 and approach the measured K_D of ST161 for Mac1 (0.86 µM).

While our Mac1 inhibitors do not directly inhibit viral RdRP at physiological NTP concentrations, we cannot exclude that partial prodrug hydrolysis to GS-441524 monophosphate, followed by cellular phosphorylation, may contribute additional antiviral effects through nucleoside analog mechanisms at higher concentrations.

What is needed, is clear data showing the difference in WT inhibition between untreated and IFN-γ treated cells. As IFN-γ represses Mac1 mutant virus replication, it is also expected that a Mac1-targeting compound would be more effective in the presence of IFN-γ. This does not look to be the case, at least by eyeballing the data. The inhibition of the mutant virus, while supportive, is also complicated by the fact that replication is already dramatically reduced in the presence of IFN-γ (Fig. S16), so it may not be surprising that the inhibitors do not have as great an effect on this virus.

In addition, raw luciferase values (like those in Fig. S16) should be shown in the main figures. It is difficult to interpret relative values when the viruses are at different starting points, for instance, rWT vs rF in the presence of IFN-γ. The authors could at least put one representative figure if luciferase values are highly variable between experiments.

AR: We thank the reviewer for these helpful and important comments. To address these concerns, we added new Supplementary Fig 20a,b showing the raw RLU values from the experiments used to generate the concentration–response curves in former Fig. 6 e and f (now Fig. 8e,f.

In CaLu-3 cells, IFN-γ pretreatment caused a pronounced 7.4-fold reduction of rWT replication, consistent with prior reports (Alhammad et al., PNAS, 2023, Taha et al., PLoS Pathog, 2023). Infection with the catalytically inactive Mac1 mutant (rF) showed an even stronger IFN-γ–mediated suppression (17.4-fold reduction), supporting the conclusion that Mac1 catalytic activity counteracts the IFN-γ–induced antiviral response.

In A549-A/T cells, IFN-γ pretreatment resulted in a smaller reduction of rWT replication (2.1-fold), which was even smaller and did not reach statistical significance in the concentration–response experiments (n = 3). In contrast, infection with rF showed a robust and significant IFN-γ–dependent reduction in luciferase activity (13.2-fold in Supplementary Figure 20; 11.3-fold in concentration–response experiments), indicating that Mac1's catalytic activity also substantially contributes to antagonizing IFN-γ responses in the A549-A/T cells.

Importantly, while ST166 inhibited rWT in the absence of IFN-γ at 0.1 µM and in the presence of IFN-γ already at 0.05 µM, inhibition of rF in the absence of IFN-γ starts at higher concentrations than inhibition of rWT (0.3 µM and greater) and in the presence of IFN-γ ST166 did not further reduce replication of rF up to 1.0 µM, consistent with genetic inactivation of Mac1 and pharmacological inhibition by ST166 targeting the same functional activity within this concentration range. At higher concentrations, ST166 further reduced chemiluminescence in IFN-γ–pretreated cells infected with rF, suggesting an additional Mac1-independent antiviral mechanism, as discussed above.

Furthermore, an additional virus replication assay, preferably a plaque assay, needs to be done to show the effect on infectious virus. Luciferase levels are often very sensitive and do not necessarily correlate to infectious virus, which is the standard in the field.

AR: We acknowledge that luciferase readouts can be highly sensitive and sometimes variable (which is why we originally normalized these values), and thank the reviewer for this valuable methodological suggestion. In addition to including raw luciferase values in the new Supplementary Fig. 20a,b, which now allows clearer interpretation of absolute reporter levels correlating to viral replication levels under different treatment conditions including IFN-γ, we have performed plaque reduction assays to directly measure infectious virus titers (new Supplementary Fig. 20c). These plaque assay data (Supplementary Fig. 20c) demonstrate that the inhibitory effect observed is indeed reflected by reduced infectious virus titers of rWT in presence of our compound.

Again, perhaps the authors could demonstrate that these compounds target both polymerase and Mac1 which could be a more interesting and possibly more useful outcome.

AR: We thank the reviewer for confirming us in our belief that a compound with such a dual mode of action, inhibiting both Mac1 and the viral RNA polymerase (by prodrug breakdown as described above) is interesting and useful.

We included this concept in discussion, stating that: "Notably, rF was not completely resistant, suggesting the presence of a secondary, Mac1-independent mode of action of prodrugs **5** and **6**. Given the remdesivir-derived core motif present in both compounds, a plausible additional viral target is the viral RNA-dependent RNA polymerase (RdRp). Although the active metabolites **4** and **42** do not substantially interfere with replication-transcription complex (RTC) function under physiological conditions, partial destabilization of the pyrophosphate moiety by the masking group in prodrug 5 could promote formation of GS-441524-MP **40** upon intracellular processing. Subsequent phosphorylation to the corresponding triphosphate **43** could, in principle, inhibit the viral RdRp[68], analogous to the mechanism of remdesivir. Consistent, with this interpretation, stabilization of the pyrophosphate in compound **6** increased the differential inhibition observed between rWT and rF inhibition, indicating a shift toward preferential Mac1 targeting. This interpretation is further supported by the observation that ST166 **6** restores IFN-γ-induced NAD^+ depletion during SARS-CoV-2 infection, consistent with the effective inhibition of Mac1-dependen ADPR turnover. While the contribution of polymerase inhibition remains to be formally established, a a dual mode of action maybe advantageous from a therapeutic perspective, as resistance development would likely require concurrent adapation in two independent viral targets."

2. Fig. 6h-i: This IFN data could provide important specificity data, but it is somewhat perplexing. While the ISG data does suggest inhibition of Mac1 as the inhibitor enhancing their abundance, the IFN data does not, even though it has been well documented that Mac1- mutant viruses lead to enhanced IFN, as is shown here with the F132A virus. One problem may be in the design of the experiment. The infection was done at an MOI of 1 and harvested at 48 hpi, a time point at which the infection should be largely over. It may be beneficial to do the experiment at either a lower MOI or collect at 24 hpi.

AR: We thank the reviewer for this constructive criticism regarding the experimental design and interpretation of our data. We acknowledge that the apparent discrepancy between ISG and IFN induction was indeed puzzling and required further investigation. Following the reviewer's suggestions, we have repeated these experiments under modified conditions. In our revised experiments, we now observe significant IFITM1 induction by our Mac1 inhibitors in the absence of exogenous IFN-γ treatment (new Figure 8), which provides clearer evidence for Mac1-specific effects and is more consistent with the expected phenotype of Mac1 inhibition. This supports our hypothesis that our compounds effectively target Mac1 function and thereby restore the cellular antiviral response.

We also investigated the reviewer's suggestion to examine earlier time points. While we explored the 24 hpi time point as recommended, we found insufficient ISG induction (<10-fold) in our experimental system at this earlier time point to provide meaningful data (data not shown). The 48 hpi time point, while later in the infection cycle, appears to be optimal for detecting the ISG responses in our cellular model, likely reflecting the kinetics of Mac1-mediated immune suppression and its reversal upon inhibitor treatment.

In addition, it's unclear why in figure 6h and 6i that the authors did not include a condition where ST166 is added without IFN. Furthermore, the pretreatment with IFN only complicates the analysis of IFNs and also the ISGs. The rF virus has an increase in IFN and IFITM1 without IFN (which was also previously published for a Mac1 deletion virus (Alhammad et al. 2023)), so it unnecessary to do this pretreatment. The authors just need to demonstrate that ST166 increases IFN/IFITM1 levels in a WT infection, but not the mutant. If they can do this, that will show the specificity needed for Mac1 during infection even if it's clear the compounds are targeting multiple viral proteins based on the replication assays.

AR: As mentioned in our response above, we have revised these experiments following the reviewer's suggestions regarding experimental design and timing. We would like to further address the specific points raised regarding the experimental conditions: The reviewer was absolutely correct that IFN-γ pretreatment unnecessarily complicated the analysis. We have now focused on the condition of ST166 treatment without IFN-γ pretreatment (new Figure 8) and we agree that this simplified approach allows for clearer and easier interpretation. We now demonstrate that ST166 increases IFITM1 levels during rWT infection, which adds to our assumption of Mac1 specificity during infection.

We have added to the text accordingly (new lines 446-456: „Next, we analyzed the induction of IFN-β and IFITM1 as representative ISGs (Fig. 8h). Both viruses induced IFN-β expression; however, transcript levels were significantly higher in rF-infected cells than in rWT-infected cells (404-fold vs. 200-fold). Treatment with ST166 **6** increased IFN-β induction in rWT-infected cells to levels comparable to those observed for rF (299-fold vs. 227-fold).

Induction of IFITM1 showed an even more pronounced difference between the viruses, with rF triggering substantially higher expression than rWT (1435-fold vs. 322-fold). Inhibition of Mac1 by ST166 **6** markedly enhanced IFITM1 induction in rWT-infected cells (1014-fold), elevating expression to levels similar to those induced by rF (1326-fold). In contrast, ST166 **6** had no significant effect on IFITM1 induction in rF-infected cells. Consistent trends were observed under conditions of IFN-γ pretreatment (Supplementary Fig. 22). In this setting, ST166 **6** significantly increased IFITM1 induction in rWT-infected cells, whereas ISG induction in rF-infected cells remained unaffected."

Another option to clearly show specificity would be to identify drug-resistant mutations in Mac1 or do a CETSA assay (though tricky for an infected cell).

AR: We agree with the reviewer that such a target engagement assay would be convincing, but we currently have no possibility to do this in an infection setup. We hope that the experiments and data described above can demonstrate the specificity of our compounds convincingly.

3. Fig. 1-3: The paper could have easily started at Fig. 4 and very little would be different. Furthermore, it is already known that GS-441524 binds and inhibits Mac1 (though weakly) (Tsika et al. 2022; Ni et al. 2021), so again the manuscript could have started at that spot, though I understand that some of the things that were learned in Figs. 1-3 contributed to the newer molecules that were highly effective, but it was only a few things. This may be okay for a chemistry journal but I'm not sure it will be okay in a broader interest journal.

AR: As noted above, and as acknowledged by the reviewer, the experiments presented in Figures 1–3 provided critical insights that directly informed our drug design strategy. While some of the tested compounds did not yield further optimization opportunities, these results are nonetheless an integral and nontrivial part of the structure–activity relationship (SAR) analysis.

We believe that presenting the full scope of the SAR—including less successful analogs—is important for transparency and for conveying the rationale underlying subsequent compound design. In our view, this information is of interest even to a broader readership, as it illustrates key chemical–biological constraints of the ADP(R) scaffold rather than only final optimized outcomes. This perspective is also supported by the other reviewers, who suggested that additional chemical biology data be moved from the Supplementary Information into the main text.

For these reasons, we feel that retaining Figures 1–3 in the main manuscript provides important context and strengthens the overall narrative of the study.

Minor Comments:

1. Fig. 6h,i: Please indicate which IFN is used for pretreatment. I would assume its IFN-γ but this should be indicated in the figure legend and on the figure. In addition, it would be helpful to state the concentration of ST166 in the legend so the reader doesn't have to go the Methods section for this information.

AR: The IFN used is indeed IFN-gamma (IFN-γ). We changed the figures and legends accordingly.(Fig 8, Supplementary Figs 20 and 22)

2. Title: As only 1 of the inhibitors throughout the study demonstrated a nanomolar level of inhibition, the title should read "a nanomolar inhibitor" or "novel inhibitors".

AR: We agree and changed the title according to the reviewer´s suggestion to "**Targeting a viral macrodomain: Design, Structure-Based Optimization and Antiviral Evaluation of Potent Inhibitors for Mac1 of SARS-CoV-2**"

3. Line 252: Tsika et al. previously published that GS-441524 inhibits Mac1 activity, thus it should be cited here and likely in other places.

AR: We thank reviewer 1 and added the citation in line 264 (former 252) accordingly.

4. Discussion: While it's not necessary to highlight a bunch of publications that have identified Mac1 inhibitors, the discussion should put this work in the context of additional efforts to identify Mac1 inhibitors and at least reference recent manuscripts that have identified Mac1 targeting inhibitors that also repress virus replication (Wazir et al. 2024; Pfannenstiel et al. 2025, Suryawanshi et al. 2024).

We agree and added the following paragraph to the discussion: "While nucleoside and nucleotide derivatives have been used to develop antiviral drugs, efforts to develop Mac1 inhibitors focused mostly on identifying non-nucleotide small molecules. Notable achievements have been made by other groups, which yielded Mac1 inhibitors that also showed effect in viral infection assays[18–20].

For instance, following a biochemical HTS of 30,400 compounds, Wazir et al. identified a 2-amide-3-methylester thiophene scaffold that led to the synthesis of MDOLL-0229 with an IC_{50} of 2.1 µM and a reported EC_{50} between 12.5 and 25 µM in SARS-CoV-2 infected CaLu-3 cells[18].

In addition, Pfannenstiel et al. conducted *in silico*-assisted structure-based optimization of a tryptophanate precursor MCD-628 [58]and generated two membrane-permeable antivirals (compound 5c and 6e) with an IC_{50} of 4.9 and 3.7 µM respectively, which both inhibited SARS-CoV-2 replication in CaLu-3 cells at 12.5 µM [19].

Furthermore, capitalizing on previously described Mac1 inhibitor candidates [16], Suryawanshi et al. performed an extensive medicinal chemistry campaign to merge key structural elements of AVI-92 and AVI-219, which yielded the novel AVI-4206, a pyrrolidinone with additional urea-functionality exhibiting an IC_{50} of 64 nM while impairing SARS-CoV-2 replication in human airway organoids at 4 µM [20]."

Reviewer #2:

The manuscript describes a substantial body of work related to the discovery of potent and specific inhibitors of the Mac1 SARS-CoV-2 viral microdomain, a macrodomain known to counter ADP-ribosylation based immune response. The authors develop robust chemical

phosphorus chemistry to generate monophosphates, diphosphates, phosphophoponates and masked derivatives to facilitate the transition from in vitro enzymatic inhibition studies to cell-based assay. Through a very systematic approach, the authors have developped a robust SAR using a combination of isothermal measurements, in silico analyses, and protein crystallography. The development of membrane permeable phosphorylated derivatives was also very well thought out and conmprehensively demonstrated that the newly generated nucleotides were active against viral infection by targetting Mac1 specifically and with limited off-target activity.

AR: We thank the reviewer for the positive comments and the acknowledgement of our efforts to conduct a systematic, comprehensive SAR and our commitment to investigatate the specificity towards Mac1.

However, the mild cell cytotoxicity observed should be evaluated more thoroughly to establish whether the toxicity is due to the quinone being released during the intracellular release of the phosphorylated inhibitors, or otherwise. A masking-agent containing control molecule should be used in cell based assays.

AR: We thank the reviewer for raising this important point regarding the origin of the observed cytotoxicity. In our own cellular MTS assays, the C11-AB–masked prodrugs evaluated in this study exhibited limited cytotoxicity in CaLu-3 cells: ST135 showed a CC_{50} value above the highest concentrations tested (> 50 µM), while ST166 displayed no detectable cytotoxicity over the concentration range assessed, despite potent antiviral activity. These data indicate a favorable selectivity window and do not suggest pronounced intrinsic toxicity of the prodrug constructs.

Importantly, differences in CC_{50} values between parent nucleosides and their corresponding Di*PP*ro prodrugs cannot be directly interpreted as evidence for toxicity arising from the masking group or its stable cleavage products (i.e. 4-hydroxy-benzylalcohol, see Figure below).

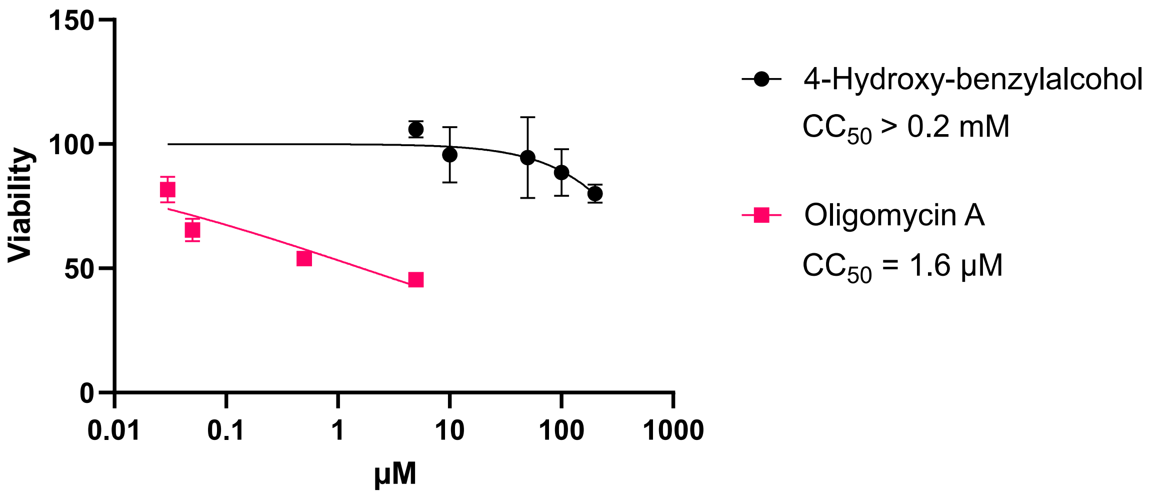

Figure 1: MTS Assay for Calu-3 cells treated with 4-Hydroxy-benzylalcohol or Oligomycin A.

Di*PP*ro prodrugs are designed to enhance membrane permeability and intracellular delivery of the active nucleotide species; thus, increased intracellular exposure can unmask pharmacological or downstream cytotoxic effects that remain undetectable for poorly permeable parent compounds. In this context, CC_{50} values primarily reflect cellular exposure and biological activity, rather than intrinsic toxicity of the prodrug moiety

Additional support for this interpretation comes from extensive studies on di- and triphosphate prodrugs of antivirally active nucleoside analogues employing chemically identical AB masking groups. In these systems, all prodrugs were shown to undergo efficient hydrolysis in buffer and in cell extracts, yielding identical masking-group cleavage products. Nevertheless, cytotoxicity was observed only for compounds displaying antiviral activity, whereas prodrugs lacking antiviral activity showed no detectable toxicity despite confirmed hydrolysis and release of the same masking-group fragments (Jia and Meier, *J. Med. Chem.* 2023; *Adv. Sci.* 2023; *J. Med. Chem.* 2020). If cytotoxicity were predominantly driven by the masking group or its breakdown products, comparable toxicity would be expected across all such compounds, which is clearly not the case.

Taken together, these observations indicate that the limited cytotoxicity observed for some Di*PP*ro prodrugs is not attributable to the stable phenolic cleavage product and cannot be explained by masking-group effects alone. While transient electrophilic intermediates formed during prodrug activation (e.g., p-quinone methides) may contribute modestly, the overall cytotoxicity profile is more consistent with compound-specific intracellular exposure and biological activity.

It is surprising that the authors did not probe for A(P)DPR or amino modification like N6-methylation (very different effect on the nuclobase pKa).

AR: We thank the reviewer for these suggestions. Regarding 2′-phospho-ADPR (ADPRP), we have already performed these experiments, and the results are shown in Fig. 2a and Fig. 2b. Both the pIC_{50} (4.43 ± 0.19) and the affinity (K_d = 35.2 µM) were lower than for ADPR. Literature data indicate that the pKa of the adenine base in adenosine 2′-phosphate is only slightly higher than that of adenosine itself (pKa = 3.74 and 3.63, respectively; Z. Shabarova, A. Bogdanov, *Advanced Organic Chemistry of Nucleic Acids*, VCH, 1994), the shift being far smaller than for 2-fluoro-adenosine or 7-deaza-adenosine (pKa < 1 and pKa = 5.2, respectively; Suydam & Sobel, JACS 2008).

As for N6-alkylated derivatives, we tested the commercially available 6-(1-methyl-butyl)-ADP in comparison to ADP and observed no significant difference in pIC_{50} (2.1 ± 0.2 vs. 2.3 ± 0.1, see Figure below). Interpretation of this result is less straightforward due to competing effects. Alkylation at N6 increases the basicity of N1 (for example, N6-methyladenosine shows an experimentally determined pKa for N1 of 4.01; Martin & Reese, J. Chem. Soc. C 1968), which would be expected to strengthen its function as a hydrogen-bond acceptor. At the same time, N6 alkylation results in *syn* and *anti* rotamers of the exocyclic amine that rapidly interconvert. Steric clash between the alkyl group and N7 strongly favors the *syn* rotamer, with solution NMR showing *syn*/*anti* ratios greater than 10 (Liu *et al.*, Nat. Commun. 2021). In the *syn* orientation, the N6-H is unfavorably oriented for forming a productive hydrogen bond with Asp22 of Mac1, and even in the *anti* orientation the secondary amine is a weaker hydrogen-bond donor than the primary amine in adenosine due to reduced polarization of the N–H bond. These data indicate

that although amino-group modifications at N6 are in principle expected to affect hydrogen bonding, the net effect on Mac1 binding is mitigated by competing electronic and conformational factors, explaining why no pronounced change in affinity is observed.

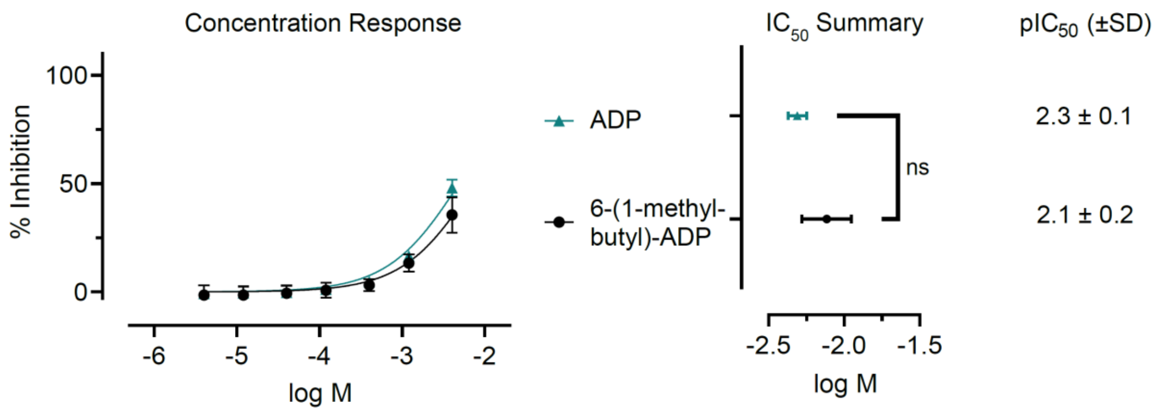

Figure 2: Concentration responses of ADP and 6-(1-methyl-butyl)-ADP on Mac1 activity determined by plate assay.

The authors have done an excellent job at describing the syntheses developed. There are however occasions where the number of mole of product generated being reported cannot correlate to the number of mole of starting material used. Surely there is something odd there. The % yields should have been determined using UV (adenosine 256nm) for quantification and confirmation of the reaction efficacy.

AR: We appreciate the reviewer´s acknowledgment of our detailed synthesis part. Regarding the product yields, we would like to emphasize that chemical yields of chemical reactions are generally reported as % yields referring to the mol amount of starting material. This is how we have done it her in our manuscript as well. In our opinion the calculation done on the basis of UV absorbance is much less accurate.

Finally, the scalability of the reported syntheses is of concerns considering the scale at which the reactions were conducted since less than 20mg of starting materials were used for most synthetic conversions. This is unfortunate.

AR: We thank the reviewer for mentioning this. At this point of time of the development of the target molecules and prodrug we limited ourselves to amounts of about 10-30 mg. With such amounts we were able to get first property studies done. For now we have not scaled-up the reaction to higher amounts. Nevetheless in other studies from our laboratory we already prepared Di*PP*Pro- or Tri*PPP*ro-compounds in up to 200 to 500 mg without encountering particular problems.

In the reported syntheses, it is imperative that the form of the phosphate reagents is clearly stated: salt + counter cation or free acid form.

AR: We agree with the reviewer. In all cases the compounds were isolated as salt forms with triethylammonium or ammonium counterions. Free acid forms are often very hygroscopic and thus difficult to handle and to store.

The stability study and the release rates should have been carried out in cell culture media and not just in NMR solvents.

AR: To address the stability in cell culture medium we repeated stability assays for ST166 in RPMI and, additionaly in phosphate buffered saline (PBS). The results have been added as new Supplementary Figures 15 and 16 and show that ST166 has a $t_{1/2}$ of ~45h in cell culture medium and a $t_{1/2}$ of ~71h in PBS.

Some of the reported chemicals and reagents are known and published entities for which a literature reference should be provided.

AR: We agree and made sure to reference them in the manuscript accordingly.

The NAD levels in cells post infection should have been measured with and without the membrane permeable inhibitor, with or witoht iFN induction. The effect of Mac1 inhibition could have very unexpected effects on the cellular NAD levels: Inhibition of Mac1 might promote NAD depletion through unchecked cellular ADPR ribosylation or alternatively or stabilize intracellular NAD levels.

AR: We thank the reviewer for this important suggestion. We have now quantified cellular NAD levels in CaLu-3 cells under all relevant conditions (±ST166, ±IFN-γ, ±SARS-CoV-2 infection) using an established enzymatic cycling assay, and have added these data as Supplementary Figure 21.

As a positive control, we included the NAMPT inhibitor FK866. Consistent with previous reports, FK866 showed no measurable effect after 2 h but reduced cellular NAD levels by ~90% after 48 h (Hasmann et al., Cancer Res 2003; Lin et al., Cell Reports 2016), validating the assay sensitivity.

In uninfected cells, IFN-γ treatment resulted in an approximately two-fold reduction in NAD levels after 2 h, consistent with induction of interferon-stimulated genes and activation of NAD-consuming enzymes such as PARPs (Heer et al. JBC 2020). At 48 h, NAD levels recovered to near-baseline, in agreement with increased activity of the NAD salvage pathway reported previously (Heer et al. JBC 2020).

Treatment with ST166 alone (0.3 µM; 10 × EC_{50}) did not significantly alter cellular NAD levels at either time point, in contrast to our uptake assays for which we employed substantially higher prodrug concentrations (0.5–50 µM).

Importantly, SARS-CoV-2 infection did not significantly affect NAD levels at 2 h post infection, and only a minor reduction was observed at 48 h. Co-treatment with ST166 did not further alter NAD levels in infected cells. Moreover, IFN-γ pretreatment of infected cells resulted in a less pronounced NAD reduction than in uninfected cells, consistent with the reported antagonistic role of Mac1 in interferon signaling and ISG expression (Taha et al., PLOS Pathogens 2023; Alhammad et al., PNAS 2023).

Finally, combined treatment of infected cells with IFN-γ and ST166 reduced NAD levels to those observed in IFN-γ–treated uninfected cells, in line with previous reports describing IFN-γ–mediated sensitization to Mac1-targeted inhibition.

Taken together, these results demonstrate that pharmacological Mac1 inhibition does not induce unexpected depletion or stabilization of cellular NAD pools under the conditions tested, addressing the reviewer's concern directly.

Reviewer #3:

The authors describe an optimization of a previously characterized Mac1 inhibitor GS-441524 to a cell active prodrug. Initial view is that the manuscript is a nice medicinal chemistry optimization of the compound. However the manuscript appear as two studies put together.

The first part on the ADP/ADPr analogs is a careful study on how these analogs could bind to the Mac1. This does not appear like a very new idea as ADP-ribose has been tried as a starting point to find macrodomain binding compounds before. The work has clear merits as it is carefully conducted and complemented with co-crystal structures of many analogs. The changes in potency of the compounds are mostly detrimental or show what modifications are tolerated.

AR: We thank the reviewer for the positive comments and the detailed and constructive review.

This however appears not to be connnected to the second part which the only study mentioned in the abstract.

AR: To better reflect the SAR part of the manuscript and its connection to the drug design, we adjusted our abstract.

The first part of the abstract now reads: "We tested ADP / ADP-ribose analogues for their ability to inhibit Mac1 of SARS-CoV-2, measured affinity of active compounds and determined their binding mode by cocrystallization, uncovering critical molecular determinants of protein-ligand interaction. Key findings of the resulting structure-activity relationship (SAR) included that inhibitory potency was improved by either replacing the distal ribose of ADP-ribose by a small alkyl group or the adenine N7 by carbon. Insights from the SAR guided our design of β-methyl-GS-441524-diphosphate, a nanomolar inhibitor that exhibits >1000-fold selectivity over human MacroD1 and MacroD2. "

In the second study the authors make small modifications to a previously discovered compounds which has been already been used to create nanomolar fluorescent probes etc. (PMID: 37126856) The value and the main interest in the manuscript is the modification of GS-441524 that made it potent, cell permeable and that has some antiviral activity.

AR: We thank the reviewer for the positive remarks.

Some major points

In the introduction the authors state that most of the inhibitors have been discovered by virtual screening. This statement is not correct as most of the validated inhibitors have been discovered by a range of experimental techniques including HTS based on competition and activity assays, fragment screening in crystallo etc.

AR: We thank the reviewer for pointing this out and agree that our original wording did not adequately reflect the diversity of experimental approaches that have been used to identify validated inhibitors. We have expanded the Discussion to explicitly acknowledge prior work employing HTS competition and activity assays, fragment screening (including *in crystallo* approaches), and other experimental strategies. These additions now provide a more balanced and accurate overview of the field and better place our work in the context of previous studies.

This section now reads:" While nucleoside and nucleotide derivatives have been used to develop antiviral drugs, efforts to develop Mac1 inhibitors focused mostly on identifying non-nucleotide small molecules. Notable achievements have been made by other groups, which yielded Mac1 inhibitors that also showed effect in viral infection assays[18–20].

For instance, following a biochemical HTS of 30,400 compounds, Wazir et al. identified a 2-amide-3-methylester thiophene scaffold that informed the synthesis of MDOLL-0229 with an IC_{50} of 2.1 µM and a reported EC_{50} between 12.5 and 25 µM in SARS-CoV-2 infected CaLu-3 cells[18].

In addition, Pfannenstiel et al. conducted *in silico*-assisted structure-based optimization of a tryptophanate precursor MCD-628 [58]and generated two membrane-permeable antivirals (compound 5c and 6e) with an IC_{50} of 4.9 and 3.7 µM respectively, which both inhibited SARS-CoV-2 replication in CaLu-3 cells at 12.5 µM [19].

Furthermore, capitalizing on previously described Mac1 inhibitor candidates [16], Suryawanshi et al. performed an extensive medicinal chemistry campaign to merge key structural elements of AVI-92 and AVI-219, which yielded the novel AVI-4206, a pyrrolidinone with additional urea-functionality exhibiting an IC_{50} of 64 nM while impairing SARS-CoV-2 replication in human airway organoids at 4 µM [20]."

Also the statement that the approach used here would be completely different would not be true. Throughout the text they also refer to studies where ADPr analogs have been tested for Mac1 inhibition.

AR: We thank the reviewer for making us aware of the awkward wording. We rephrased the paragraph in the discussion to better reflect the commonalities and differences to the studies mentioned above: "We, in contrast, used an assorted, purine-targeted library that includes many ADP(R) analogs not commercially available and probed the relative importance of the different structural elements of ADPR **1**, which resulted in the first initial complete SAR and allowed us to come up with a nucleotide-based inhibitor of Mac1."

In addition, we changed the paragraph in the introduction to: "Numerous attempts have been made to identify inhibitors for the viral macrodomain, e.g. by high-throughput screening (HTS)

and fragment screening, but so far only few viable drug candidates for Mac1 have been identified[11–20].

Here, using an assay for the enzymatic activity of Mac1, we probed its active site using a unique collection of ADPR and ADP derivatives. The resulting initial SAR guided our modification of the low-affinity Mac1-binding nucleoside GS-441524 **3**[21], into a nanomolar inhibitor: β–methyl-GS-441524-diphosphate (β–methyl-GS-441524-DP) **4**.".

The virus assays would be more robust if there would be target engagement study included in the work.

AR: We agree with the reviewer that a direct target engagement assay would provide valuable additional support for the proposed mechanism of action. However, such assays require specialized tools and experimental setups that are currently not available in our laboratories. To address this point as far as possible within these constraints, we have performed additional infection assays, i.e. analysis of IFN-ß and IFITM1 induction (Fig. 8), quantification of cellular NAD-levels as well as viral titration assays (Supplementtray Figs 20c, 21). We believe that the consistent antiviral effects observed across these assays provide strong indirect evidence for target engagement and support the activity of our compounds.

The work with the catalytically inactive Mac1 should be explained better as why to target Mac1 if the inactivating mutation does not inactivate. Authors imply that there could be other mechanism for the prodrug, even a different target.

AR: We thank the reviewer for the opportunity to clarify the rationale for the choice of the F132A Mac1 mutant. This mutant was selected to probe the specificity of our Mac1 inhibitors, rather than to phenocopy a complete loss of Mac1 function. Our working hypothesis was that the de-ADP-ribosylating (hydrolase) activity of Mac1 contributes to its ability to interfere with host innate immune responses, and that inhibition of this activity would become more apparent under conditions of immune activation.

The absence of a strong phenotype for the F132A mutant under basal conditions is consistent with previous reports showing that genetic perturbation of Mac1 has only a limited impact on SARS-CoV-2 replication in CaLu-3 cells. In particular, complete deletion of Mac1 was reported to have a modest effect in these cells in the absence of exogenous interferon signaling (Alhammad et al., PNAS 2023). Likewise, for the catalytically inactive but ADP-ribose–binding–competent N40D mutant, no substantial replication defect was observed in CaLu-3 cells in the absence of interferon stimulation (Taha et al., PLOS Pathogens 2023), despite clear inactivation of the de-ADP-ribosylating activity of Mac1 (O'Connor et al., J. Virol. 2025).

In line with these observations, the F132A mutation alone had little effect on viral replication in CaLu-3 and even less in A549-A/T cells (Supplementary Fig. 18). Nevertheless, we included this mutant to assess the contribution of Mac1 catalytic activity to the antiviral effects observed with our compounds.

Importantly, upon stimulation with IFN-γ, we observed a clear shift in the concentration–response curve for the F132A mutant relative to the wild-type virus. This differential response indicates that part of the compound activity is linked to the catalytic function of Mac1.

Thus, we changed the paragraph in the results section to:" To study the effect of inhibitors on SARS-CoV-2, we constructed recombinant SARS-CoV-2 encoding a luciferase reporter and either wild-type Mac1 (rWT) or catalytically inactive Mac1 rF132A (rF) to assess the contribution of Mac1 catalytic activity to the antiviral effects observed with our compounds. (Fig. 8a)."

The mutation likely affects compound binding potency and this is not tested.

AR: To address this point we performed additional ITC measurements (Supplementary Fig.17). The F132A mutant exhibits an affinity of 30 nM for ß-methyl-GS-441524-diphosphate and 540 nM for ST161, indicating that the mutation increases the affinityof the macrodomain for both compounds.

In addition we added following passage to the results section: "To test if the Mac1 mutant binds the active metabolites (compound **4** and **42**) of our prodrugs (**5** and **6**) differently than the wild type, we determined binding affinities. As expected, Phe132Ala Mac1 binds to both compounds **4** and **42**, albeit with somewhat higher affinities (0.03 and 0.54 µM respectively, Supplementary Fig. 17).".

Some minor points

Figure 1c is not especially clear presentation due to the color choices and it maybe better to use only one color for proteins carbons in the figure as now it changes in every picture. Different color for different compounds would highlight the compound more and clearly show the differences. The views are well selected though. It would be easier for the reader to compare b and c if the ADPr would be in a roughly similar orientation and some summary schematic on the effect of the changes could be used in the main text and not just in the supplement.

AR: We appreciate the positive comment on the co-crystal views and are thankful for the suggestions for better visual impression. We changed protein carbon colour to grey (better contrasting the coloured ligands) (Fig.1c, 2e, 3d, 5e, 7e). We agree with the reviewer that reorienting the chemical structure of ADPR would better reflect the binding mode shown in crystallo and that the schematic SAR figure should be more prominent in the manuscript. We thus flipped the structure of ADPR upside down and integrated the former Supplementary Fig. 5 to the set of main figures (new Fig. 4).

It is nice to see the electron density figures in the supplement. In some cases like for compound 29 the density is not super clear for the resolution cut off so it may halp to include the protein residues and waters also. PDB validation reports were not provided.

AR: To address the unclear electron density, we have reprocessed and revised all ligand density figures, including re-alignment of the maps and application of a more stringent and consistent density carving procedure. As a result, the electron density supporting ligand placement is now substantially clearer across all complexes, including compound 29. Given this improvement, we believe that inclusion of nearby protein residues and solvent molecules is no longer required to support ligand interpretation in the electron density figures. Nevertheless, to further enhance transparency and rigor, we now provide the complete PDB validation reports for all structures as separate Supplementary Information accompanying the manuscript as requested.

Prodrug structures should be included in the main manuscript and not just in the supplement.

AR: We agree and moved the former Supplementary Fig. 12 to the set of main figures (new Fig. 6).

In the text it is not clear if the ADPr Mac1 complex was solved again by authors as no reference or comparison to previous structures is given. The structure just appears.

AR: We thank the reviewer for making us aware of this. We indeed did solve the structure again. This paragraph now reads: "Structural superposition of our own high-resolution crystal structure of Mac1 in complex with ADPR **1** (PDB: 8AZD) with existing structures of Mac1-ADPR complexes (PDB: 6Z5T, 7KQP, 6WOJ, 6W02) demonstrates a conserved macrodomain fold and ligand binding mode, with two key hydrogen bonds between the adenine base and the protein: N6 acts as hydrogen bond donor to Asp22, while N1 accepts a hydrogen bond from the amide N-H of the peptide bond between Asp22 and Ile23."

There are some typos and eg. "compunds" in the abstract all the NAD+ are missing the +.

AR: We thank the reviewer for carefully reading our manuscript and directing us to that. We changed this in the manuscript accordingly.

Manuscript: Targeting a viral macrodomain: Design, structure-based optimization and antiviral evaluation of potent inhibitors for Mac1 of SARS-CoV-2

ID: NCOMMS-25-56595A

Response to reviewers

We again thank the reviewers for their constructive comments, which we have all addressed in the second revision of our manuscript. We believe that these comments and our revision have improved the manuscript over the last version. Listed below are our general changes to the manuscript as well as our point-by-point responses to the comments of the reviewers.

General changes in the revised manuscript:

- Corrected capitalization of the title.
- Added additional labels to Fig. 6a to clearly identify ST-135 and ST-166.
- Moved former Supplementary Fig. 20 a and b (raw luciferase data of infection experiment) to the main manuscript as panels h and i of Fig. **8**.
- Added a new Supplementary Fig. 20 showing the effect of remdesivir on luciferase activity of recombinant SARS-CoV-2.
- Added data from a second experiment to the former Supplementary Fig. 20c (Plaque assay) (now Supplementary Fig. 21).
- Added additional data from a repeat experiment to former Supplementary Fig. 21 showing the effect of ST166 on cellular NAD^+ levels (now Supplementary Fig. 22)
- Added a new Supplementary Fig. 23 comparing the viral RNA levels upon infection with recombinant SARS-CoV-2 with or without ST166 treatment.
- Increased the font size on the labels of the y-axis in Fig. 8 h + i
- Some changes in the text to avoid single sentence paragraphs and increase readability.
- Corrected error in Fig. 8d and revised statistics in Fig 8d + 8g and changed legend*

Reviewer #1:

I want to thank the authors for their attention to detail on the comments, and the manuscript has been improved substantially. I would specifically mention that the new IFN/ISG data increased my confidence that the compound targets Mac1 during infection. However, there are still some issues that need to be further addressed, mostly involving interpretations of the results in Fig. S20 and S21. Most are textual changes, but some additional controls and experimental repeats for the current experiments are also needed.

Author response (AR): We thank the reviewer for the very detailed and helpful review and comments.

Comments
1. First, in the title either all terms need to be capitalized, or they all should be lowercase. i.e. viral macrodomain should be Viral Macrodomain based on the way the rest of the title is written.

AR: We thank the reviewer for pointing this out. The author guidelines give no specific recommendation on title capitalization, but looking at recent papers in Nature Communications, the dominant style today appears to be sentence case. We thus revised the title of our manuscript to sentence case.

2. Line 50: Should be active, not activate.

Manuscript: Targeting a viral macrodomain: Design, structure-based optimization and antiviral evaluation of potent inhibitors for Mac1 of SARS-CoV-2

ID: NCOMMS-25-56595A

AR: We agree and changed this to "catalytically active".

3. Lines 64-69. The final sentences of this paragraph are not as to why they transitioned from ST135 (5) to ST166 (6). It should be stated more clearly that compound 5 had reduced stability and specificity in antiviral assays, necessitating the production of compound 6.

AR: We agree with the reviewer that this did not appropriately reflect our reasoning and thus replaced the sentence "Recombinant SARS-CoV-2 with intact or modified Mac1 enabled us to evaluate inhibitor specificity and optimize stability and antiviral potency, culminating in prodrug **6** (ST166)." by: "Recombinant SARS-CoV-2 with intact or modified Mac1 enabled us to evaluate the specificity of the inhibitor. Determination of the *in vitro* stability of compound **5** revealed a short half-life due to spontaneous hydrolysis of the phosphate anhydride bond. Replacement of the terminal methyl phosphate with an ethyl phosphonate, yielding compound **6**, markedly improved stability."

4. Figure 6. From this figure it was unclear exactly which molecule is ST135 (5). This should be labeled.

AR: We thank the reviewer for pointing this out and added additional labels to Fig. 6a to allow for unequivocal identification of ST-135 and ST-166.

5. The IFN/IFITM1 is perhaps the best data to show the specificity, and to do this experiment the authors modified their normal infection protocol to start drug-treatment at 16 hpi with a high MOI to likely ensure that the replication of each virus was only minimally impacted considering they used a higher dose of compound (0.5 uM). These changes to their general procedures should be described in the results, not just in the methods.

AR: We agree with the reviewer that this would be helpful information for the reader. The modified experimental conditions used for this assay, including the addition of ST166 **6** at 16 hpi to allow establishment of infection prior to treatment, are now explicitly described in the Results section.

The section now reads: "Next, we analyzed the induction of IFN-β and IFITM1 as representative ISGs in cells infected with a MOI of 1 of either rWT or rF. To allow infection to become established prior to inhibitor treatment ST166 **6** (0.5 µM) was added 16 hpi (Fig. 8j)."

Also, it would be prudent to include viral RNA levels in this assay to confirm that there were no drastic differences in viral RNA amounts with/without treatment or comparing the mutants.

AR: We agree and have therefore included viral RNA measurements to assess potential differences in viral RNA levels between treatment conditions and viral mutants. The corresponding data have been added to the revised manuscript as Supplementary Fig. 23. In addition, primers and probe used for viral RNA amplification are now referenced in the methods section (Corman et al, new reference 86).

6. Supplemental Figure 20a-b should be included in the main manuscript. These results are critical for the interpretation of the data.

AR: We thank the reviewer for pointing this out and included the raw luciferase data from Supplementary Figure 20a-b now as panel h and i of Figure 8 in the main manuscript.

Also, what is the limit of detection in these assays? A control using an established drug (i.e. GS441524 – inhibits polymerase activity) should be included.

AR: We thank the reviewer for this suggestion. In our assay system, the practical limit of detection is primarily determined by residual luciferase activity associated with the input virus particles, which is

reflected in the 0 hpi control measurements. Consequently, luciferase signals cannot decrease substantially below this background level, irrespective of inhibitor potency.

Importantly, at the highest inhibitor concentrations tested, the measured RLU values already approach the 0 hpi / background signal, indicating that viral replication is suppressed to the detection limit of the assay. Therefore, inclusion of an additional model inhibitor would not further improve definition of assay sensitivity, as the lower boundary is constrained by the intrinsic background signal derived from virus-associated luciferase carryover, nevertheless we determined in an independent experiment the dynamic range using remdesivir as model inhibitor. When cells were treated with RdRp inhibitor, RLU values approached the luminescence at 0 hpi confirming that the background luciferase activity essentially limits the dynamic range of the assay to approx. 3 - 3.5 log steps (Supplementary Fig. 20)

To clarify this point, we have added an explanation of the assay detection limit and the relevance of the 0 hpi control values in the revised manuscript.

The respective section reads: "The dynamic range of the luciferase assay is mostly limited by the residual luciferase present in the input viral particles (indicated in Figs. 8h,i and Supplementary Fig 18 as dashed line denoting 0 hpi) and covers approx. 3.5 log steps. This was confirmed by using 2 µM of the RdRp inhibitor remdesivir to fully inhibit viral replication (Supplementary Fig. 20). "

The graphs themselves should probably be shown on a log-scale to better interpret what is happening at the higher concentrations of drug. Is the dynamic range of the assay only 10-fold, or is it several logs? If the dynamic range is not very large than the conclusion that the mutant virus is more resistant to the drug may not be true. But if negative controls are several logs lower than the positive control, then I agree with their assessment that rF is more insensitive to the drug.

AR: We thank the reviewer for this suggestion and changed the y-axis with the luminescence data to log-scale to better reflect the dynamic range of the assay which covers as discussed above approx. 3 to 3.5 log steps.

Also, I can barely read the numbers on the y-axis, these need to be increased in size.

AR: We agree with the reviewer regarding the readability of the labels and thus increased the font size for the labeling of the y-axis in Fig. 8 h + i.

7. I don't get what is going on with Figure S20c. In the data in S20a-b, the compound at these concentrations had nearly identical effects on rF and rWT in the absence of IFN-g. However, in c, the compound reduced rWT nearly 5-logs while only affecting rF by at most 1-log. The other problem is that if an enzyme dead mutant has no defect without IFN-g then the drug should not impact WT virus replication if it's targeting this enzyme activity (also the Mac1 deletion virus did not have a defect in these cells). Thus, the impact on WT virus would have to be non-specific, which the authors acknowledged in other parts of the paper. But the compounds also did not impact the mutant virus, which is very weird. The only explanation I can come up with is that the rF virus was either not treated with IFN-g while the rWT virus was, or that different amounts of compounds were used for each virus. The authors need to redo this assay or have a logical explanation for the discrepancy in their results.

AR: We thank the reviewer for this careful and important assessment. We agree that the magnitude of the differential effects observed in the original version of Supplementary Fig. 20c appeared difficult to reconcile with the luciferase-based measurements shown in Supplementary Fig. 20a–b and warranted further investigation.

In the original experiment, an additional higher concentration of ST166 **6** had been included experimentally but was not titrated, as the selected concentrations were primarily based on the most

informative range identified in the luciferase assay. Following the reviewer's comment, we re-analysed these original data (shown in Supplementary Fig. 20a) and additionally repeated the infectious titre assay (Supplementary Fig. 20b).

Analysis of the previously unshown higher concentration, together with the repeat experiment, demonstrated that both rWT and rF are inhibited at elevated compound concentrations of ST166 **6**. Although the differential effect was less pronounced in the repeat experiment, rF consistently remained less sensitive to ST166 **6** treatment than rWT.

We therefore agree with the reviewer that the original Supplementary Fig. 20c likely overstated the magnitude of the differential inhibition between the two viruses. To address this, we have replaced the original panel with a revised figure showing both datasets side-by-side, thereby more accurately reflecting the experimental variability and overall trend observed across experiments. We appreciate the reviewer's comment, which prompted us to further validate and refine these findings

8. The data in Fig. S21 is not consistent with a role for Mac1 in maintaining NAD+ levels. While the authors make an observation of lower NAD levels at 2 hpi in the presence of IFN-g and ST166, the difference between that group and the IFN-g only treated samples is likely not significant. Furthermore, viral protein production does not typically begin until 4-6 hpi, so the amount of Mac1 in the cell at that time is either minimal or completely absent. Thus, the conclusions on lines 444-445 should be modified or the experiment should instead be done at about 8 hpi.

AR: We agree with the reviewer that Mac1 expression is expected to be minimal or absent at 2 hpi. To address a potential effect of Mac1 on cellular NAD levels, we repeated the experiment and included an additional later time point (12 hpi), at which Mac1 expression would be expected. While the combined data confirmed both, the reduction in cellular NAD levels following IFN-γ preincubation (at 2 h and 48 h) and the lack of effect of ST166 on cellular NAD levels under any condition, the previously observed differences between IFN-γ–preincubated infected cells and the corresponding ST166-treated samples were not reproducible and are no longer statistically significant, including at 12 hpi. We therefore agree with the reviewer that our previous interpretation overemphasized the data and have revised the manuscript accordingly. The data for 2 hpi and 48 hpi are now shown in the revised Supplementary. Fig. 22. For the reviewer we show the full data set including the 12h time point below-

The revised text now reads:

"In uninfected cells, IFN-γ treatment caused an approximately 40% reduction in NAD levels. SARS-CoV-2 infection had no significant effect on cellular NAD levels at 2 hpi and caused an approximately 30% reduction at 48 hpi. ST166 alone had no significant effect on cellular NAD levels and did not further alter NAD levels in combination with viral infection or IFN-γ preincubation."

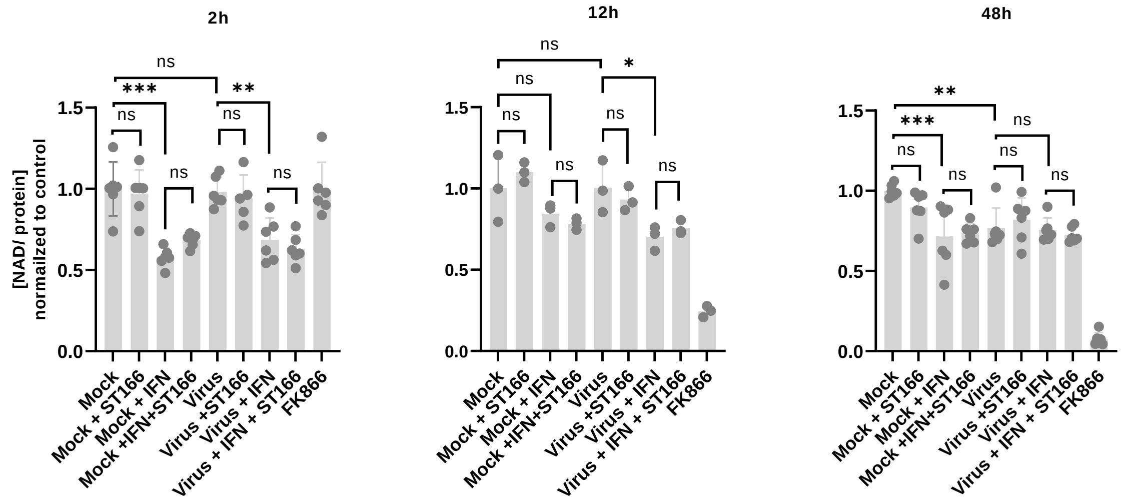

In addition, we removed the former corresponding interpretation in the discussion section (lines 546-551). The removed paragraph was:" Consistently, improvement of pyrophosphate stability in compound **Fehler! Verweisquelle konnte nicht gefunden werden.** resulted in a more pronounced difference between rWT and rF inhibition, suggesting a preferential targeting of Mac1, which is further supported by our observation that ST166 **6** restores NAD^+ depletion induced by IFN-γ upon SARS-CoV-2 infection."

Reviewer #2:

The modifications that have been incorporated in the revised manuscript, including supplementary data, are compelling and present a cohesive and robust case as to the novelty of this new class of pro-drug antiviral compounds and their mechanism of action against an innovative target.

The authors have addressed comprehensively all my comments and concerns. As such, I strongly support its publication.

AR: We thank the reviewer for the positive assessment of our revised manuscript and for recognizing the significance of our findings. We appreciate the reviewer's constructive comments throughout the review process.

Reviewer #3:

The authors have improved the manuscript based on the suggestions and have addressed the concerns well.

AR: We thank the reviewer for his helpful comments and his positive remarks.

I have listed some minor improvement suggestions still below.

Although the connection between what initially appeared to be two separate studies has improved, it could still be strengthened to help guide the reader through the narrative. Because the manuscript presents a large amount of data, establishing a clear storyline and smooth flow is particularly important.

Manuscript: Targeting a viral macrodomain: Design, structure-based optimization and antiviral evaluation of potent inhibitors for Mac1 of SARS-CoV-2

ID: NCOMMS-25-56595A

The discussion of the ADPR analog analysis remains rather lengthy, as many of the described results do not clearly inform the final conclusions. Figure 4 provides a helpful overview of these findings and is appreciated. However, it is currently underutilized and could be integrated more effectively into the discussion. For example, it would be better to refer back to the figure rather than presenting abrupt statements such as "Next, we synthesized β-methyl-GS-441524-DP 4.".

AR: We thank the reviewer for this helpful suggestion. In response, we revised and shortened the discussion of the ADPR analogue analysis to improve focus and readability. In addition, we now integrate Fig. 4 more explicitly throughout the discussion to better connect the analogue designs with the emerging SAR model.

For example, the sentence "Next, we synthesized β-methyl-GS-441524-DP **4**" was revised to:

"Based on our SAR model, which suggests favoured binding of Mac1 towards β-alkylated nucleotide diphosphates (Fig. 4), we synthesized β-methyl-GS-441524-DP **4**."

We believe these changes improve the overall flow of the discussion and clarify how the individual analogue studies contribute to the final conclusions.

The discussion section now acknowledges several potential limitations, but these could be elaborated further. The selectivity analysis is based on only two additional macrodomains, making this aspect relatively limited. This limitation could be more explicitly discussed, as it represents an important area for future work rather than a strength of the current manuscript. This especially as the inhibitors are nucleoside analogs.

AR: We thank the reviewer for raising this important point. We agree that our selectivity analysis is currently limited, as only MacroD1 and MacroD2 were evaluated, and that this should not be overinterpreted as evidence for broad specificity. We have therefore revised the Discussion to more clearly acknowledge this limitation and to frame the current data as providing only an initial indication of selectivity. In addition, we now explicitly note that, given the nucleoside nature of the compounds, broader profiling against additional macrodomains and other host ADP-ribose/NAD-binding proteins will be important future work. At the same time, we retained the structural discussion regarding the potential role of the oxyanion hole in contributing to the observed selectivity against MacroD1 and MacroD2.

The section now reads: „β-Methyl-GS-441524-DP 4 exhibited no detectable inhibition of the human macrodomains MacroD1 and MacroD2 under the conditions tested, suggesting an initial degree of selectivity for Mac1. Although alkylated ADP derivatives also showed some degree of specificity (data not shown), the selectivity of compound **4** likely arises from the GS-441524 nucleoside scaffold. Ni et al. demonstrated that the 1′-cyano group of GS-441524 inserts into a pocket near loop β7-α6, where it interacts with the backbone N–H groups of Phe156 and Asp157, creating a positive dipole that stabilizes the partial negative charge of the cyano group.[21] Schuller et al. referred to this region as the "oxyanion hole", as several anionic fragments were found to bind at this site.[13] In human MacroD1 and MacroD2, a leucine in this oxyanion hole is replaced by aspartate. The resulting electrostatic incompatibility with the cyano group may contribute to the apparent selectivity of β-alkylated GS-441524-DP 4 for Mac1. However, the present selectivity analysis remains limited, as only two human macrodomains were examined. Given the nucleoside analog nature of these compounds, broader profiling against additional macrodomains and other host ADP-ribose/NAD-binding proteins will be

important to more comprehensively assess selectivity and potential off-target effects in future studies."

Finally, several sections contain multiple one-sentence paragraphs. Some rephrasing and merging of these paragraphs would help improve readability and better connect the experimental results with the corresponding interpretations.

AR: We thank the reviewer pointing this out. In response we merged some of the paragraphs and were able to improve the overall flow throughout the manuscript. We believe that these changes improved readability and interpretation of the presented data by the reader.

*Explanation for correction of error and statistics in Fig. 8 d+g:

Reviewing of our datasets we found an error in Fig. 8d (bar chart of AUC) for ST135 - rF (red), which showed mean AUC = 0.05254 and has now been corrected to mean AUC = 0.2457, so that the bar chart is now consistent with the corresponding concentration-response curve.

The statistics for panels d and g were reanalysed. We realized that the original GraphPad Prism implementation for comparing AUCs (based on Gagnon and Peterson, 1998) treats individual datapoints along the dose–response curves as independent observations. We therefore recalculated the AUC separately for each independent experiment and subsequently compared AUC values between groups using unpaired t-tests. This reanalysis yielded qualitatively similar results and did not alter the overall conclusions, although p-values changed quantitatively.

We changed the Legend for Fig 8 accordingly. It now specifically states for panels d and g that AUC was determined from each experiment and now reads: "**(a)** Schematic overview of the recombinant viruses. (b - d) CaLu-3 cells were infected with rWT or rF at MOI=1 and treated with **(b)** ST135 (C_{11}-β-methyl-GS-441524-DP) **5** or (c) ST166 **6** at increasing concentrations. Luciferase reporter activity (RLU) was measured 24 hpi and (d) corresponding area under the curve (AUC) values from each experiment compared by unpaired t-tests. **(e - g)** A549-A/T cells were infected with rWT or rF at MOI=1 and treated with (e) ST166 6 or (f) additionally stimulated with IFN-γ (250 U) prior to infection. RLU and (g) corresponding AUC values were determined and statistically tested as above. **(h, i)** Raw RLU data underlying (e-g) were tested by lognormal ANOVA with Šídák-Holm-corrected post hoc test against prodrug-untreated controls. A dashed line indicates the RLU at 0 hpi. **(j)** CaLu-3 cells were infected with rWT or rF at MOI=1 with or without ST166 **6** (0.5 µM) treatment 16 hpi. Induction of IFNβ and IFITM1 (mRNAs) was calculated at 48 hpi relative to uninfected, untreated cells. Two-way ANOVA with Šídák–corrected post-hoc t-tests of log-transformed data were performed. Data points (n= 3) are represented, means ± SEM are given. ns: not significant, *p ≤ 0.05 **p ≤ 0.01,***p ≤ 0.001, ****p ≤ 0.0001.".

Manuscript: Design, structure-based optimization and antiviral evaluation of potent Inhibitors for the macrodomain Mac1 of SARS-CoV-2

ID: NCOMMS-25-56595-C

Response to reviewers

Once again we would like to thank the reviewers for their comments and constructive cri tiques throughout the revision process which have helped us to significantly improve the manu script.
